# Metaproteomic investigation of functional insight into special defined microbial starter on production of fermented rice with melanogenesis inhibition activity

**DOI:** 10.1371/journal.pone.0241819

**Published:** 2020-11-04

**Authors:** Orrarat Sangkaew, Narumon Phaonakrop, Sittiruk Roytrakul, Chulee Yompakdee

**Affiliations:** 1 Department of Microbiology, Faculty of Science, Chulalongkorn University, Pathumwan, Bangkok, Thailand; 2 Functional Ingredients and Food Innovation Research Group, National Center for Genetic Engineering and Biotechnology, National Science and Technology Development Agency, Klong Luang, Pathumthani, Thailand; Institute for Biological Research "S. Stanković", University of Belgrade, SERBIA

## Abstract

Fermentation of rice grains requires diverse metabolic enzymes to be synchronously synthesized by the microbial community. Although many studies have used a metaproteomic approach to investigate the roles of microorganisms in improving the flavor of fermented foods, their roles in producing compounds with biological activity have not yet been reported. In a previous study the ferment obtained from unpolished black rice (UBR) fermented with a defined microbial starter (De-E11), comprised of *Rhizopus oryzae*, *Saccharomycopsis fibuligera*, *Saccharomyces cerevisiae*, and *Pediococcus pentosaceus*, (fermented UBR; FUBR) showed a strong melanogenesis inhibition activity in B16F10 melanoma cells. Hence, in this study, the roles of these microorganisms in producing the melanogenesis inhibitor(s) in FUBR was investigated using a metaproteomic approach. The melanogenesis inhibition activity of the FUBR liquid (FR-Liq) was found to increase with longer fermentation times. *R*. *oryzae* and *S*. *cerevisiae* were the major hosts of proteins related to the biosynthesis of melanogenesis inhibitor(s) in the FUBR. During fermentation, the enzymes involved in the degradation of UBR and in the carbohydrate metabolic process were identified. These enzymes were associated with the process of releasing of bioactive compound(s) from UBR and the synthesis of organic acids from the microorganisms, respectively. In addition, enzymes involved in the synthesis of some known melanogenesis inhibitor(s) and in the degradation of the melanogenesis stimulator (arsenate) were detected. Varying the combination of microorganisms in the De-E11 starter to produce the FR-Liq revealed that all four microorganisms were required to produce the most potent melanogenesis inhibition activity. Taken together with the metaproteomics results, this suggested that the microorganisms in De-E11 synchronously synthesize the FR-Liq with melanogenesis inhibition activity. In conclusion, this information on the metaproteome in FUBR will increase our understanding of the microbial metabolic modes and could lead to knowledge-based improvements in the fermented rice process to produce melanogenesis inhibitor(s).

## Introduction

Fermented foods are well-known for their nutritional benefits and biological activities [1]. Within Asia, including Thailand, fermentation of rice with a specific traditional microbial starter, such as loogpang, koji, nuruk, and jiuqu, is widely used in the early stages of manufacturing fermented foods, such as rice wine (Sake, Sato, and Makgeolli), Chinese distilled beverage (Baijiu), sweet fermented rice (Khao-Mak), fermented red pepper paste (gochujang), and fermented soybean paste (miso and doenjang) [2–4]. These microbial starters act as enzyme sources for the fermentation process, but the composition of the starter culture affects the quality of the fermented food products [5, 6].

Microorganisms are the most important participants in the fermentation process. Fermentation is performed by mixed microorganisms, in which several microorganisms show symbiotic cooperation [7, 8]. Many reports have demonstrated that fermentation not only enhances the biological activity of the substrate, but can also lead to new biological activities [9–11]. Hence, an in-depth knowledge of the functions of microbial ecosystems is essential to understand the mechanism of the fermentation.

Recently, microbiota research has seen a shift in perspective from taxonomy to function. Among the systematic approaches for the characterization of the function of microbial ecosystems, metaproteomics has the advantage of being able to determine which proteins are expressed in a mixed culture, which makes metaproteomic analysis a powerful tool to better understand the role of a given microbiota in complex samples, such as fermented foods and beverages [12–15]. Several metaproteomics studies have been performed to identify microbial proteins involved in the flavor-formation of fermented foods [14, 16, 17]. However, there are no studies to date using metaproteomics to identify microbial proteins involved in the biological activities of fermented food.

Melanogenesis is a physiological process that results in the synthesis of melanin pigments. Although melanin plays an important role in skin protection from harmful effects caused by UV ray, the overproduction of melanin can lead to hyperpigmentation disorders, such as melisma and freckles [18]. Hence, melanogenesis inhibitors are in big demand for the treatment of hyperpigmentation disorders [19]. Several reports have indicated that some fermented products have an effective function as a melanogenesis inhibitor for reducing melanin, such as fermented soy milk, fermented *Angelica tenuissima*, and fermented red ginseng [20–22].

The fermented rice in Thailand called “Khao Mak”, which is produced from rice fermented with Thai traditional starters, or so called “*loogpangs*”, has been reported to possess many biological activities, such as anti-osteoporosis, anti-mutagenic, and anti-cancer activities as well as promoting growth development of malnutritioned children, and so it could be utilized as a health-functional food or pharmaceutical agent [23–25]. Hence, fermented rice is an interesting product to determine its melanogenesis inhibition activity.

In a previous study, a combination of varying rice cultivars and varying sources of the Thai traditional starter, *loogpang*, was used to produce fermented rice and screened for melanogenesis inhibition activity in B16F10 melanoma cells [26]. The combination of *loogpang* E11 and unpolished black rice (UBR) was selected for production of a fermented rice product having the most potent melanogenesis inhibition activity [26]. It was also reported that the liquid obtained from fermented UBR (FUBR), called FR-Liq, using a defined starter mixture of microbes isolated from the selected *loogpang* E11, De-E11 [containing *Rhizopus oryzae*, *Saccharomycopsis fibuligera*, *Saccharomyces cerevisiae*, and *Pediococcus pentosaceus*] (FUBR) for 12 d showed a similar anti-melanogenesis activity as that from the FUBR using the original *loogpang* E11 [26]. Therefore, to understand the role of these microorganisms in the FUBR that are involved in production of melanogenesis inhibitor(s), the microbial proteins in the FUBR were investigated using metaproteomics analysis. This study will improve our knowledge of fermented rice for the production of effective melanogenesis inhibitor(s).

## Materials and methods

### Fermentation of UBR and sample collection

The FUBR was prepared as previously reported [26]. The UBR or Hom nin rice, used as the raw material for the fermentation, was purchased from Green Niche Rice, Thailand. The UBR (200 g) was mixed with 400 mL of water and autoclaved at 121°C for 15 min. After that, the cooked rice was mixed with the defined De-E11 microbial starter which isolated from *loogpang* E11 [26], and is comprised of *R*. *oryzae* strain E1101, *S*. *fibuligera* strain E1102, *S*. *cerevisiae*, strain E1103 and *P*. *pentosaceus* strain E1104 (deposited at Microbial Culture Collection, Department of Microbiology, Faculty of Science, Chulalongkorn University (MSCU), Thailand) at 1 x 10^4^, 2 x 10^4^, 1 x 10^3^ and 3 x 10^8^ colony forming units (CFU)/g, respectively, and is consistent with amount enumerated from the original *loogpang* E11. The sample was divided into five bottles, lid attached, and incubated at 30°C for different fermentation times (0, 3, 6, 9, and 12 d). At each time point a sample was collected and centrifuged at 11,000 ×g for 15 min to separate the liquid (FR-Liq) and sediment (FR-Sed) phases. Both phases were then stored separately at -80°C for further study.

To determine the microorganisms in the defined microbial starter De-E11 that play a role in the production of the FR-Liq with melanogenesis inhibition activity, the cooked rice was mixed with (i) *R*. *oryzae* (R), (ii) *R*. *oryzae* and *S*. *cerevisiae* (Sc) (R + Sc), (iii) *R*. *oryzae*, *S*. *cerevisiae*, and *P*. *pentosaceus* (P) (R + Sc + P), or (iv) *R*. *oryzae*, *S*. *cerevisiae*, *P*. *pentosaceus*, and *S*. *fibuligera* (Sm) (R + Sc + P + Sm). The samples were incubated at 30°C for 12 d, then the FR-Liq was harvested and determined for melanogenesis inhibition activity in the B16F10 cell line.

### Melanin content assay

Cellular melanin content was measured as previously described [27]. The B16F10 melanoma cell line (ATCCCCL-6475™) was cultured in complete medium [CM; DMEM supplemented with 10% (v/v) fetal bovine serum, penicillin (100 U/mL), and streptomycin (100 mg/mL)] in six-well plates (5 x 10^4^ cells/well) and incubated for 24 h at 37°C under a humidified 95% (v/v) air, 5% (v/v) CO_2_ atmosphere. The cells were then treated with 5% (v/v) of FR-Liq and incubated in CM as above for 72 h. After incubation, the cells were harvested and solubilized in 1 N NaOH at 60°C for 60 min, and the absorbance of the cell suspension was measured by spectrophotometer at 405 nm (OD_405_). The melanin content was expressed as the relative residual melanin (%) from the following equation: relative residual melanin (%) = [(A÷B) ÷ (C÷D)] x 100, where A and C are the OD_405_ values of the treated and untreated cells, respectively, and B and D are the protein concentrations of the treated and untreated cells, respectively.

### Extraction of total proteins from the FUBR

Total protein was isolated from the FR-Sed as reported [16] except with some modifications. Briefly, 5 g of FR-Sed were suspended in extraction buffer [50 mM Tris-HCl buffer pH 7.5, 100 mM KCl, 50 mM ethylenediaminetetraacetic acid, 5 mM dithiothreitol (DTT), 2% (w/v) polyvinylpolypyrrolidone, and 30% (w/v) sucrose] and sonicated on ice for 10 min. The samples were then centrifuged at 4,000 × *g* at 4°C for 5 min, where after the supernatants were collected and the pellets were discarded. Tris-buffered phenol was added to the samples at a 1:1 (v/v) ratio and shaken on ice for 1 h prior to centrifugation at 12000 × *g* for 15 min at 4 °C. The upper phenol phase was transferred and extracted with an equal volume of extraction buffer. Extracted proteins were precipitated from the phenol phase by adding 5 volumes of 100 mM ammonium acetate in 100% (v/v) methanol and incubated at −20 °C overnight. The proteins were then collected by centrifugation at 12000 × *g* for 15 min at 4 °C. The protein pellets were washed twice with cold acetone, dried, and solubilized in 1% (w/v) sodium dodecyl sulphate (SDS).

The protein in the FR-Liq was prepared using the methanol/chloroform protein precipitation method. For this, 150 μL liquid sample was mixed with 600 μL methanol, 150 μL chloroform, and 450 μL of water. After centrifugation at 14,000 x g for 5 min at 4°C, the upper phase was discarded. Next, 650 μL methanol was added, mixed, and centrifuged at 14,000 x g for 5 min at 4°C. The protein pellet was dried and solubilized in 1% (w/v) SDS.

The proteins obtained from the FR-Liq and FR-Sed were stored separately at −80°C for later use. The concentration of protein samples was quantified according to the Bradford assay (1976) using bovine serum albumin as the protein standard.

### Liquid chromatography-tandem mass spectrometry (LC/MS-MS) analysis

Each 5 μg protein sample (proteins obtained from FR-Liq and FR-Sed at different times of fermentation) was reduced using 5 mM DTT in 10 mM ammonium bicarbonate at 60°C for 1 h and alkylated using 15 mM iodoacetamide in 10 mM ammonium bicarbonate at room temperature for 45 min in the dark. The protein samples were then mixed with 50 ng/μL of sequencing grade trypsin (1:20 ratio) (Promega, Germany) and incubated at 37°C overnight. The tryptic peptide samples were injected into an Ultimate3000 Nano/Capillary LC System (Thermo Scientific, UK) coupled to a HCTUltra LC-MS system (Bruker Daltonics Ltd; Hamburg, Germany).

Briefly, peptides were enriched on a C18 Pepmap 100, 5 μm, 100 A (Thermo Scientific, UK) μ-Precolumn (300 μm i.d. x 5 mm), and then separated on a 75 μm I.D. x 15 cm column packed with Acclaim PepMap RSLC C18, 2 μm, 100Å, nanoViper (Thermo Scientific, UK). Solvent A and B were 0.1% (v/v) formic acid in water and 0.1% (v/v) formic acid in 80% (v/v) acetonitrile (ACN), respectively. A gradient of 5–55% solvent B was used to elute the peptides at a constant flow rate of 0.30 μL/min for 30 min. The MS and MS/MS spectra were obtained in the positive-ion mode over the range (m/z) 150–2200. Peptide fragment mass spectra were acquired in data-dependent AutoMS (2) mode with a scan range of 300−1500 *m*/*z*, three averages, and up to five precursor ions selected from the MS scan 50−3000 *m*/*z*. The LC-MS analysis of each sample was done in triplicate.

### Bioinformatics and data analysis

DecyderMS was used to quantify the proteins in individual samples [28]. Protein identification with Mascot standard settings was performed: maximum of two miss cleavages, mass tolerance of 0.6 Da for the main search, trypsin as the digesting enzyme, carbamidomethylation of cysteine as the fixed modification, and oxidation of methionine as the variable modifications. Only proteins with at least one unique peptide were considered as being identified and used for further data analysis. The bacterial protein database downloaded from NCBI was used as the search FASTA file. The level of proteins in each sample was expressed as a log_2_ value.

Gene Ontology (GO) and biological process (BP) annotations for the identified proteins were assigned according to those reported in the uniport database (http://www.uniprot.org). Data visualization of the melanin content analysis was achieved using the GraphPad Prism by one-way analysis of variance (ANOVA), followed by Tukey’s multiple comparison test. A *p*-value of < 0.05 was accepted as significant.

### Analysis of some major compounds in FUBR

Fermented rice samples collected at day O (Un-FR) and the liquid part at day 12 (FR-Liq) were lyophilized prior to resolvation in water. The samples were then subjected to high-performance liquid chromatography (HPLC) using an Agilent 1200 series HPLC system. Separation was completed using the SunFire C18 OBD Prep Column, 10 μm, 19 x 250 mm. The mobile phase consisted of mobile phase A (85% water) and mobile phase B (15% ACN) at, a flow rate of 15 ml/min. The gradient elution profile was 0 min, 15% B; 18 min, 80% B; 33 min, 80% B; 35 min, 100% B. Some major compounds found only in the FR-Liq but not in the Un-FR were further purified and identified by mass spectrometry (MS) (Bruker micrOTOF spectrometer, Bruker, Karlsruhe, Germany) and nuclear magnetic resonance spectrometry (NMR) (Bruker AV500 spectrometer, Bruker, Karlsruhe, Germany). Each sample was analyzed in triplicate.

## Results and discussion

### Melanin content reduction by the FUBR liquid (FR-Liq)

Melanin is a dark skin pigment that can lead to perceived esthetic problems [18]. In a previous study, the FR-Liq fraction obtained from UBR fermented with the De-E11 starter for 12 d was found to be able to reduce the melanin content in cultured B16F10 melanoma cells via a reduced tyrosinase activity and expression level of melanogenesis related genes or proteins [26].

**Fig 1:**
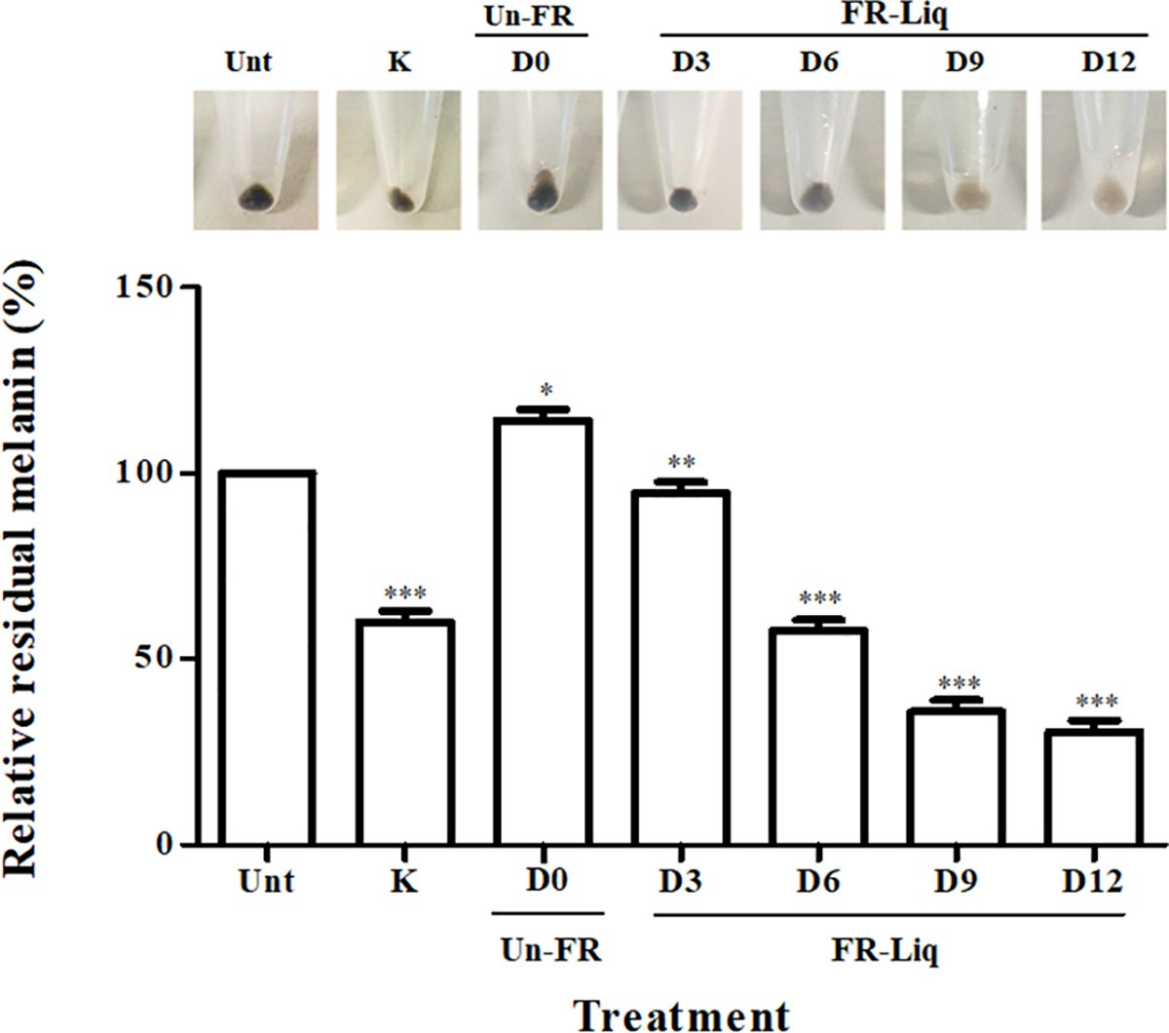
Reduction of the melanin content in B16F10 melanoma cells by FR-Liq from different fermentation times. B16F10 melanoma cells were treated with 1 mM kojic acid (K) as a positive control and FR-Liq from different fermentation times (0, 3, 6, 9, and 12 d). Data are shown as the mean ± SEM from three independent experiments performed in triplicate. Statistically significant differences between each FR-Liq and the fermented rice at day 0 (Un-FR) and between K and the Un-FR at day 0 (D0) compared with untreated cells (Unt) are indicated by *p < 0.05, **p < 0.01, and ***p < 0.001.

In this study, the reduction of melanin content in B16F10 melanoma cells by FR-Liq produced from UBR fermentation at different fermentation times (0, 3, 6, 9, and 12 d) was evaluated. The protein from rice grains with no fermentation (Un-FR, day 0), the FR-Sed at day 0 was extracted using the water extraction method as previously described [29] and used as the unfermented control. The FR-Liq samples collected at day 3, 6, 9, and 12 significantly decreased the melanin content in B16F10 melanoma cells compared to that in the untreated (Unt) cells and the degree of melanogenesis inhibition increased with longer fermentation times. Whereas the unfermented UBR at day 0 (Un-FR) showed a significant increase in the melanin content compared to that in the Unt cells (Fig 1). Thus, the fermented products obtained from the FUBR play important role(s) in inhibiting melanogenesis in B16F10 melanoma cells and the degree of this melanogenesis inhibition increased with longer fermentation times (Fig 1).

### Metaproteomic analysis of the FUBR

The in-situ protein analysis at the community level provides information on the metabolic processes, thus helping to explain how the microorganisms in the De-E11 starter influenced the production of compounds with melanogenesis inhibition activity in the fermented rice.

**Fig 2:**
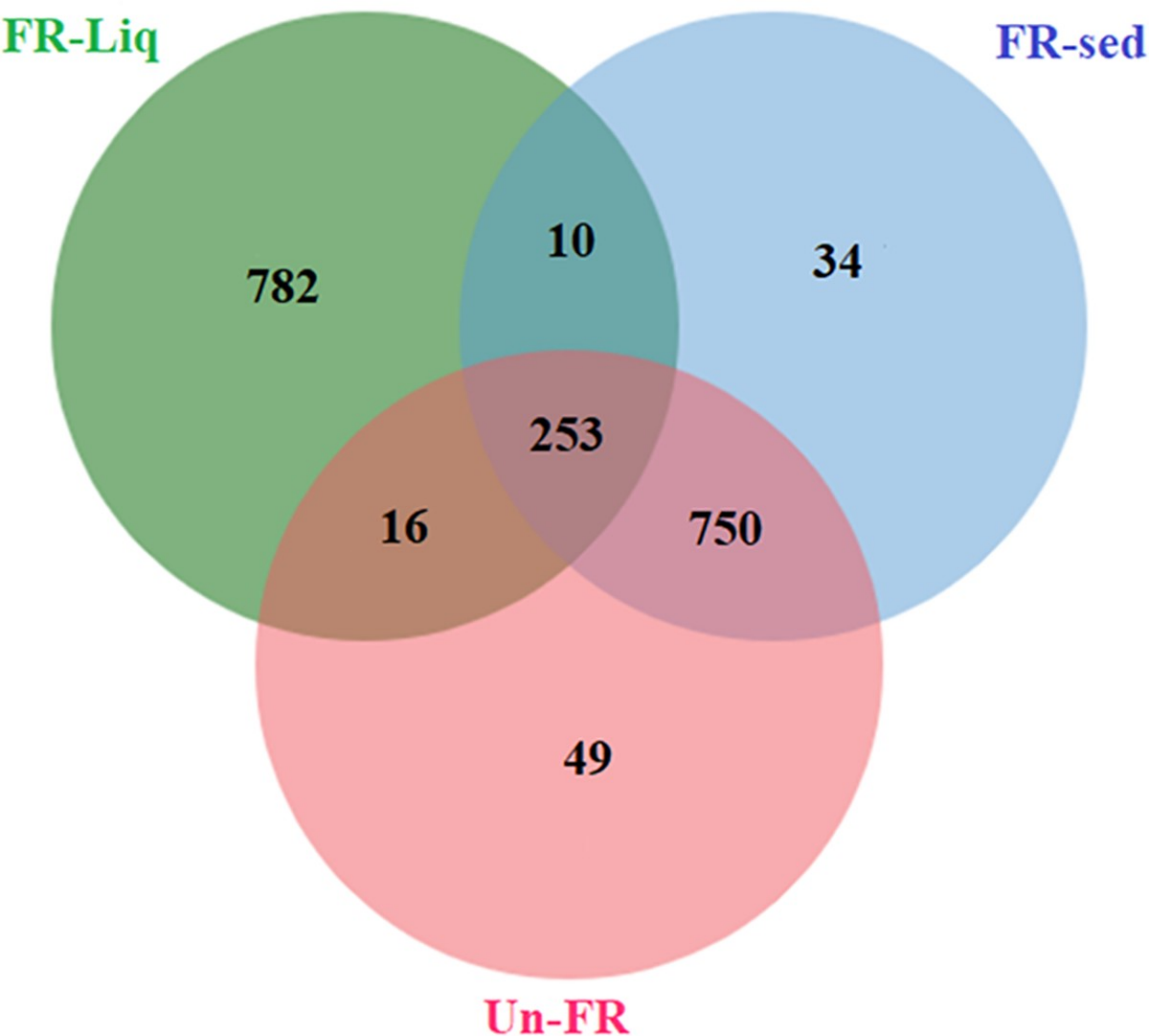
Venn diagram showing the overlap of identified proteins from the FR-Liq, FR-Sed, and Un-FR. The identified proteins belonging to *R*. *oryzae*, *S*. *fibuligera*, *S*. *cerevisiae*, and *P*. *pentosaceus* from the FR-Liq, FR-Sed, and Un-FR were input to a Venn diagram.

The metaproteomes in the FUBR at different fermentation times were evaluated using LC-MS/MS analyses. Proteins produced by the microorganisms in the De-E11 starter during the fermentation of UBR were then investigated. All proteins obtained from the FR-Liq, FR-Sed, and Un-FR were input to a Venn diagram [30], where 782 proteins were identified as being only from the FR-Liq, 34 proteins only from the FR-Sed, and 49 proteins from Un-FR (Fig 2). Moreover, the identified proteins found in both the FR-Sed and Un-FR shared more common proteins (1,003 proteins) than those found in both the FR-Liq and the Un-FR (269 proteins) indicating that more unique identified proteins were found in the FR-Liq than in either the FR-Sed or Un-FR. This result is consistent with a previous study, which found that the FR-Liq, but not FR-Sed and Un-FR, contained the melanogenesis inhibition activity [26].

**Fig 3:**
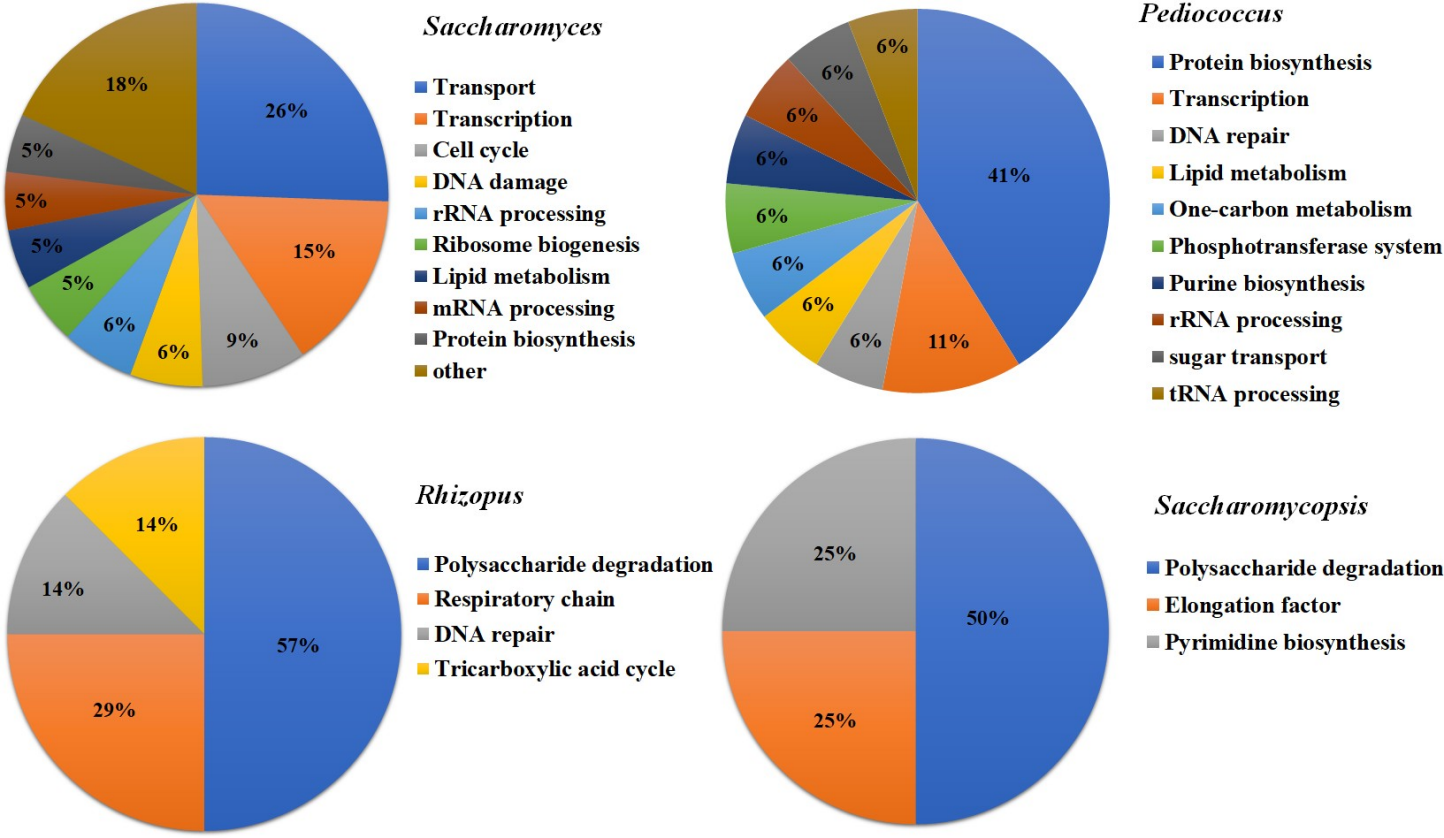
The biological process distribution of identified proteins from the De-E11 starter. The identified proteins from *R*. *oryzae*, *S*. *fibuligera*, *S*. *cerevisiae*, and *P*. *pentosaceus* in the FUBR were classified for their biological process according to Uniprot annotation system.

To analyze the proteins in the fermentation process, the 1,845 identified proteins that were uniquely found during the fermentation process in FR-Liq and FR-Sed were then subjected to further bioinformatic analysis (S1 Table). According to the Uniprot annotation system (http://www.uniprot.org), the 1,845 identified proteins in FUBR were classified based on their biological process (Fig 3). The results showed that the main biological activity of proteins synthesized by *S*. *cerevisiae* and *P*. *pentosaceus* were involved in molecular transport and protein biosynthesis, respectively. In addition, both *R*. *oryzae*, and *S*. *fibuligera* were involved in polysaccharide degradation (Fig 3).

### Proteins related to the biosynthesis of melanogenesis inhibitor(s) in the FUBR

Bioactive compounds, such as phenolic compounds, present in plants can be found in the vacuoles of plant cells and bound with cell wall structures [31, 32]. Fermentation is known to lead to complete breaking down of the plant cell wall resulting in the release of several plant bioactive compounds. Several studies have reported that fermentation influences the profile of phenolic compounds in various plant sources or during the fermentation of plant sources [33].

**Fig 4:**
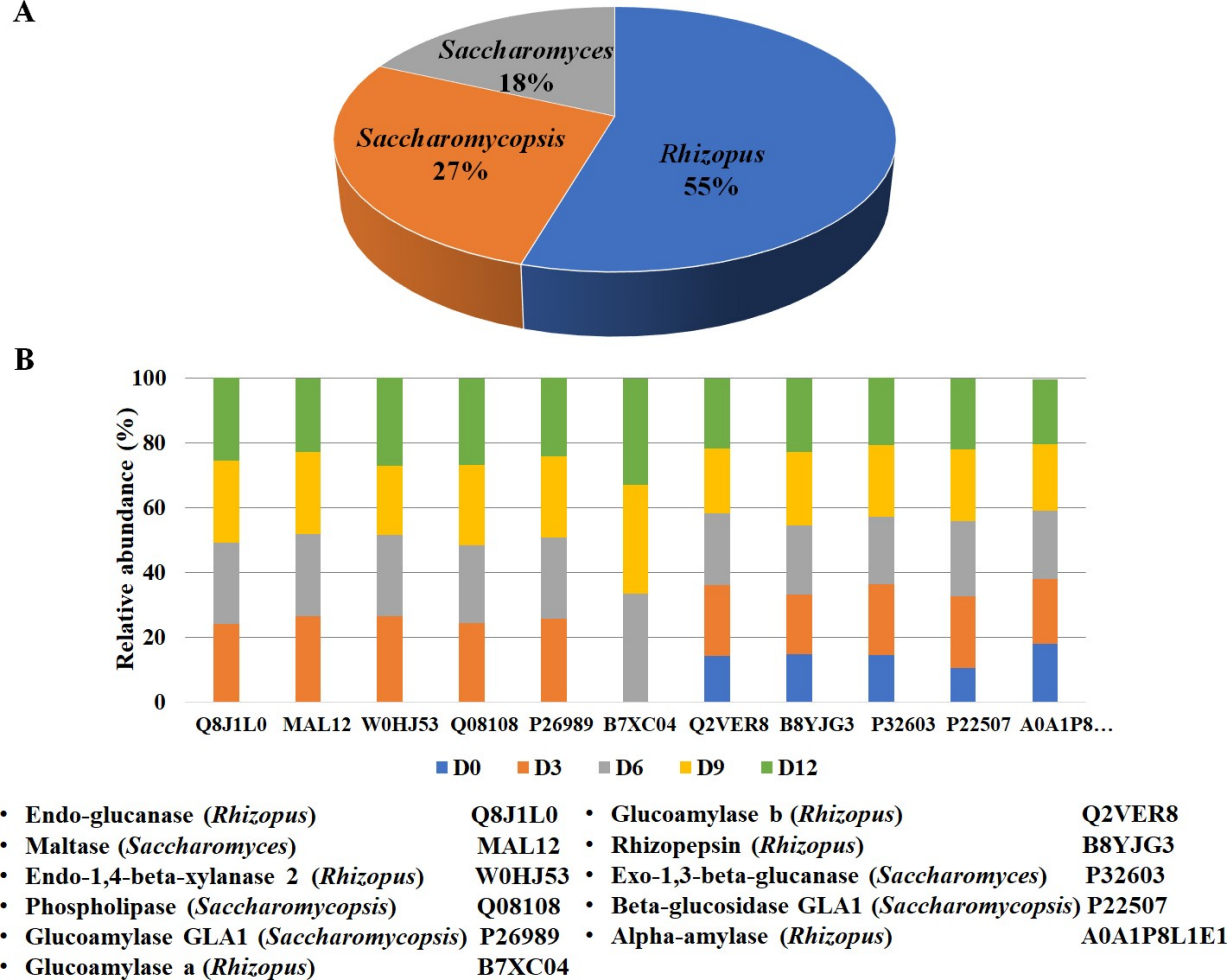
The identified proteins in the FUBR involved in the degradation of rice grain composition. (A) Distribution of proteins involved in the degradation of rice grain composition using the defined microbial starter community, and (B) the abundance of these proteins at different fermentation times.

Moreover, protein degradation in rice grains may yield peptides with a melanogenesis inhibitory activity [34]. To gain more knowledge on the roles of microorganisms in the De-E11 starter that are involved in melanogenesis inhibitor synthesis, in this study the proteins or enzymes possibly involved in the degradation of various components in rice grains, such as xylan, cellulose, starch, protein and lipid, which could be produced by the microbial community during the fermentation process and lead to the release of bioactive compounds into the FR-Liq fraction, were analyzed. Among the identified proteins, 11 proteins were related to degradation process and *R*. *oryzae* was the major source of rice degrading enzymes (Fig 4A). This result was consistent with previous reports that fungi were the major host for enzymes related to substrate degradation [16, 17, 35]. Fig 4B shows the identified proteins involved in the degradation of carbohydrate, protein, and lipid in the FUBR. They were endo-glucanase, maltase, endo-1,4-beta-xylanase, phospholipase, glucoamylase GLA1, glucoamylase a, glucoamylase b, rhizopepsin, exo-1,3-beta glucanase, beta-glucosidase, and alpha-amylase. Six out of these 11 enzymes could be observed during the fermentation process, whereas the level of four enzymes increased during the fermentation process. Recently, similar enzymes involved in polysaccharide degradation were found in the microbial starter of a traditional Chinese beverage, which was studied using a metatranscriptomic approach [35]. Thus, the rice fermentation process led to the *de novo* synthesis or increasing level of the enzymes. Although alpha-amylase did not change in abundance during fermentation, the enzyme activity might be improved by several factors in the fermentation process with increasing fermentation times [36].

**Fig 5:**
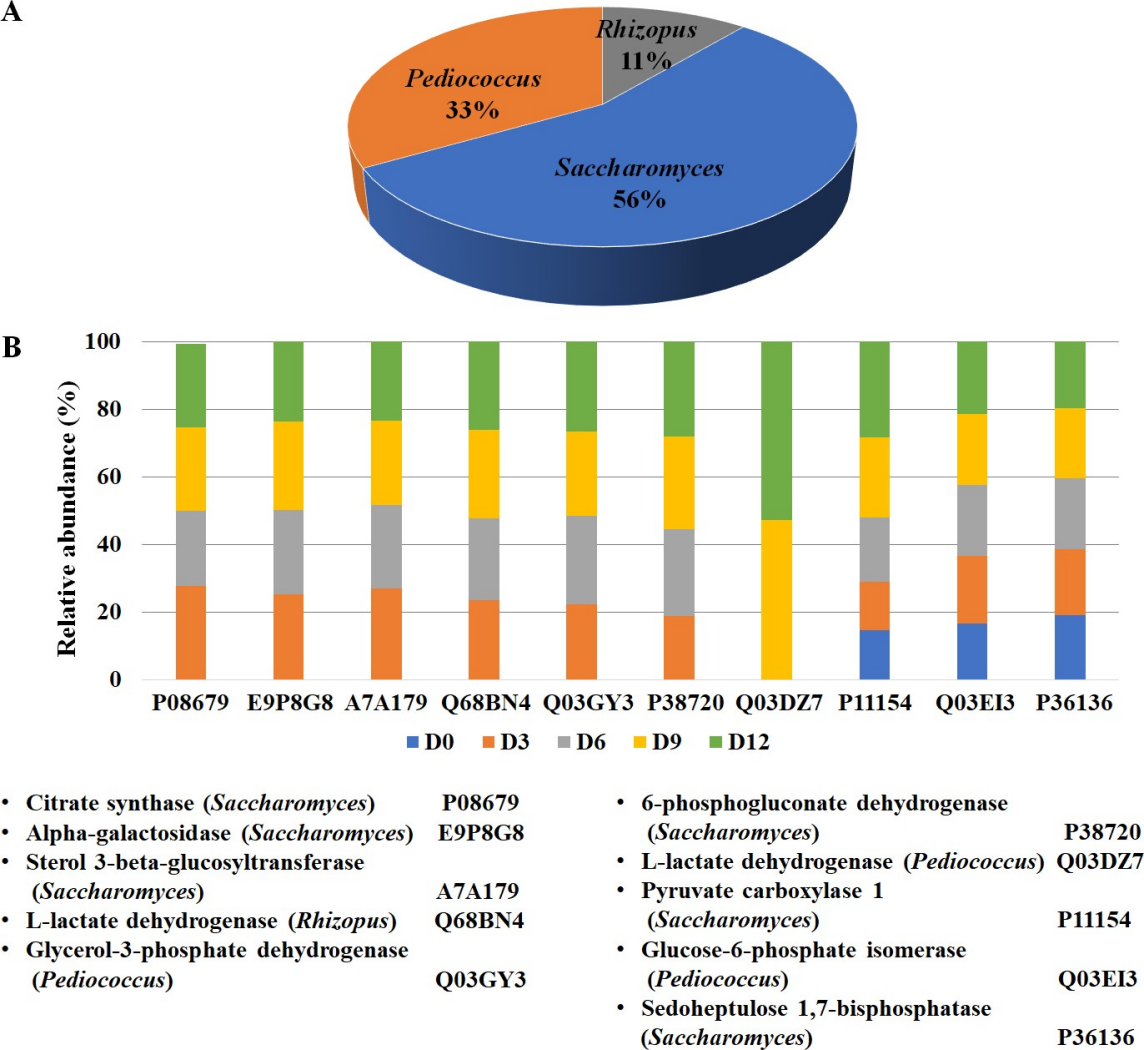
Proteins identified in the FUBR involved in carbohydrate metabolism based on gene ontology. (A) The distribution of proteins involved in carbohydrate metabolism in the De-E11 starter community and (B) their abundance at different fermentation times.

Not only are bioactive compounds obtained from degraded rice grains, but the microbial community themselves can also synthesize a broad variety of bioactive compounds. Carbohydrate metabolism is a fundamental biochemical process that can generate many metabolites, including organic compounds [37, 38], some of which may have biological activities. As shown in Fig 5, ten identified proteins were classified as playing a role in carbohydrate metabolism. Their expression levels increased during the fermentation process (Fig 5B), where seven of the ten enzymes were only found during the fermentation process, and the level of pyruvate carboxylase and glucose-6-phosphate isomerase increased during the fermentation process. These results were consistent with a previous report that the enzymes involved in carbohydrate metabolism were highly expressed in the fermentation process [35].

However, the level of sedoheptulose 1,7-bisphosphatase did not change after fermentation. In accord, it was previously reported that under conditions where NADPH is in demand, such as cell growth, there was no change in the transcript expression level of sedoheptulose-1,7 biphosphatase, an enzyme that is involved in the conversion of glycolytic intermediates into ribose-5-phosphate without the production of NADPH (riboneogenesis) [39]. Moreover, the results showed that these proteins were produced by *S*. *cerevisiae*, the major source of proteins involved in carbohydrate metabolism (Fig 5A).

**Fig 6:**
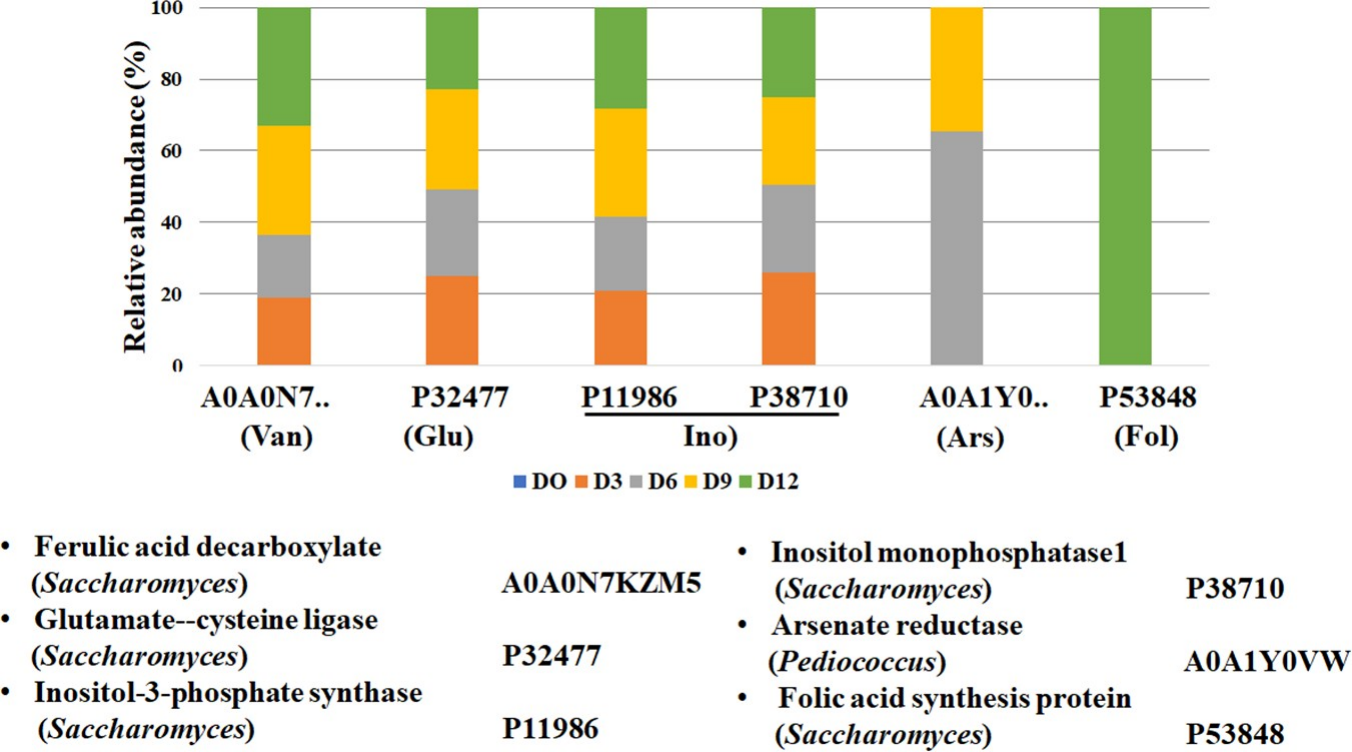
Proteins identified involved in the production of melanogenesis inhibitors and melanogenesis stimulator degrading enzymes. The abundance of these proteins in the FUBR at different fermentation times was analyzed. Van, Glu, Ino, Ars, and Fol represent vanillic acid, glutathione, myo-inositol, arsenate, and tetrahydrofolate, respectively.

In addition, the enzymes involved in biosynthesis of some metabolites that have been reported to have a melanogenesis inhibitory activity were analyzed. Ferulic acid decarboxylase, glutamate-cysteine ligase, and folic acid synthesis were found to be associated with the synthesis of vanillic acid, glutathione, and tetrahydrofolate, respectively [40–43]. Inositol-3-phosphate synthase and inositol monophosphatase 1 were related to myo-inositol synthesis [44]. These compounds have been reported to reduce melanin synthesis [45–48]. Besides, the fermentation process did not exclusively synthesize bioactive compounds, but also decreased some components. The enzyme involved in the reduction of arsenate (arsenate reductase) was detected (Fig 6). Arsenate, which is naturally present in the environment, has been found at higher levels in black rice than in white rice [49]. Long-term exposure to arsenate has been linked to increased skin pigmentation [50]. Previous studies have reported that these known melanogenesis inhibitors and melanogenesis-stimulator degrading enzymes could be produced by microorganisms and fermentation [51–57].

The metaproteomic analysis showed an alteration in the abundance of six enzymes related to not only known melanogenesis inhibitors but also to melanogenesis-stimulator degradation during the fermentation process (Fig 6). All six enzymes were found during the fermentation process.

### Synchronous fermentation process by the defined microbial starter De-E11 on melanogenesis inhibition

The metaproteomic results indicated that *R*. *oryzae* and *S*. *cerevisiae* were the major sources of microbial enzymes in FUBR with melanogenesis inhibition activity (Figs 4 and 5, respectively). Hence, to confirm the metaproteomic results, the FR-Liq obtained from UBR fermented with *R*. *oryzae* and *S*. *cerevisiae* and different combinations of the microorganisms in the De-E11 starter were determined for melanogenesis inhibition activity in B16F10 melanoma cells.

**Fig 7:**
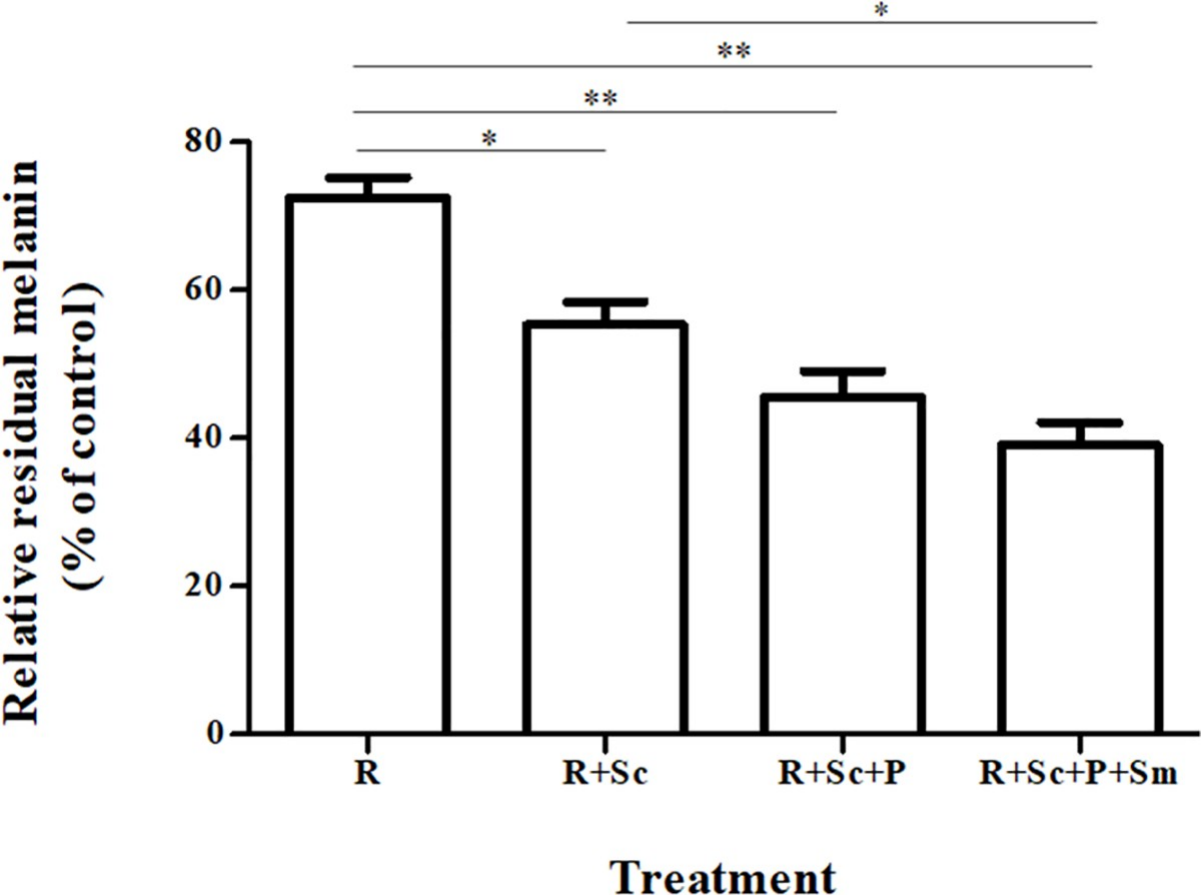
Melanogenesis inhibition activity of the FR-Liq from different combinations of microorganisms in the De-E11 starter. B16F10 melanoma cells were treated with water (as control) or FR-Liq from different combinations of the defined microorganisms in the De-E11 starter. R, Sc, P and Sm represent *R*. *oryzae*, *S*. *cerevisiae*, *P*. *pentosaceus*, and *S*. *fibuligera*, respectively. Data are expressed as a percentage of the untreated control and presented as the mean ± SEM from three independent experiments performed in triplicate. Statistically significant differences between each FR-Liq are indicated by *p < 0.05 and **p < 0.01.

The results revealed that melanogenesis inhibition activity of the FR-Liq obtained from different combinations of the microorganisms in the De-E11 starter showed a significant difference, and were ranked from the lowest to the highest activity as: R < R + Sc < R + Sc + P < R + Sc+ P + Sm, respectively, (Fig 7). Although the activity from FR-Liq obtained from R+Sc+P was not significantly different from that obtained from R + Sc + P + Sm, it was numerically lower. Moreover, the activity of the FR-Liq obtained from R + Sc, which were identified as the two major microorganisms from metaproteomics results (Figs 4 and 5), was significantly different (lower) from that obtained from R + Sc + P + Sm. These results indicated that each microorganism in the De-E11 starter synchronously synthesized the FR-Liq with potent melanogenesis inhibition activity. To obtain FR-Liq with the highest melanogenesis inhibition activity, all four of the microorganisms were required for the fermentation process.

### Analysis of some major melanogenesis inhibitors in FUBR

**Fig 8:**
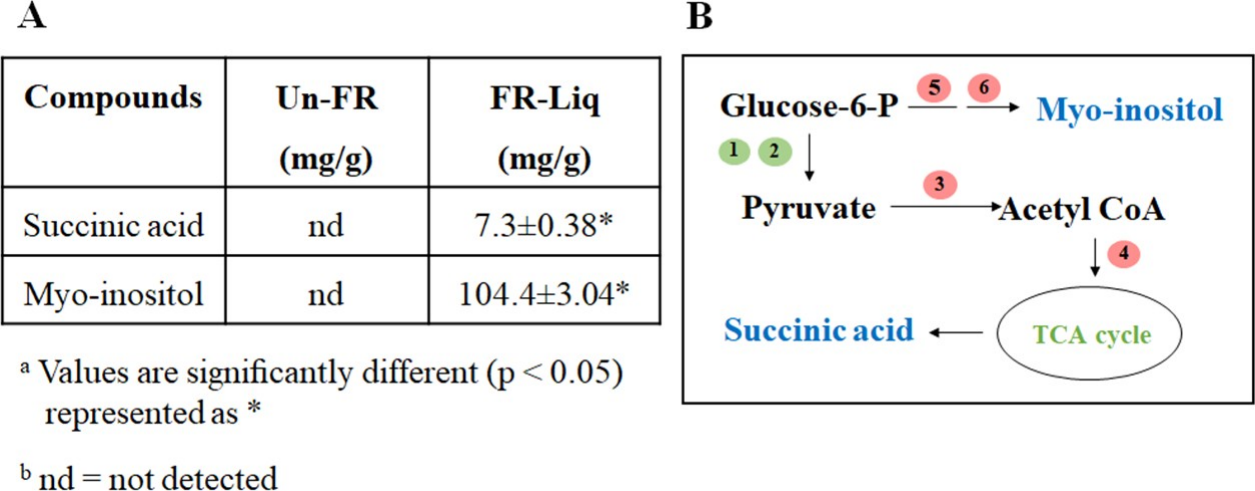
Representatives of major compounds containing anti-melanogenesis inhibition activity in the FUBR. (A) Analysis of the major compounds in FUBR. The data are represented as the mean ± SD, and all values represent triplicate results. (B) Proteins identified in FUBR that were possibly involved in biosynthesis of the representative major compounds succinic acid and myo-inositol. No. 1–6 represent enzymes detected by metaproteomics. 1: glycerol-3-phosphate dehydrogenase, 2: glucose-6-phosphate isomerase, 3: pyruvate carboxylase, 4: citrate synthase, 5: inositol-3-phosphate synthase, and 6: inositol monophosphatase 1, respectively. Red and green circles represent enzymes from *S*. *cerevisiae* and *P*. *pentosaceus*, respectively.

To verify the metaproteomic results, we aimed to detect some major water-soluble compounds that were only found in FR-Liq. The fermented rice samples at day 12 (FR-Liq) and at day 0 (Un-FR) were lyophilized prior to rehydration with water and subjected to HPLC-MS analysis. Succinic acid and myo-inositol, as confirmed by NMR analysis, were two representative major compounds that were only found in the FR-Liq and not in the Un-FR (Fig 8A). Both succinic acid and myo-inositol have previously been reported to exhibit melanogenesis inhibiting activity [45, 58]. The results were consistent with the metaproteomics data (summarized in Fig 8B). Glucose-6-phosphate could be converted to succinic acid by carbohydrate metabolic enzymes, such as glycerol-3-phosphate dehydrogenase, glucose-6-phosphate isomerase, pyruvate carboxylase, and citrate synthase (Fig 5B). In addition, glucose-6-phosphate could be also converted to myo-inositol by inositol-3-phosphate synthase and inositol monophosphatase 1 (Fig 6). Other less polar chemical compounds, including polyphenols, which are known as melanogenesis inhibitors were not detected in this study because the FR-Liq was not extracted with a low polarity solvent prior to chemical analysis.

**Fig 9:**
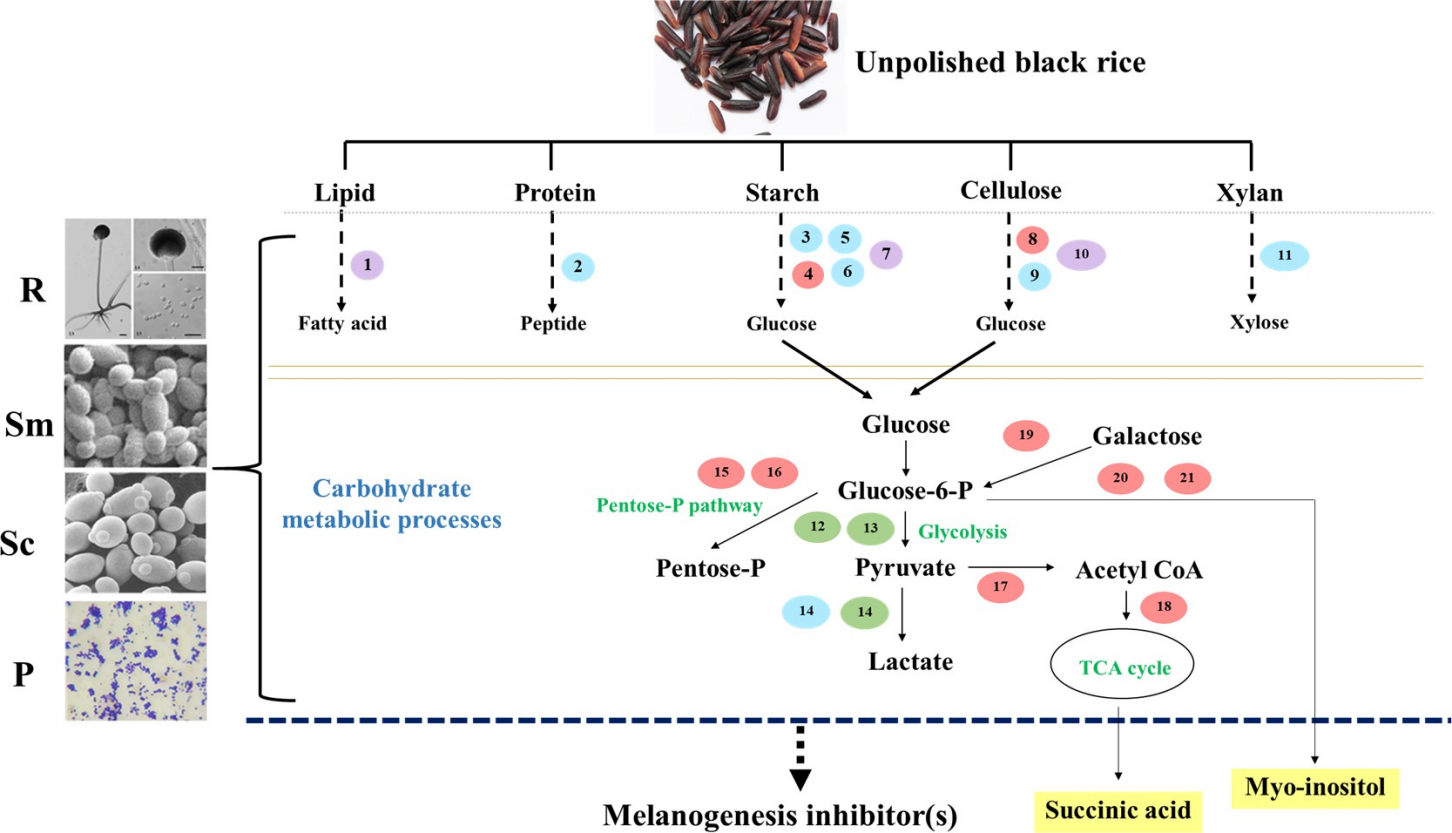
Schematic diagram summarizing the fermentation process inferred from the metaproteomic data. The process begins with UBR grain degradation to carbohydrate metabolism and yield compounds containing anti-melanogenesis inhibition activity. The abbreviations R (blue circle), Sc (red circle), Sm (purple circle), and P (green circle) represent *R*. *oryzae*, *S*. *cerevisiae*, *S*. *fibuligera*, and *P*. *pentosaceus*, respectively. No. 1–21 represent microbial enzymes in the FR-Liq detected by metaproteomics. 1: phospholipase, 2: rhizopepsin, 3: alpha-amylase, 4: maltase, 5: glucoamylase a, 6: glucoamylase b, 7: glucoamylase, 8: exo-1,3-beta-glucanase, 9: endo-glucanase, 10: beta-glucosidase, 11: endo-1,4-beta-xylanase, 12: glycerol-3-phosphate dehydrogenase, 13: glucose-6-phosphate isomerase, 14: lactate dehydrogenase, 15: 6-phosphogluconate dehydrogenase, 16: sedoheptulose 1,7-bisphosphatase, 17: pyruvate carboxylase, 18: citrate synthase, 19: alpha-galactosidase, 20: inositol-3-phosphate synthase and 21: inositol monophosphatase, respectively. Yellow boxes represent some major water-soluble compounds with melanogenesis inhibition activity detected in this study.

Overall, the role of each microorganism in the De-E11 starter could be inferred as follows: during fermentation of UBR by the defined microbial starter, both *R*. *oryzae* and *S*. *cerevisiae* were the main enzyme producers to catalyze degradation of UBR (Fig 4A) and carbohydrate metabolic processes (Fig 5A), respectively. *S*. *fibuligera* may play a role in UBR degradation (Fig 4) while enzymes from *P*. *pentosaceus* regulate the carbohydrate metabolic process, especially in the biosynthesis of lactic acid (Fig 5), another known melanogenesis inhibitor [59]. This overview is summarized as a schematic diagram related to rice degradation and carbohydrate metabolic processes in Fig 9. First, various components in the rice grain, such as xylan, cellulose, starch, protein, and lipid, were degraded into monomers. Moreover, this process leads to the release of several compounds in the UBR. Next, the microorganisms in the De-E11starter can utilize these monomers from the carbohydrate metabolism and generate their metabolites (such as succinic acid and myo-inositol). These combined metabolic processes of the microorganisms in FUBR contributed to the production of diverse metabolic compounds, some of which may enhance the melanogenesis inhibition bioactivity of the FUBR. Moreover, the condition of the fermentation process and fermentation time also contributed to produce the bioactive compounds with melanogenesis inhibition activity (Figs 1, 4B, 5B and 6) [60, 61].

This metaproteomic information increases our understanding of microbial metabolic modes and leads to knowledge-based improvements in fermented rice production to obtain effective melanogenesis inhibition. The information obtained can help improving the growth rate of the microorganisms in the fermentation process by optimizing the fermentation condition (such as aeration, temperature) to suit the microorganisms that synchronously function in fermentation process, as proposed in Fig 9. This could lead to an increased product yield or accelerate the fermentation process.

## Supporting information

S1 Table Listed of identified proteins from the defined microbial starter in the fermented rice. (DOCX)
