# Supplementary material for: Metaproteomic investigation of functional insight into special defined microbial starter on production of fermented rice with melanogenesis inhibition activity
Source: PLoS One. 2020 Nov 4;15(11):e0241819. doi: 10.1371/journal.pone.0241819 (PMC7641363; doi:10.1371/journal.pone.0241819)
Supplement: S1 Table — (DOCX) [file pone.0241819.s001.docx]

**S1 Table. Listed of identified proteins from the defined microbial starter in the fermented rice**

| **Accession** | **Description** | **Organism** |
| --- | --- | --- |
| P31334 | 54S ribosomal protein L9, mitochondrial (Mitochondrial large ribosomal subunit protein uL3m) (YmL9) | *Saccharomyces cerevisiae* |
| P32559 | tRNA modification GTPase MSS1, mitochondrial | *Saccharomyces cerevisiae* |
| P08964 | Myosin-1 (Type II myosin) | *Saccharomyces cerevisiae* |
| P15891 | Actin-binding protein | *Saccharomyces cerevisiae* |
| P18544 | Acetylornithine aminotransferase, mitochondrial (ACOAT) (EC 2.6.1.11) | *Saccharomyces cerevisiae* |
| P29366 | Bud emergence protein 1 (Suppressor of RHO3 protein 1) | *Saccharomyces cerevisiae* |
| P07245 | C-1-tetrahydrofolate synthase, cytoplasmic (C1-THF synthase) | *Saccharomyces cerevisiae* |
| P03965 | Carbamoyl-phosphate synthase arginine-specific large chain (EC 6.3.5.5) | *Saccharomyces cerevisiae* |
| P07252 | Cytochrome B pre-mRNA-processing protein 1 | *Saccharomyces cerevisiae* |
| P14905 | Cytochrome B translational activator protein CBS2 | *Saccharomyces cerevisiae* |
| P16522 | Anaphase-promoting complex subunit CDC23 | *Saccharomyces cerevisiae* |
| P00546 | Cyclin-dependent kinase 1 (CDK1) (EC 2.7.11.22) | *Saccharomyces cerevisiae* |
| P08679 | Citrate synthase (EC 2.3.3.16) | *Saccharomyces cerevisiae* |
| P07143 | Cytochrome c1, heme protein, mitochondrial (Complex III subunit 4) (Complex III subunit IV) (Cytochrome b-c1 complex subunit 4) (Ubiquinol-cytochrome-c reductase complex cytochrome c1 subunit) (Cytochrome c-1) | *Saccharomyces cerevisiae* |
| P24783 | ATP-dependent RNA helicase DBP2 (EC 3.6.4.13) | *Saccharomyces cerevisiae* |
| P22281 | Aldehyde dehydrogenase 1, mitochondrial (EC 1.2.1.3) | *Saccharomyces cerevisiae* |
| P02994 | Elongation factor 1-alpha (EF-1-alpha) | *Saccharomyces cerevisiae* |
| P00925 | Enolase 2 (EC 4.2.1.11) | *Saccharomyces cerevisiae* |
| P08524 | Farnesyl pyrophosphate synthase (FPP synthase) (FPS) (EC 2.5.1.10) ((2E,6E)-farnesyl diphosphate synthase) (Dimethylallyltranstransferase) (EC 2.5.1.1) (Farnesyl diphosphate synthase) (Geranyltranstransferase) | *Saccharomyces cerevisiae* |
| P12683 | 3-hydroxy-3-methylglutaryl-coenzyme A reductase 1 (EC 1.1.1.34) | *Saccharomyces cerevisiae* |
| P28241 | Isocitrate dehydrogenase [NAD] subunit 2, mitochondrial (EC 1.1.1.41) | *Saccharomyces cerevisiae* |
| P17119 | Kinesin-like protein KAR3 (Nuclear fusion protein) | *Saccharomyces cerevisiae* |
| P15790 | Casein kinase II subunit alpha (CK II subunit alpha) (EC 2.7.11.1) | *Saccharomyces cerevisiae* |
| P19454 | Casein kinase II subunit alpha' (CK II) (EC 2.7.11.1) | *Saccharomyces cerevisiae* |
| P28743 | Kinesin-like protein KIP2 | *Saccharomyces cerevisiae* |
| P00549 | Pyruvate kinase 1 (PK 1) (EC 2.7.1.40) | *Saccharomyces cerevisiae* |
| P06208 | 2-isopropylmalate synthase (EC 2.3.3.13) | *Saccharomyces cerevisiae* |
| P08638 | Regulatory protein LEU3 | *Saccharomyces cerevisiae* |
| P07702 | L-2-aminoadipate reductase (EC 1.2.1.31) | *Saccharomyces cerevisiae* |
| P21965 | Protein kinase MCK1 (EC 2.7.12.1) (Meiosis and centromere regulatory kinase) | *Saccharomyces cerevisiae* |
| P24279 | DNA replication licensing factor MCM3 (EC 3.6.4.12) | *Saccharomyces cerevisiae* |
| P23500 | Mitochondrial RNA-splicing protein MRS4 | *Saccharomyces cerevisiae* |
| P20676 | Nucleoporin NUP1 (Nuclear pore protein NUP1) | *Saccharomyces cerevisiae* |
| P00560 | Phosphoglycerate kinase (EC 2.7.2.3) | *Saccharomyces cerevisiae* |
| P19735 | Pre-mRNA-splicing factor 6 | *Saccharomyces cerevisiae* |
| P06777 | DNA repair protein RAD1 | *Saccharomyces cerevisiae* |
| P22336 | Replication factor A protein 1 (RF-A protein 1) | *Saccharomyces cerevisiae* |
| P14120 | 60S ribosomal protein L30 (L32) (Large ribosomal subunit protein eL30) (RP73) (YL38) | *Saccharomyces cerevisiae* |
| P06844 | Protein SPT3 (Positive regulator of Ty transcription) | *Saccharomyces cerevisiae* |
| P13574 | Protein STE12 | *Saccharomyces cerevisiae* |
| P05453 | Eukaryotic peptide chain release factor GTP-binding subunit | *Saccharomyces cerevisiae* |
| P22217 | Thioredoxin-1 (Thioredoxin I) (TR-I) (Thioredoxin-2) | *Saccharomyces cerevisiae* |
| P15565 | tRNA (guanine(26)-N(2))-dimethyltransferase, mitochondrial (EC 2.1.1.216) | *Saccharomyces cerevisiae* |
| P26370 | Transcriptional activator protein UGA3 | *Saccharomyces cerevisiae* |
| P22543 | Phosphatidylinositol 3-kinase VPS34 | *Saccharomyces cerevisiae* |
| P25555 | Single-strand telomeric DNA-binding protein GBP2 | *Saccharomyces cerevisiae* |
| P25619 | 30 kDa heat shock protein | *Saccharomyces cerevisiae* |
| P07262 | NADP-specific glutamate dehydrogenase 1 (NADP-GDH 1) (EC 1.4.1.4) (NADP-dependent glutamate dehydrogenase 1) | *Saccharomyces cerevisiae* |
| P19262 | Dihydrolipoyllysine-residue succinyltransferase component of 2-oxoglutarate dehydrogenase complex, mitochondrial (EC 2.3.1.61) | *Saccharomyces cerevisiae* |
| P18408 | Phosphoadenosine phosphosulfate reductase (EC 1.8.4.8) | *Saccharomyces cerevisiae* |
| P07275 | Delta-1-pyrroline-5-carboxylate dehydrogenase, mitochondrial (P5C dehydrogenase) (EC 1.2.1.88) (L-glutamate gamma-semialdehyde dehydrogenase) | *Saccharomyces cerevisiae* |
| Q00816 | Resistance to glucose repression protein 1 (Protein HEX2) (Second-site suppressor of the rna1-1 mutation 1) | *Saccharomyces cerevisiae* |
| Q02100 | CRE-binding bZIP protein SKO1 | *Saccharomyces cerevisiae* |
| Q02821 | Importin subunit alpha (Karyopherin subunit alpha) (Karyopherin-60) (Serine-rich RNA polymerase I suppressor protein) | *Saccharomyces cerevisiae* |
| P31378 | Endonuclease III homolog 1 (EC 3.2.2.-) (EC 4.2.99.18) | *Saccharomyces cerevisiae* |
| P11986 | Inositol-3-phosphate synthase (EC 5.5.1.4) | *Saccharomyces cerevisiae* |
| P32794 | ATPase family gene 2 protein (EC 3.6.4.10) | *Saccharomyces cerevisiae* |
| P32448 | Anti-silencing protein 2 | *Saccharomyces cerevisiae* |
| P32558 | FACT complex subunit SPT16 (Cell division control protein 68) | *Saccharomyces cerevisiae* |
| P32799 | Cytochrome c oxidase subunit 6A, mitochondrial (EC 1.9.3.1) | *Saccharomyces cerevisiae* |
| P32800 | Peroxisomal biogenesis factor 2 (Peroxin-2) (Peroxisomal protein PAS5) (Protein CRT1) | *Saccharomyces cerevisiae* |
| P32590 | Heat shock protein homolog SSE2 | *Saccharomyces cerevisiae* |
| P32465 | Low-affinity glucose transporter HXT1 | *Saccharomyces cerevisiae* |
| P32333 | TATA-binding protein-associated factor MOT1 (TBP-associated factor MOT1) (EC 3.6.4.-) | *Saccharomyces cerevisiae* |
| P32494 | Chromatin-remodeling complexes subunit NGG1 (Transcriptional adapter 3) | *Saccharomyces cerevisiae* |
| P32380 | Spindle pole body component 110 (Extragenic suppressor of CMD1-1 mutant protein 1) (Nuclear filament-related protein 1) (Spindle pole body spacer protein SPC110) | *Saccharomyces cerevisiae* |
| P32319 | Vacuolar protein sorting/targeting protein VPS10 (Vacuolar protein-targeting protein 1) | *Saccharomyces cerevisiae* |
| P32383 | 1-phosphatidylinositol 4,5-bisphosphate phosphodiesterase 1 (EC 3.1.4.11) | *Saccharomyces cerevisiae* |
| P32863 | DNA repair and recombination protein RAD54 (EC 3.6.4.-) | *Saccharomyces cerevisiae* |
| P32915 | Protein transport protein SEC61 | *Saccharomyces cerevisiae* |
| P32771 | S-(hydroxymethyl)glutathione dehydrogenase (EC 1.1.1.284) (Alcohol dehydrogenase SFA) (EC 1.1.1.1) | *Saccharomyces cerevisiae* |
| Q01389 | Serine/threonine-protein kinase BCK1/SLK1/SSP31 (EC 2.7.11.1) | *Saccharomyces cerevisiae* |
| P32579 | Threonylcarbamoyl-AMP synthase (TC-AMP synthase) (EC 2.7.7.87) | *Saccharomyces cerevisiae* |
| P32861 | UTP--glucose-1-phosphate uridylyltransferase (EC 2.7.7.9) | *Saccharomyces cerevisiae* |
| P32657 | Chromo domain-containing protein 1 (EC 3.6.4.-) | *Saccharomyces cerevisiae* |
| P32634 | Negative regulator of sporulation PMD1 | *Saccharomyces cerevisiae* |
| P32342 | Signal recognition particle subunit SRP21 (Signal recognition particle 21 kDa protein) | *Saccharomyces cerevisiae* |
| P32899 | U3 small nucleolar ribonucleoprotein protein IMP3 | *Saccharomyces cerevisiae* |
| P32527 | Zuotin (DnaJ-related protein ZUO1) (J protein ZUO1) (Heat shock protein 40 homolog ZUO1) (Ribosome-associated complex subunit ZUO1) | *Saccharomyces cerevisiae* |
| P36089 | Putative uncharacterized protein YKL066W | *Saccharomyces cerevisiae* |
| P32048 | Lysine--tRNA ligase, mitochondrial (EC 6.1.1.6) (Lysyl-tRNA synthetase) (LysRS) | *Saccharomyces cerevisiae* |
| P33892 | eIF-2-alpha kinase activator GCN1 (General control non-derepressible protein 1) (Translational activator GCN1) | *Saccharomyces cerevisiae* |
| P33748 | Zinc finger protein MSN2 (Multicopy suppressor of SNF1 protein 2) | *Saccharomyces cerevisiae* |
| P35182 | Protein phosphatase 2C homolog 1 (PP2C-1) (EC 3.1.3.16) | *Saccharomyces cerevisiae* |
| P33334 | Pre-mRNA-splicing factor 8 | *Saccharomyces cerevisiae* |
| P33332 | Exocyst complex component SEC3 (Protein PSL1) | *Saccharomyces cerevisiae* |
| P33442 | 40S ribosomal protein S1-A (RP10A) (Small ribosomal subunit protein eS1-A) | *Saccharomyces cerevisiae* |
| P33300 | Mannosyl phosphorylinositol ceramide synthase SUR1 (EC 2.4.-.-) | *Saccharomyces cerevisiae* |
| P32571 | Ubiquitin carboxyl-terminal hydrolase 4 (EC 3.4.19.12) | *Saccharomyces cerevisiae* |
| P33767 | Dolichyl-diphosphooligosaccharide--protein glycosyltransferase subunit WBP1 (Oligosaccharyl transferase subunit WBP1) (Oligosaccharyl transferase subunit beta) | *Saccharomyces cerevisiae* |
| P35195 | UPF0045 protein ECM15 (Extracellular mutant protein 15) | *Saccharomyces cerevisiae* |
| P34233 | Transcriptional regulatory protein ASH1 (Daughter cells HO repressor protein) | *Saccharomyces cerevisiae* |
| P35688 | Rho-GTPase-activating protein LRG1 (LIM-RhoGAP protein 1) | *Saccharomyces cerevisiae* |
| A2P1U8 | ORF | *Saccharomyces cerevisiae* |
| P06738 | Glycogen phosphorylase (EC 2.4.1.1) | *Saccharomyces cerevisiae* |
| P35843 | Protein HES1 (Oxysterol-binding protein homolog 5) | *Saccharomyces cerevisiae* |
| P36010 | Nucleoside diphosphate kinase (NDK) (NDP kinase) (EC 2.7.4.6) | *Saccharomyces cerevisiae* |
| P36421 | Tyrosine--tRNA ligase, cytoplasmic (EC 6.1.1.1) (Tyrosyl-tRNA synthetase) | *Saccharomyces cerevisiae* |
| P23501 | Dihydrosphingosine 1-phosphate phosphatase YSR3 (EC 3.1.3.-) | *Saccharomyces cerevisiae* |
| P36144 | Ribosome biogenesis protein UTP30 (U3 snoRNP-associated protein UTP30) | *Saccharomyces cerevisiae* |
| P36023 | Oleate activated transcription factor 3 | *Saccharomyces cerevisiae* |
| P36160 | Ribosome biogenesis protein RPF2 | *Saccharomyces cerevisiae* |
| P36099 | Putative uncharacterized protein YKL030W | *Saccharomyces cerevisiae* |
| P36069 | Probable phosphoglycerate mutase PMU1 (EC 5.4.-.-) (Phosphomutase homolog 1) | *Saccharomyces cerevisiae* |
| P36056 | Probable S-adenosyl-L-methionine-dependent RNA methyltransferase RSM22, mitochondrial (EC 2.1.1.-) | *Saccharomyces cerevisiae* |
| P36051 | GPI ethanolamine phosphate transferase 1 | *Saccharomyces cerevisiae* |
| P28273 | 5-oxoprolinase (EC 3.5.2.9) | *Saccharomyces cerevisiae* |
| P38113 | Alcohol dehydrogenase 5 (EC 1.1.1.1) | *Saccharomyces cerevisiae* |
| P38157 | Maltose fermentation regulatory protein MAL33 | *Saccharomyces cerevisiae* |
| P38622 | Serine/threonine-protein kinase RCK1 (EC 2.7.11.1) | *Saccharomyces cerevisiae* |
| P37297 | Phosphatidylinositol 4-kinase STT4 (PI4-kinase) (PtdIns-4-kinase) (EC 2.7.1.67) | *Saccharomyces cerevisiae* |
| P38316 | Ubiquitin-like protein ATG12 (Autophagy-related protein 12) | *Saccharomyces cerevisiae* |
| P38323 | ATP-dependent clpX-like chaperone, mitochondrial (mtClpX) (ATP-dependent unfoldase ClpX) | *Saccharomyces cerevisiae* |
| P38330 | Protein RMD9-like, mitochondrial | *Saccharomyces cerevisiae* |
| P38361 | Phosphate permease PHO89 (Na(+)/Pi cotransporter PHO89) | *Saccharomyces cerevisiae* |
| P38340 | Alpha N-terminal protein methyltransferase 1 (EC 2.1.1.244) | *Saccharomyces cerevisiae* |
| P38202 | UPF0642 protein YBL028C | *Saccharomyces cerevisiae* |
| P38175 | 37S ribosomal protein MRP21, mitochondrial | *Saccharomyces cerevisiae* |
| P38124 | Fluconazole resistance protein 1 | *Saccharomyces cerevisiae* |
| P38226 | Uncharacterized acyltransferase CST26 (EC 2.3.-.-) (Chromosome stability protein 26) | *Saccharomyces cerevisiae* |
| P38085 | Valine/tyrosine/tryptophan amino-acid permease 1 (Tyrosine and tryptophan amino acid transporter 1) | *Saccharomyces cerevisiae* |
| P38264 | SRP-independent targeting protein 3 (Inorganic phosphate transport protein PHO88) | *Saccharomyces cerevisiae* |
| P39710 | Putative uncharacterized protein YAL066W | *Saccharomyces cerevisiae* |
| P38720 | 6-phosphogluconate dehydrogenase, decarboxylating 1 (EC 1.1.1.44) | *Saccharomyces cerevisiae* |
| P39969 | Protein BOI2 (Protein BEB1) | *Saccharomyces cerevisiae* |
| P39101 | Protein CAJ1 | *Saccharomyces cerevisiae* |
| P04817 | Arginine permease CAN1 (Canavanine resistance protein 1) | *Saccharomyces cerevisiae* |
| P38991 | Spindle assembly checkpoint kinase (EC 2.7.11.1) | *Saccharomyces cerevisiae* |
| P38692 | Serine/threonine-protein kinase KIC1 (EC 2.7.11.1) | *Saccharomyces cerevisiae* |
| P38689 | Ribose-phosphate pyrophosphokinase 3 (EC 2.7.6.1) | *Saccharomyces cerevisiae* |
| P39518 | Long-chain-fatty-acid--CoA ligase 2 (EC 6.2.1.3) (Fatty acid activator 2) (Long-chain acyl-CoA synthetase 2) | *Saccharomyces cerevisiae* |
| P40958 | Mitotic spindle checkpoint component MAD2 (Mitotic MAD2 protein) | *Saccharomyces cerevisiae* |
| P07266 | Mitochondrial RNA-splicing protein MRS1 | *Saccharomyces cerevisiae* |
| Q00539 | Protein NAM8 | *Saccharomyces cerevisiae* |
| P25560 | Protein RER1 (Retention of ER proteins 1) | *Saccharomyces cerevisiae* |
| P40348 | Replication factor C subunit 2 (Replication factor C2) | *Saccharomyces cerevisiae* |
| P21672 | Ribonucleoside-diphosphate reductase large chain 2 (EC 1.17.4.1) | *Saccharomyces cerevisiae* |
| P32844 | Exocyst complex component SEC6 | *Saccharomyces cerevisiae* |
| P39929 | Vacuolar-sorting protein SNF7 (DOA4-independent degradation protein 1) | *Saccharomyces cerevisiae* |
| P25302 | Regulatory protein SWI4 (Cell-cycle box factor subunit SWI4) (Protein ART1) | *Saccharomyces cerevisiae* |
| P39944 | Ubiquitin carboxyl-terminal hydrolase 5 (EC 3.4.19.12) | *Saccharomyces cerevisiae* |
| P39722 | Mitochondrial Rho GTPase 1 (EC 3.6.5.-) (GTPase EF-hand protein of mitochondria 1) | *Saccharomyces cerevisiae* |
| P40008 | Protein FMP52, mitochondrial (Found in mitochondrial proteome protein 52) | *Saccharomyces cerevisiae* |
| P40009 | Golgi apyrase (EC 3.6.1.5) | *Saccharomyces cerevisiae* |
| P40024 | ABC transporter ATP-binding protein ARB1 (ATP-binding cassette protein involved in ribosome biogenesis 1) | *Saccharomyces cerevisiae* |
| P40046 | Vacuolar transporter chaperone 1 | *Saccharomyces cerevisiae* |
| P40053 | Altered inheritance of mitochondria protein 9, mitochondrial (Found in mitochondrial proteome protein 29) | *Saccharomyces cerevisiae* |
| P40074 | Vacuolar amino acid transporter 6 | *Saccharomyces cerevisiae* |
| P38750 | Uncharacterized transporter YHL008C | *Saccharomyces cerevisiae* |
| P38741 | Meiotic activator RIM4 (Regulator of IME2 protein 4) | *Saccharomyces cerevisiae* |
| P38734 | Low-affinity methionine permease | *Saccharomyces cerevisiae* |
| P38726 | Putative UPF0377 protein YHL045W | *Saccharomyces cerevisiae* |
| P38756 | tRNA threonylcarbamoyladenosine dehydratase 1 (EC 6.1.-.-) (t(6)A37 dehydratase 1) | *Saccharomyces cerevisiae* |
| P38760 | RNA-binding protein MIP6 (MEX67-interacting protein 6) | *Saccharomyces cerevisiae* |
| P38791 | Deoxyhypusine synthase (DHS) (EC 2.5.1.46) | *Saccharomyces cerevisiae* |
| P38713 | Oxysterol-binding protein homolog 3 | *Saccharomyces cerevisiae* |
| P38795 | Glutamine-dependent NAD(+) synthetase (EC 6.3.5.1) | *Saccharomyces cerevisiae* |
| P38816 | Thioredoxin reductase 2, mitochondrial (EC 1.8.1.9) | *Saccharomyces cerevisiae* |
| P38820 | Adenylyltransferase and sulfurtransferase UBA4 | *Saccharomyces cerevisiae* |
| P38821 | Aspartyl aminopeptidase 4 (EC 3.4.11.21) | *Saccharomyces cerevisiae* |
| P38859 | DNA replication ATP-dependent helicase/nuclease DNA2 [Includes: DNA replication nuclease DNA2 (EC 3.1.-.-); DNA replication ATP-dependent helicase DNA2 (EC 3.6.4.12)] | *Saccharomyces cerevisiae* |
| P38699 | Protein STB5 | *Saccharomyces cerevisiae* |
| P38879 | Nascent polypeptide-associated complex subunit alpha (NAC-alpha) (Alpha-NAC) (GAL4 DNA-binding enhancer protein 2) | *Saccharomyces cerevisiae* |
| P38885 | Altered inheritance of mitochondria protein 46, mitochondrial | *Saccharomyces cerevisiae* |
| P40556 | Mitochondrial nicotinamide adenine dinucleotide transporter 1 (Mitochondrial NAD(+) transporter 1) | *Saccharomyces cerevisiae* |
| P40540 | ER membrane protein complex subunit 5 (Killer toxin-resistance protein 27) | *Saccharomyces cerevisiae* |
| P40527 | Probable phospholipid-transporting ATPase NEO1 (EC 7.6.2.1) | *Saccharomyces cerevisiae* |
| P40188 | Respiratory growth induced protein 2 | *Saccharomyces cerevisiae* |
| P40517 | Ran-specific GTPase-activating protein 2 (Ran-binding protein 2) (RANBP2) | *Saccharomyces cerevisiae* |
| P40505 | Transcriptional regulatory protein SDS3 (Suppressor of defective silencing protein 3) | *Saccharomyces cerevisiae* |
| P40490 | Putative uncharacterized protein YIL100W | *Saccharomyces cerevisiae* |
| P40486 | Protein SHQ1 (Small nucleolar RNAs of the box H/ACA family quantitative accumulation protein 1) | *Saccharomyces cerevisiae* |
| P40480 | Protein HOS4 | *Saccharomyces cerevisiae* |
| P40562 | ATP-dependent DNA helicase MPH1 (EC 3.6.4.12) | *Saccharomyces cerevisiae* |
| P39535 | Low-affinity phosphate transporter PHO90 | *Saccharomyces cerevisiae* |
| P40892 | Putative acetyltransferase YJL218W (EC 2.3.1.-) | *Saccharomyces cerevisiae* |
| P40345 | Phospholipid:diacylglycerol acyltransferase (PDAT) (EC 2.3.1.158) | *Saccharomyces cerevisiae* |
| Q7GID8 | Uncharacterized protein | *Saccharomyces cerevisiae* |
| Q04434 | Putative uncharacterized protein YDR535C | *Saccharomyces cerevisiae* |
| E9PA68 | J0919 protein | *Saccharomyces cerevisiae* |
| E9PA69 | J0925 protein | *Saccharomyces cerevisiae* |
| V9H0W3 | L3118 protein | *Saccharomyces cerevisiae* |
| E9PAA2 | L3151 protein | *Saccharomyces cerevisiae* |
| P53549 | 26S proteasome subunit RPT4 (26S protease subunit SUG2) (Proteasomal cap subunit) | *Saccharomyces cerevisiae* |
| P41697 | Bud site selection protein 6 (Actin-interacting protein 3) | *Saccharomyces cerevisiae* |
| P43550 | Dihydroxyacetone kinase 2 (DHA kinase 2) (EC 2.7.1.28) (EC 2.7.1.29) (Glycerone kinase 2) (Triokinase 2) (Triose kinase 2) | *Saccharomyces cerevisiae* |
| P41819 | Dimethyladenosine transferase (EC 2.1.1.183) (18S rRNA (adenine(1779)-N(6)/adenine(1780)-N(6))-dimethyltransferase) (18S rRNA dimethylase) (S-adenosylmethionine-6-N', N'-adenosyl(rRNA) dimethyltransferase) | *Saccharomyces cerevisiae* |
| P42940 | Probable electron transfer flavoprotein subunit beta (Beta-ETF) (Changed intracellular redox state protein 1) | *Saccharomyces cerevisiae* |
| P38911 | FK506-binding nuclear protein (EC 5.2.1.8) (FKBP-70) (Nucleolar proline isomerase) (Peptidyl-prolyl cis-trans isomerase) (PPIase) (Proline rotamase) | *Saccharomyces cerevisiae* |
| P41814 | tRNA (adenine(58)-N(1))-methyltransferase non-catalytic subunit TRM6 (General control non-derepressible protein 10) (Protein GCD10) (tRNA(m1A58)-methyltransferase subunit TRM6) (tRNA(m1A58)MTase subunit TRM6) | *Saccharomyces cerevisiae* |
| P43639 | Casein kinase II subunit beta (CK II beta) | *Saccharomyces cerevisiae* |
| P43588 | Ubiquitin carboxyl-terminal hydrolase RPN11 (EC 3.4.19.12) (26S proteasome regulatory subunit RPN11) (Protein MPR1) | *Saccharomyces cerevisiae* |
| P46964 | Dolichyl-diphosphooligosaccharide--protein glycosyltransferase subunit OST2 | *Saccharomyces cerevisiae* |
| P46971 | Dolichyl-phosphate-mannose--protein mannosyltransferase 4 (EC 2.4.1.109) | *Saccharomyces cerevisiae* |
| P41805 | 60S ribosomal protein L10 (L9) (Large ribosomal subunit protein uL16) (Ubiquinol-cytochrome C reductase complex subunit VI-requiring protein) | *Saccharomyces cerevisiae* |
| P06105 | Protein SCP160 (Protein HX) | *Saccharomyces cerevisiae* |
| P43472 | Sucrose operon repressor | *Pediococcus pentosaceus* |
| P41833 | N6-adenosine-methyltransferase IME4 (EC 2.1.1.348) | *Saccharomyces cerevisiae* |
| P46677 | Transcription initiation factor TFIID subunit 1 (EC 2.3.1.48) | *Saccharomyces cerevisiae* |
| P42943 | T-complex protein 1 subunit eta (TCP-1-eta) (CCT-eta) | *Saccharomyces cerevisiae* |
| P35169 | Serine/threonine-protein kinase TOR1 (EC 2.7.11.1) | *Saccharomyces cerevisiae* |
| P43569 | CCR4-associated factor 16 | *Saccharomyces cerevisiae* |
| P43605 | N-acetyltransferase ECO1 (EC 2.3.1.-) (Chromosome transmission fidelity protein 7) (Establishment of cohesion protein 1) | *Saccharomyces cerevisiae* |
| P43609 | Chromatin structure-remodeling complex protein RSC8 | *Saccharomyces cerevisiae* |
| P46949 | Protein FYV8 (Function required for yeast viability protein 8) | *Saccharomyces cerevisiae* |
| E9PAF7 | N1890 protein | *Saccharomyces cerevisiae* |
| Q12130 | Uncharacterized protein YLR112W | *Saccharomyces cerevisiae* |
| P11433 | Cell division control protein 24 (Calcium regulatory protein) | *Saccharomyces cerevisiae* |
| P49095 | Glycine dehydrogenase (decarboxylating), mitochondrial (EC 1.4.4.2) | *Saccharomyces cerevisiae* |
| P25045 | Serine palmitoyltransferase 1 (SPT 1) (SPT1) (EC 2.3.1.50) | *Saccharomyces cerevisiae* |
| P04046 | Amidophosphoribosyltransferase (ATase) (EC 2.4.2.14) | *Saccharomyces cerevisiae* |
| P48581 | DNA damage checkpoint control protein RAD17 (DNA repair exonuclease RAD17) | *Saccharomyces cerevisiae* |
| P47821 | RNA polymerase II holoenzyme cyclin-like subunit (Suppressor of RNA polymerase B 11) | *Saccharomyces cerevisiae* |
| P49090 | Asparagine synthetase [glutamine-hydrolyzing] 2 (EC 6.3.5.4) | *Saccharomyces cerevisiae* |
| P48439 | Dolichyl-diphosphooligosaccharide--protein glycosyltransferase subunit 3 | *Saccharomyces cerevisiae* |
| P47124 | Putative glycosyltransferase HOC1 (EC 2.4.-.-) (M-Pol II subunit Hoc1p) (Mannan polymerase II complex HOC1 subunit) | *Saccharomyces cerevisiae* |
| P47130 | Cop9 signalosome complex subunit 12 | *Saccharomyces cerevisiae* |
| P47133 | ER membrane protein complex subunit 2 | *Saccharomyces cerevisiae* |
| P47160 | Epsin-3 | *Saccharomyces cerevisiae* |
| P47161 | Vacuolar protein sorting-associated protein 70 (EC 3.4.-.-) | *Saccharomyces cerevisiae* |
| P47077 | Nucleolar protein 9 (Pumilio domain-containing protein NOP9) | *Saccharomyces cerevisiae* |
| P47045 | Mitochondrial import inner membrane translocase subunit TIM54 | *Saccharomyces cerevisiae* |
| P47090 | Uncharacterized endoplasmic reticulum membrane protein YJR015W | *Saccharomyces cerevisiae* |
| P48836 | V-type proton ATPase subunit G (V-ATPase subunit G) (V-ATPase 13 kDa subunit) (Vacuolar proton pump subunit G) | *Saccharomyces cerevisiae* |
| P48561 | Poly(A) RNA polymerase protein 1 (EC 2.7.7.19) (Topoisomerase 1-related protein TRF5) | *Saccharomyces cerevisiae* |
| E9PAB4 | Uncharacterized protein | *Saccharomyces cerevisiae* |
| P53909 | Adenine deaminase (ADE) (EC 3.5.4.2) (Adenine aminohydrolase) (AAH) | *Saccharomyces cerevisiae* |
| P32451 | Biotin synthase, mitochondrial (EC 2.8.1.6) | *Saccharomyces cerevisiae* |
| P41695 | Checkpoint serine/threonine-protein kinase BUB1 (EC 2.7.11.1) | *Saccharomyces cerevisiae* |
| P33307 | Importin alpha re-exporter (Chromosome segregation protein CSE1) | *Saccharomyces cerevisiae* |
| P54838 | Dihydroxyacetone kinase 1 (DHA kinase 1) (EC 2.7.1.28) (EC 2.7.1.29) | *Saccharomyces cerevisiae* |
| P15801 | DNA polymerase gamma (EC 2.7.7.7) | *Saccharomyces cerevisiae* |
| Q03018 | Separin (EC 3.4.22.49) (Separase) | *Saccharomyces cerevisiae* |
| P53551 | Histone H1 | *Saccharomyces cerevisiae* |
| P54862 | Hexose transporter HXT11 (Low-affinity glucose transporter LGT3) | *Saccharomyces cerevisiae* |
| P54854 | Hexose transporter HXT15 | *Saccharomyces cerevisiae* |
| P53341 | Alpha-glucosidase MAL12 (EC 3.2.1.20) (Maltase) | *Saccharomyces cerevisiae* |
| P53583 | Protein MPA43 | *Saccharomyces cerevisiae* |
| P50105 | Transcription initiation factor TFIID subunit 4 (MPT-1) (TAF suppressor gene 2 protein) (TAFII-48) (TBP-associated factor 4) (TBP-associated factor 48 kDa) | *Saccharomyces cerevisiae* |
| P52920 | Cytosolic Fe-S cluster assembly factor NBP35 (Nucleotide-binding protein 35) | *Saccharomyces cerevisiae* |
| P39946 | Nuclear distribution protein PAC1 (Lissencephaly-1 homolog) (LIS-1) (nudF homolog) | *Saccharomyces cerevisiae* |
| P51533 | ATP-dependent permease PDR10 | *Saccharomyces cerevisiae* |
| P32854 | Syntaxin PEP12 (Carboxypeptidase Y-deficient protein 12) (Vacuolar protein sorting-associated protein 6) (Vacuolar protein-targeting protein 13) | *Saccharomyces cerevisiae* |
| P39008 | Poly(A) ribonuclease POP2 (EC 3.1.13.4) (CCR4-associated factor 1) | *Saccharomyces cerevisiae* |
| P38972 | Phosphoribosylformylglycinamidine synthase (FGAM synthase) (FGAMS) (EC 6.3.5.3) | *Saccharomyces cerevisiae* |
| P51401 | 60S ribosomal protein L9-B (L8) | *Saccharomyces cerevisiae* |
| P22224 | Exocyst complex component SEC15 | *Saccharomyces cerevisiae* |
| P53012 | FIT family protein SCS3 | *Saccharomyces cerevisiae* |
| P08459 | Sporulation-specific protein 2 | *Saccharomyces cerevisiae* |
| P46679 | Protein STB2 | *Saccharomyces cerevisiae* |
| P37296 | V-type proton ATPase subunit a, Golgi isoform (V-ATPase a 2 subunit) | *Saccharomyces cerevisiae* |
| P53394 | Putative sulfate transporter YPR003C | *Saccharomyces cerevisiae* |
| P07284 | Serine--tRNA ligase, cytoplasmic (EC 6.1.1.11) (Seryl-tRNA synthetase) | *Saccharomyces cerevisiae* |
| P32356 | Neutral trehalase (EC 3.2.1.28) (Alpha,alpha-trehalase) | *Saccharomyces cerevisiae* |
| P32366 | V-type proton ATPase subunit d (V-ATPase subunit d) | *Saccharomyces cerevisiae* |
| P39731 | Kinetochore-associated protein MTW1 (Mis12-like protein) | *Saccharomyces cerevisiae* |
| P53244 | Arrestin-related trafficking adapter 5 | *Saccharomyces cerevisiae* |
| P53250 | Twinfilin-1 (Twinfilin-A) | *Saccharomyces cerevisiae* |
| P53252 | Sphingolipid long chain base-responsive protein PIL1 | *Saccharomyces cerevisiae* |
| P53280 | Protein CAF130 (130 kDa CCR4-associated factor) | *Saccharomyces cerevisiae* |
| P53294 | tRNA pseudouridine(31) synthase (EC 5.4.99.42) | *Saccharomyces cerevisiae* |
| P53298 | Inner kinetochore subunit OKP1 (CENP-Q homolog) (Constitutive centromere-associated network protein OKP1) ) | *Saccharomyces cerevisiae* |
| P50077 | Calcium-channel protein CCH1 | *Saccharomyces cerevisiae* |
| P53334 | Probable family 17 glucosidase SCW4 (EC 3.2.1.-) | *Saccharomyces cerevisiae* |
| P53166 | ATP-dependent RNA helicase MRH4, mitochondrial (EC 3.6.4.13) | *Saccharomyces cerevisiae* |
| P53144 | HD domain-containing protein YGL101W | *Saccharomyces cerevisiae* |
| P53119 | Probable E3 ubiquitin-protein ligase HUL5 (EC 2.3.2.26) | *Saccharomyces cerevisiae* |
| P53086 | Kinesin-like protein KIP3 | *Saccharomyces cerevisiae* |
| P53073 | ER membrane protein complex subunit 4 | *Saccharomyces cerevisiae* |
| P40340 | Tat-binding homolog 7 | *Saccharomyces cerevisiae* |
| P50273 | Mitochondrial translation factor ATP22 | *Saccharomyces cerevisiae* |
| P47988 | TY1 enhancer activator | *Saccharomyces cerevisiae* |
| P53894 | Serine/threonine-protein kinase CBK1 (EC 2.7.11.1) | *Saccharomyces cerevisiae* |
| P55250 | Fumarate hydratase, mitochondrial (Fumarase) (EC 4.2.1.2) | *Rhizopus oryzae* |
| P17442 | Phosphate system positive regulatory protein PHO81 (CDK inhibitor PHO81) | *Saccharomyces cerevisiae* |
| P49954 | Omega-amidase NIT3 (EC 3.5.1.3) (Nitrilase homolog 2) | *Saccharomyces cerevisiae* |
| P40308 | Lipase 3 (EC 3.1.1.3) (Triacylglycerol lipase 3) | *Saccharomyces cerevisiae* |
| P54837 | Endoplasmic reticulum vesicle protein 25 | *Saccharomyces cerevisiae* |
| P53830 | Cold sensitive U2 snRNA suppressor 2 | *Saccharomyces cerevisiae* |
| P53823 | Probable pyridoxal 5'-phosphate synthase subunit SNO2 (EC 4.3.3.6) (PDX2 homolog 2) (Pdx2.2) (Pyridoxal 5'-phosphate synthase glutaminase subunit) (EC 3.5.1.2) | *Saccharomyces cerevisiae* |
| P53723 | UPF0674 endoplasmic reticulum membrane protein YNR021W | *Saccharomyces cerevisiae* |
| P53725 | M-phase phosphoprotein 6 homolog (Exosome-associated RNA-binding protein MPP6) | *Saccharomyces cerevisiae* |
| P53741 | UBP3-associated protein BRE5 (Brefeldin-A sensitivity protein 5) | *Saccharomyces cerevisiae* |
| P53743 | Pre-rRNA-processing protein ESF2 (18S rRNA factor 2) | *Saccharomyces cerevisiae* |
| P53953 | SED5-binding protein 2 (SEC24-related protein 2) | *Saccharomyces cerevisiae* |
| P53950 | Vacuolar segregation protein 7 | *Saccharomyces cerevisiae* |
| P53944 | Mitochondrial MRF1 N(5)-glutamine methyltransferase MTQ1 (EC 2.1.1.297) | *Saccharomyces cerevisiae* |
| P50947 | Transcriptional regulatory protein PHO23 | *Saccharomyces cerevisiae* |
| P50942 | Polyphosphatidylinositol phosphatase INP52 (Synaptojanin-like protein 2) | *Saccharomyces cerevisiae* |
| P53893 | Ribosome assembly protein 1 (EC 3.6.5.-) (EF-2-like GTPase) (Elongation factor-like 1) | *Saccharomyces cerevisiae* |
| P54964 | Oligoribonuclease, mitochondrial (EC 3.1.-.-) | *Saccharomyces cerevisiae* |
| P53855 | Autophagy-related protein 2 (Sporulation-specific protein 72) | *Saccharomyces cerevisiae* |
| P51996 | GTP-binding protein YPT32/YPT11 (Rab GTPase YPT32) | *Saccharomyces cerevisiae* |
| P31111 | Synaptonemal complex protein ZIP1 | *Saccharomyces cerevisiae* |
| P87271 | Putative uncharacterized protein YDR455C | *Saccharomyces cerevisiae* |
| O13557 | Putative uncharacterized protein YLR463C | *Saccharomyces cerevisiae* |
| O13575 | Putative uncharacterized protein YPR177C | *Saccharomyces cerevisiae* |
| Q12386 | Actin-like protein ARP8 | *Saccharomyces cerevisiae* |
| Q12675 | Phospholipid-transporting ATPase DNF2 (EC 7.6.2.1) ( | *Saccharomyces cerevisiae* |
| Q12674 | Probable phospholipid-transporting ATPase DNF3 (EC 7.6.2.1) | *Saccharomyces cerevisiae* |
| Q04487 | Mitochondrial inner membrane protein SHH3 (SDH3 homolog) | *Saccharomyces cerevisiae* |
| Q08271 | 1,3-beta-glucanosyltransferase GAS4 (EC 2.4.1.-) (Glycolipid-anchored surface protein 4) | *Saccharomyces cerevisiae* |
| Q12001 | Dolichyl pyrophosphate Man9GlcNAc2 alpha-1,3-glucosyltransferase (EC 2.4.1.267) | *Saccharomyces cerevisiae* |
| Q04651 | ER-derived vesicles protein ERV41 | *Saccharomyces cerevisiae* |
| Q04371 | Damage-control phosphatase YMR027W (EC 3.1.3.-) (Sugar phosphate phosphatase YMR027W) | *Saccharomyces cerevisiae* |
| Q04264 | Sister chromatid cohesion protein PDS5 | *Saccharomyces cerevisiae* |
| Q04305 | U3 small nucleolar RNA-associated protein 15 (U3 snoRNA-associated protein 15) | *Saccharomyces cerevisiae* |
| Q04471 | Abasic site processing protein YMR114C | *Saccharomyces cerevisiae* |
| Q04216 | Defect at low temperature protein 1 | *Saccharomyces cerevisiae* |
| Q03790 | Nucleoporin NUP53 (Nuclear pore protein NUP53) | *Saccharomyces cerevisiae* |
| Q03694 | Inheritance of peroxisomes protein 1 | *Saccharomyces cerevisiae* |
| Q04991 | Protein FMP42 | *Saccharomyces cerevisiae* |
| Q05024 | Protein TRI1 | *Saccharomyces cerevisiae* |
| Q12060 | Transcriptional coactivator HFI1/ADA1 | *Saccharomyces cerevisiae* |
| Q99189 | mRNA transport regulator MTR10 | *Saccharomyces cerevisiae* |
| Q02820 | Non-classical export protein 1 | *Saccharomyces cerevisiae* |
| Q08108 | Lysophospholipase 3 (EC 3.1.1.5) (Phospholipase B 3) | *Saccharomyces cerevisiae* |
| Q99176 | Protein SRN2 (ESCRT-I complex subunit VPS37) (Vacuolar protein sorting-associated protein 37) | *Saccharomyces cerevisiae* |
| Q12326 | Phosphoglycerate mutase 3 (PGAM 3) (EC 5.4.2.11) | *Saccharomyces cerevisiae* |
| Q12469 | Serine/threonine-protein kinase SKM1 (EC 2.7.11.1) | *Saccharomyces cerevisiae* |
| Q12099 | ATP-dependent RNA helicase FAL1 (EC 3.6.4.13) | *Saccharomyces cerevisiae* |
| Q07478 | ATP-dependent RNA helicase SUB2 (EC 3.6.4.13) | *Saccharomyces cerevisiae* |
| Q03532 | ATP-dependent RNA helicase HAS1 (EC 3.6.4.13) | *Saccharomyces cerevisiae* |
| Q99383 | Nuclear polyadenylated RNA-binding protein 4 | *Saccharomyces cerevisiae* |
| Q12404 | Protein disulfide-isomerase MPD1 (EC 5.3.4.1) | *Saccharomyces cerevisiae* |
| Q02891 | Medium-chain fatty acid ethyl ester synthase/esterase 1 (Alcohol O-acetyltransferase) (EC 2.3.1.84) (EC 3.1.1.-) (Ethyl ester biosynthesis protein 1) | *Saccharomyces cerevisiae* |
| Q06639 | Chromatin structure-remodeling complex protein RSC3 | *Saccharomyces cerevisiae* |
| P14772 | Bile pigment transporter 1 | *Saccharomyces cerevisiae* |
| P09032 | Translation initiation factor eIF-2B subunit gamma | *Saccharomyces cerevisiae* |
| P53622 | Coatomer subunit alpha (Alpha-coat protein) | *Saccharomyces cerevisiae* |
| P22007 | Protein farnesyltransferase subunit beta (FTase-beta) (EC 2.5.1.58) | *Saccharomyces cerevisiae* |
| P25847 | DNA mismatch repair protein MSH2 (MutS protein homolog 2) | *Saccharomyces cerevisiae* |
| P38959 | Vacuolar protein sorting-associated protein 41 (Vacuolar morphogenesis protein 2) | *Saccharomyces cerevisiae* |
| P22023 | Killer toxin-resistance protein 5 | *Saccharomyces cerevisiae* |
| P32336 | Protein NUD1 | *Saccharomyces cerevisiae* |
| P06776 | 3',5'-cyclic-nucleotide phosphodiesterase 2 (PDEase 2) (EC 3.1.4.53) | *Saccharomyces cerevisiae* |
| P32329 | Aspartic proteinase 3 (EC 3.4.23.41) | *Saccharomyces cerevisiae* |
| P22276 | DNA-directed RNA polymerase III subunit RPC2 (RNA polymerase III subunit C2) (EC 2.7.7.6) | *Saccharomyces cerevisiae* |
| P13188 | Glutamine--tRNA ligase (EC 6.1.1.18) | *Saccharomyces cerevisiae* |
| Q01476 | Ubiquitin carboxyl-terminal hydrolase 2 (EC 3.4.19.12) | *Saccharomyces cerevisiae* |
| P12689 | DNA repair protein REV1 (EC 2.7.7.-) (Reversionless protein 1) | *Saccharomyces cerevisiae* |
| Q12524 | Peroxisomal coenzyme A diphosphatase 1, peroxisomal (EC 3.6.1.55) | *Saccharomyces cerevisiae* |
| P26989 | Glucoamylase GLA1 (EC 3.2.1.3) (1,4-alpha-D-glucan glucohydrolase) (Glucan 1,4-alpha-glucosidase) | *Saccharomycopsis fibuligera* |
| Q05900 | U1 small nuclear ribonucleoprotein C | *Saccharomyces cerevisiae* |
| Q12522 | Eukaryotic translation initiation factor 6 (eIF-6) | *Saccharomyces cerevisiae* |
| Q12034 | Protein SLF1 | *Saccharomyces cerevisiae* |
| Q12159 | RNA annealing protein YRA1 | *Saccharomyces cerevisiae* |
| P36523 | 54S ribosomal protein L15, mitochondrial | *Saccharomyces cerevisiae* |
| Q02863 | Ubiquitin carboxyl-terminal hydrolase 16 (EC 3.4.19.12) | *Saccharomyces cerevisiae* |
| O74666 | SAP3 (Fragment) | *Rhizopus oryzae* |
| O74667 | SAP4 (Fragment) | *Rhizopus oryzae* |
| Q08905 | Ferric reductase transmembrane component 3 (EC 1.16.1.9) | *Saccharomyces cerevisiae* |
| Q02354 | U3 small nucleolar RNA-associated protein 6 | *Saccharomyces cerevisiae* |
| E9P9X9 | YGL027 protein | *Saccharomyces cerevisiae* |
| Q9WVX3 | PenO | *Pediococcus pentosaceus* |
| Q9WVU3 | OrfA (OrfX-like protein) | *Pediococcus pentosaceus* |
| Q02948 | Vacuolar protein sorting-associated protein 30 (Autophagy-related protein 6) | *Saccharomyces cerevisiae* |
| P35817 | Bromodomain-containing factor 1 | *Saccharomyces cerevisiae* |
| Q04182 | ATP-dependent permease PDR15 | *Saccharomyces cerevisiae* |
| Q12455 | Spermine synthase (SPMSY) (EC 2.5.1.22) (Spermidine aminopropyltransferase) | *Saccharomyces cerevisiae* |
| P38067 | Succinate-semialdehyde dehydrogenase [NADP(+)] (SSDH) (EC 1.2.1.16) | *Saccharomyces cerevisiae* |
| P32349 | DNA-directed RNA polymerase III subunit RPC3 | *Saccharomyces cerevisiae* |
| Q99297 | Mitochondrial 2-oxodicarboxylate carrier 2 | *Saccharomyces cerevisiae* |
| P25582 | 27S pre-rRNA (guanosine(2922)-2'-O)-methyltransferase (EC 2.1.1.167) | *Saccharomyces cerevisiae* |
| P25568 | Autophagy-related protein 22 | *Saccharomyces cerevisiae* |
| P00931 | Tryptophan synthase (EC 4.2.1.20) | *Saccharomyces cerevisiae* |
| P39522 | Dihydroxy-acid dehydratase, mitochondrial (DAD) (EC 4.2.1.9) | *Saccharomyces cerevisiae* |
| Q12110 | Uncharacterized protein YLR049C | *Saccharomyces cerevisiae* |
| P19483 | ATP synthase subunit alpha, mitochondrial (ATP synthase F1 subunit alpha) | *Bos taurus (Bovine)* |
| P05631 | ATP synthase subunit gamma, mitochondrial (ATP synthase F1 subunit gamma) | *Bos taurus (Bovine)* |
| Q02486 | ARS-binding factor 2, mitochondrial | *Saccharomyces cerevisiae* |
| P35207 | Antiviral helicase SKI2 (EC 3.6.4.13) (Superkiller protein 2) | *Saccharomyces cerevisiae* |
| P39744 | Nucleolar complex protein 2 | *Saccharomyces cerevisiae* |
| Q07788 | Protein COS7 | *Saccharomyces cerevisiae* |
| E9P8G8 | Alpha-galactosidase (EC 3.2.1.22) (Melibiase) | *Saccharomyces cerevisiae* |
| Q12335 | Protoplast secreted protein 2 | *Saccharomyces cerevisiae* |
| Q9UVZ5 | Orotidine 5'-phosphate decarboxylase (EC 4.1.1.23) | *Saccharomycopsis fibuligera* |
| P15807 | Siroheme biosynthesis protein MET8 [Includes: Precorrin-2 dehydrogenase (EC 1.3.1.76); Sirohydrochlorin ferrochelatase (EC 4.99.1.4)] | *Saccharomyces cerevisiae* |
| P33331 | Nuclear transport factor 2 (NTF-2) (Nuclear transport factor P10) | *Saccharomyces cerevisiae* |
| Q04080 | GPI transamidase component GPI17 | *Saccharomyces cerevisiae* |
| Q04002 | Sister chromatid cohesion protein 2 | *Saccharomyces cerevisiae* |
| P22213 | Protein SLY1 (Suppressor of loss of YPT1 protein 1) | *Saccharomyces cerevisiae* |
| Q08965 | Ribosome biogenesis protein BMS1 | *Saccharomyces cerevisiae* |
| Q8J1L0 | Endo-glucanase RCE3 | *Rhizopus oryzae* |
| P0CY08 | Mating-type protein ALPHA2 (MATalpha2 protein) (Alpha-2 repressor) | *Saccharomyces cerevisiae* |
| P38284 | Increased copper sensitivity protein 2 | *Saccharomyces cerevisiae* |
| Q12158 | Sister chromatid cohesion protein 1 | *Saccharomyces cerevisiae* |
| Q12289 | Mitochondrial carnitine carrier | *Saccharomyces cerevisiae* |
| Q06407 | Rho-type GTPase-activating protein 2 | *Saccharomyces cerevisiae* |
| P33416 | Heat shock protein 78, mitochondrial | *Saccharomyces cerevisiae* |
| P31382 | Dolichyl-phosphate-mannose--protein mannosyltransferase 2 (EC 2.4.1.109) | *Saccharomyces cerevisiae* |
| P25357 | Probable DNA-binding protein SNT1 (SANT domain-containing protein 1) | *Saccharomyces cerevisiae* |
| Q00402 | Nuclear migration protein NUM1 | *Saccharomyces cerevisiae* |
| Q05567 | Sphingosine-1-phosphate lyase (S1PL) (SP-lyase) (ySPL) (EC 4.1.2.27) | *Saccharomyces cerevisiae* |
| P25641 | Putative lipase ATG15 (EC 3.1.1.3) | *Saccharomyces cerevisiae* |
| Q03898 | Filament protein FIN1 (Filaments in between nuclei protein 1) | *Saccharomyces cerevisiae* |
| P09007 | Pre-rRNA-processing protein SRD1 (Suppressor of rRNA-processing defect protein 1) | *Saccharomyces cerevisiae* |
| P25360 | Inorganic phosphate transporter PHO87 | *Saccharomyces cerevisiae* |
| Q03973 | High mobility group protein 1 (High spontaneous mutagenesis protein 2) | *Saccharomyces cerevisiae* |
| Q99314 | Something about silencing protein 5 | *Saccharomyces cerevisiae* |
| P32266 | Dynamin-like GTPase MGM1, mitochondrial | *Saccharomyces cerevisiae* |
| Q08278 | Mediator of RNA polymerase II transcription subunit 7 (Mediator complex subunit 7) | *Saccharomyces cerevisiae* |
| O94742 | 26S proteasome complex subunit SEM1 | *Saccharomyces cerevisiae* |
| Q03785 | Serine/threonine-protein kinase VHS1 (EC 2.7.11.1) | *Saccharomyces cerevisiae* |
| P03879 | Intron-encoded RNA maturase bI4 (RNA maturase SCbI4) | *Saccharomyces cerevisiae* |
| O13570 | Putative uncharacterized protein YPR142C | *Saccharomyces cerevisiae* |
| Q07786 | Sorbitol dehydrogenase 2 (SDH 2) (EC 1.1.1.-) (Polyol dehydrogenase) (Xylitol dehydrogenase) (EC 1.1.1.9) | *Saccharomyces cerevisiae* |
| P38225 | Very long-chain fatty acid transport protein | *Saccharomyces cerevisiae* |
| Q68BN4 | L-lactate dehydrogenase (EC 1.1.1.27) | *Rhizopus oryzae* |
| Q04299 | Probable ADP-ribose 1''-phosphate phosphatase YML087W (EC 3.1.3.84) | *Saccharomyces cerevisiae* |
| Q12223 | DNA repair protein RAD59 | *Saccharomyces cerevisiae* |
| P35179 | Protein transport protein SSS1 (Sec61 complex subunit SSS1) | *Saccharomyces cerevisiae* |
| Q99207 | Nucleolar complex protein 14 (U three protein 2) | *Saccharomyces cerevisiae* |
| P38904 | Protein SPP41 | *Saccharomyces cerevisiae* |
| P32316 | Acetyl-CoA hydrolase (EC 3.1.2.1) | *Saccharomyces cerevisiae* |
| Q3T4F6 | Orf370 | *Rhizopus oryzae* |
| Q3T4F2 | NADH-ubiquinone oxidoreductase chain 3 (EC 7.1.1.2) | *Rhizopus oryzae* |
| Q3T4D6 | NADH-ubiquinone oxidoreductase chain 4 (EC 7.1.1.2) | *Rhizopus oryzae* |
| Q08732 | Serine/threonine-protein kinase HRK1 (EC 2.7.11.1) | *Saccharomyces cerevisiae* |
| P32495 | H/ACA ribonucleoprotein complex subunit NHP2 (High mobility group-like nuclear protein 2) | *Saccharomyces cerevisiae* |
| Q03956 | Regulator of V-ATPase in vacuolar membrane protein 2 | *Saccharomyces cerevisiae* |
| Q12142 | Autophagy-related protein 9 (Cytoplasm to vacuole targeting protein 7) | *Saccharomyces cerevisiae* |
| Q07528 | Autophagy-related protein 20 | *Saccharomyces cerevisiae* |
| P50264 | Polyamine oxidase FMS1 (EC 1.5.3.17) | *Saccharomyces cerevisiae* |
| Q04172 | Sensitive to high expression protein 9, mitochondrial | *Saccharomyces cerevisiae* |
| Q06001 | Factor arrest protein 10 | *Saccharomyces cerevisiae* |
| Q05946 | U3 small nucleolar RNA-associated protein 13 | *Saccharomyces cerevisiae* |
| Q29TN1 | Tubulin beta chain (Fragment) | *Rhizopus oryzae* |
| Q03954 | DASH complex subunit SPC19 | *Saccharomyces cerevisiae* |
| Q12033 | pH-response regulator protein palA/RIM20 (Regulator of IME2 protein 20) | *Saccharomyces cerevisiae* |
| Q03290 | RNA polymerase II transcription factor B subunit 3 | *Saccharomyces cerevisiae* |
| Q03940 | RuvB-like protein 1 (RUVBL1) (EC 3.6.4.12) | *Saccharomyces cerevisiae* |
| P53848 | Folic acid synthesis protein FOL1 | *Saccharomyces cerevisiae* |
| Q06337 | Chromatin modification-related protein EAF1 (ESA1-associated factor 1) | *Saccharomyces cerevisiae* |
| Q08245 | Protein ZEO1 (Zeocin resistance protein 1) | *Saccharomyces cerevisiae* |
| Q04183 | Trafficking protein particle complex II-specific subunit 120 | *Saccharomyces cerevisiae* |
| Q04429 | Protein HLR1 (LRE1 homolog) | *Saccharomyces cerevisiae* |
| Q06632 | Protein CFT1 (Cleavage factor two protein 1) | *Saccharomyces cerevisiae* |
| Q06674 | Protein HIM1 (High induction of mutagenesis protein 1) | *Saccharomyces cerevisiae* |
| Q07660 | Protein BRE4 (Brefeldin A-sensitivity protein 4) | *Saccharomyces cerevisiae* |
| Q08202 | Protein OPI10 | *Saccharomyces cerevisiae* |
| Q08232 | DSC E3 ubiquitin ligase complex subunit 2 | *Saccharomyces cerevisiae* |
| Q08522 | Putative increased recombination centers protein 14 | *Saccharomyces cerevisiae* |
| Q08580 | Peroxisomal membrane protein PEX27 (Peroxin-27) | *Saccharomyces cerevisiae* |
| Q08903 | Putative uncharacterized protein YOR379C | *Saccharomyces cerevisiae* |
| Q12096 | Glucose N-acetyltransferase 1 (EC 2.4.1.-) (N-acetylglucosaminyltransferase) | *Saccharomyces cerevisiae* |
| Q12234 | GRIP domain-containing protein RUD3 (Golgin-related protein 1) | *Saccharomyces cerevisiae* |
| Q6Q560 | Protein ISD11 (Iron-sulfur protein biogenesis, desulfurase-interacting protein 11) | *Saccharomyces cerevisiae* |
| Q99220 | Protein OS-9 homolog | *Saccharomyces cerevisiae* |
| Q9ZZX0 | Intron-encoded DNA endonuclease aI5 beta (EC 3.1.-.-) | *Saccharomyces cerevisiae* |
| Q04311 | Protein VMS1 (VCP/CDC48-associated mitochondrial stress-responsive protein 1) | *Saccharomyces cerevisiae* |
| Q04585 | Uncharacterized sugar kinase YDR109C (EC 2.7.1.-) | *Saccharomyces cerevisiae* |
| Q05580 | E3 ubiquitin-protein ligase HEL2 (EC 2.3.2.27) | *Saccharomyces cerevisiae* |
| Q08951 | AP-3 complex subunit delta (Adaptor-related protein complex 3 subunit delta) | *Saccharomyces cerevisiae* |
| Q05787 | ERAD-associated E3 ubiquitin-protein ligase component HRD3 (HMG-CoA reductase degradation protein 3) | *Saccharomyces cerevisiae* |
| Q05949 | Protein BUR2 (Bypass UAS requirement protein 2) | *Saccharomyces cerevisiae* |
| Q06147 | Sphingoid long chain base kinase 5 (LCB kinase 5) (EC 2.7.1.91) | *Saccharomyces cerevisiae* |
| Q06160 | Protein SPH1 (SPA2 homolog 1) | *Saccharomyces cerevisiae* |
| Q06551 | Palmitoyltransferase ERF2 (EC 2.3.1.225) | *Saccharomyces cerevisiae* |
| Q06704 | Golgin IMH1 (Integrins and myosins homology protein 1) | *Saccharomyces cerevisiae* |
| Q08692 | Outer spore wall protein 1 | *Saccharomyces cerevisiae* |
| Q12252 | Phosphate metabolism protein 7 | *Saccharomyces cerevisiae* |
| Q12271 | Polyphosphatidylinositol phosphatase INP53 (Suppressor of PMA1 protein 2) (Synaptojanin-like protein 3) [Includes: SAC1-like phosphoinositide phosphatase (EC 3.1.3.-); Phosphatidylinositol 4,5-bisphosphate 5-phosphatase (EC 3.1.3.36)] | *Saccharomyces cerevisiae* |
| Q12420 | 66 kDa U4/U6.U5 small nuclear ribonucleoprotein component | *Saccharomyces cerevisiae* |
| Q06436 | RING-finger protein MAG2 | *Saccharomyces cerevisiae* |
| Q08986 | S-adenosylmethionine permease SAM3 (S-adenosylmethionine metabolism protein 3) | *Saccharomyces cerevisiae* |
| Q12466 | Tricalbin-1 | *Saccharomyces cerevisiae* |
| Q03071 | Elongin-C | *Saccharomyces cerevisiae* |
| Q03195 | Translation initiation factor RLI1 | *Saccharomyces cerevisiae* |
| Q05899 | Uncharacterized vacuolar protein YLR297W | *Saccharomyces cerevisiae* |
| Q06466 | Putative vacuolar protein sorting-associated protein TDA6 (Topoisomerase I damage affected protein 6) | *Saccharomyces cerevisiae* |
| Q06497 | Peroxisomal adenine nucleotide transporter 1 | *Saccharomyces cerevisiae* |
| Q07451 | Endoplasmic reticulum transmembrane protein 3 | *Saccharomyces cerevisiae* |
| Q12040 | Broad-specificity phosphatase YOR283W (EC 3.1.3.-) | *Saccharomyces cerevisiae* |
| Q12043 | Lipase 5 (EC 3.1.1.3) (Triacylglycerol lipase 5) | *Saccharomyces cerevisiae* |
| Q12063 | Dehydrodolichyl diphosphate synthase complex subunit NUS1 (EC 2.5.1.87) | *Saccharomyces cerevisiae* |
| Q12156 | Protein AIM7 (Altered inheritance rate of mitochondria protein 7) | *Saccharomyces cerevisiae* |
| Q12164 | Pore membrane protein of 33 kDa | *Saccharomyces cerevisiae* |
| Q12298 | Uncharacterized ABC transporter ATP-binding protein YDR061W | *Saccharomyces cerevisiae* |
| Q12321 | Mediator of RNA polymerase II transcription subunit 1 (Mediator complex subunit 1) | *Saccharomyces cerevisiae* |
| Q12754 | Ribosomal RNA-processing protein 12 | *Saccharomyces cerevisiae* |
| P38835 | PH domain-containing protein YHR131C | *Saccharomyces cerevisiae* |
| P38688 | Signal recognition particle subunit SRP72 (Signal recognition particle 72 kDa protein homolog) | *Saccharomyces cerevisiae* |
| P19658 | Exocyst complex component EXO70 (Exocyst complex protein of 70 kDa) | *Saccharomyces cerevisiae* |
| Q86ZR7 | Uncharacterized hydrolase YKL033W-A (EC 3.-.-.-) | *Saccharomyces cerevisiae* |
| P36531 | 54S ribosomal protein L36, mitochondrial (Mitochondrial large ribosomal subunit protein bL31m) (YmL36) | *Saccharomyces cerevisiae* |
| Q3E754 | 40S ribosomal protein S21-B (S26) (Small ribosomal subunit protein eS21-B) (YS25) | *Saccharomyces cerevisiae* |
| P38210 | Chromatin structure-remodeling complex protein RSC14 (Low dye-binding protein 7) (Remodel the structure of chromatin complex subunit 14) | *Saccharomyces cerevisiae* |
| P03873 | Cytochrome b mRNA maturase bI2 | *Saccharomyces cerevisiae* |
| P27680 | Ubiquinone biosynthesis O-methyltransferase, mitochondrial | *Saccharomyces cerevisiae* |
| P38842 | UPF0641 membrane protein YHR140W | *Saccharomyces cerevisiae* |
| Q12000 | Translation machinery-associated protein 46 (DRG family-regulatory protein 1) | *Saccharomyces cerevisiae* |
| P15442 | eIF-2-alpha kinase GCN2 (EC 2.7.11.1) | *Saccharomyces cerevisiae* |
| P39073 | Meiotic mRNA stability protein kinase SSN3 (EC 2.7.11.22) (EC 2.7.11.23) (Cyclin-dependent kinase 8) (Suppressor of RNA polymerase B SRB10) | *Saccharomyces cerevisiae* |
| Q03GQ0 | Sigma70_r4 domain-containing protein | *Pediococcus pentosaceus* |
| Q03G65 | Uncharacterized protein | *Pediococcus pentosaceus* |
| Q03G09 | Uncharacterized protein | *Pediococcus pentosaceus* |
| A0JJS2 | Putative carotene-dioxygenase | *Rhizopus oryzae* |
| P36169 | Suppressor of lethality of KEX2 GAS1 double null mutant protein 1 | *Saccharomyces cerevisiae* |
| Q03D71 | ATP-dependent helicase/nuclease subunit A (EC 3.1.-.-) (EC 3.6.4.12) (ATP-dependent helicase/nuclease AddA) | *Pediococcus pentosaceus* |
| Q03E40 | Glutamate--tRNA ligase (EC 6.1.1.17) (Glutamyl-tRNA synthetase) (GluRS) | *Pediococcus pentosaceus* |
| Q03E41 | Cysteine--tRNA ligase (EC 6.1.1.16) (Cysteinyl-tRNA synthetase) (CysRS) | *Pediococcus pentosaceus* |
| Q03EB4 | Elongation factor G (EF-G) | *Pediococcus pentosaceus* |
| Q03EG6 | Aspartyl/glutamyl-tRNA(Asn/Gln) amidotransferase subunit B (Asp/Glu-ADT subunit B) (EC 6.3.5.-) | *Pediococcus pentosaceus* |
| Q03F34 | GTPase Obg (EC 3.6.5.-) (GTP-binding protein Obg) | *Pediococcus pentosaceus* |
| Q03F36 | Ribonuclease Z (RNase Z) (EC 3.1.26.11) (tRNA 3 endonuclease) (tRNase Z) | *Pediococcus pentosaceus* |
| Q03GG4 | Leucine--tRNA ligase (EC 6.1.1.4) (Leucyl-tRNA synthetase) (LeuRS) | *Pediococcus pentosaceus* |
| Q03HF6 | Ribosomal RNA small subunit methyltransferase A (EC 2.1.1.182) | *Pediococcus pentosaceus* |
| P25373 | Glutaredoxin-1 (EC 1.11.1.9) (EC 2.5.1.18) | *Saccharomyces cerevisiae* |
| P42212 | Green fluorescent protein | *Aequorea victoria (Jellyfish)* |
| P12904 | 5'-AMP-activated protein kinase subunit gamma (AMPK gamma) | *Saccharomyces cerevisiae* |
| P40479 | Dual-specificity protein phosphatase SDP1 (EC 3.1.3.48) | *Saccharomyces cerevisiae* |
| A7A0W3 | alpha-1,2-Mannosidase (EC 3.2.1.-) | *Saccharomyces cerevisiae* |
| A6ZZF6 | Probable serine/threonine-protein kinase KKQ8 (EC 2.7.11.1) | *Saccharomyces cerevisiae* |
| A6ZNU1 | Translation machinery associated protein | *Saccharomyces cerevisiae* |
| P38922 | Protein HRB1 (Protein TOM34) | *Saccharomyces cerevisiae* |
| P53919 | H/ACA ribonucleoprotein complex non-core subunit NAF1 (Nuclear assembly factor 1) | *Saccharomyces cerevisiae* |
| P05373 | Delta-aminolevulinic acid dehydratase (ALADH) (EC 4.2.1.24) (Porphobilinogen synthase) | *Saccharomyces cerevisiae* |
| Q6B0Z2 | Putative uncharacterized protein YML101C-A | *Saccharomyces cerevisiae* |
| P40040 | Protein THO1 | *Saccharomyces cerevisiae* |
| P40969 | Centromere DNA-binding protein complex CBF3 subunit B (Centromere protein 3) | *Saccharomyces cerevisiae* |
| A7A179 | Sterol 3-beta-glucosyltransferase (EC 2.4.1.173) | *Saccharomyces cerevisiae* |
| Q08649 | Histone acetyltransferase ESA1 (EC 2.3.1.48) | *Saccharomyces cerevisiae* |
| P38626 | NADH-cytochrome b5 reductase 1 (EC 1.6.2.2) | *Saccharomyces cerevisiae* |
| P12962 | Cap-associated protein CAF20 | *Saccharomyces cerevisiae* |
| P03877 | Intron-encoded DNA endonuclease aI3 (DNA endonuclease I-SceIII) | *Saccharomyces cerevisiae* |
| B3LHB6 | Uncharacterized protein | *Saccharomyces cerevisiae* |
| B3LT04 | DNA repair protein RAD5 | *Saccharomyces cerevisiae* |
| B3LJF6 | Asparagine-rich zinc finger protein AZF1 | *Saccharomyces cerevisiae* |
| B3LKF8 | AA_permease domain-containing protein | *Saccharomyces cerevisiae* |
| B3LKY9 | Tetrameric tRNA splicing endonuclease 54 kDa subunit | *Saccharomyces cerevisiae* |
| B3LL22 | Probable vacuolar protein sorting-associated protein 16 homolog | *Saccharomyces cerevisiae* |
| B3LK76 | Multiple RNA-binding domain-containing protein 1 | *Saccharomyces cerevisiae* |
| B3LPP2 | Protein HRB1 | *Saccharomyces cerevisiae* |
| B3LP03 | SnRNP protein | *Saccharomyces cerevisiae* |
| P07271 | ATP-dependent DNA helicase PIF1 (EC 3.6.4.12) | *Saccharomyces cerevisiae* |
| P43603 | LAS seventeen-binding protein 3 (LAS17-binding protein 3) | *Saccharomyces cerevisiae* |
| B5VTZ8 | Aspartate aminotransferase (EC 2.6.1.1) | *Saccharomyces cerevisiae* |
| B5VTN4 | Signal recognition particle 54 kDa protein | *Saccharomyces cerevisiae* |
| B5VT96 | YPL066Wp-like protein | *Saccharomyces cerevisiae* |
| B5VSY8 | YPL188Wp-like protein | *Saccharomyces cerevisiae* |
| B5VRV4 | Uncharacterized protein (Fragment) | *Saccharomyces cerevisiae* |
| B5VRP7 | YOL007Cp-like protein (Fragment) | *Saccharomyces cerevisiae* |
| B5VQW8 | Uncharacterized protein | *Saccharomyces cerevisiae* |
| B5VQP1 | YNL166Cp-like protein | *Saccharomyces cerevisiae* |
| B5VQC0 | Uncharacterized protein (Fragment) | *Saccharomyces cerevisiae* |
| B5VNS2 | YLR384Cp-like protein (Fragment) | *Saccharomyces cerevisiae* |
| B5VMZ6 | YLR063Wp-like protein | *Saccharomyces cerevisiae* |
| B5VL88 | YJL088Wp-like protein | *Saccharomyces cerevisiae* |
| B5VL61 | YJL112Wp-like protein | *Saccharomyces cerevisiae* |
| B5VK49 | Uncharacterized protein (Fragment) | *Saccharomyces cerevisiae* |
| B5VJW4 | Uncharacterized protein | *Saccharomyces cerevisiae* |
| B5VIJ2 | YGL139Wp-like protein (Fragment) | *Saccharomyces cerevisiae* |
| B5VHE1 | Uncharacterized protein (Fragment) | *Saccharomyces cerevisiae* |
| B5VH18 | YDR490Cp-like protein (Fragment) | *Saccharomyces cerevisiae* |
| B5VGM6 | Uncharacterized protein | *Saccharomyces cerevisiae* |
| B5VGK6 | Uncharacterized protein | *Saccharomyces cerevisiae* |
| B5VFX6 | YDR080Wp-like protein (Fragment) | *Saccharomyces cerevisiae* |
| B5VFD3 | YDL138Wp-like protein (Fragment) | *Saccharomyces cerevisiae* |
| B5VEZ9 | YCR067Cp-like protein (Fragment) | *Saccharomyces cerevisiae* |
| B5VEP4 | Uncharacterized protein | *Saccharomyces cerevisiae* |
| E9P9J7 | Rsf1p | *Saccharomyces cerevisiae* |
| B8YJG1 | Rhizopuspepsin 6 (EC 3.4.23.21) | *Rhizopus oryzae* |
| B8YJG7 | Rhizopuspepsin-like protein (EC 3.4.23.21) | *Rhizopus oryzae* |
| B5VLV9 | ER membrane protein complex subunit 3 (Altered inheritance rate of mitochondria protein 27) | *Saccharomyces cerevisiae* |
| P32639 | Pre-mRNA-splicing helicase BRR2 (EC 3.6.4.13) (Protein Snu246) | *Saccharomyces cerevisiae* |
| C7GY61 | MFS domain-containing protein | *Saccharomyces cerevisiae* |
| C7GVR7 | Ubiquitin carboxyl-terminal hydrolase (EC 3.4.19.12) | *Saccharomyces cerevisiae* |
| C7GUT1 | Hxt11p | *Saccharomyces cerevisiae* |
| C7GUE2 | YJL213W-like protein | *Saccharomyces cerevisiae* |
| C7GP07 | Mnr2p | *Saccharomyces cerevisiae* |
| C7GIL7 | Rpl16bp | *Saccharomyces cerevisiae* |
| P32477 | Glutamate--cysteine ligase (EC 6.3.2.2) | *Saccharomyces cerevisiae* |
| Q8KWJ9 | CPN60 (HSP60) (Fragment) | *Pediococcus pentosaceus* |
| C8Z3N2 | EC1118_1A20_0133p | *Saccharomyces cerevisiae* |
| C8Z3H4 | EC1118_1A20_0815p | *Saccharomyces cerevisiae* |
| C8Z4F1 | EC1118_1C17_1475p | *Saccharomyces cerevisiae* |
| C8Z4M9 | EC1118_1D0_1915p | *Saccharomyces cerevisiae* |
| C8Z534 | EC1118_1D0_3719p | *Saccharomyces cerevisiae* |
| C8Z5F0 | EC1118_1D0_5127p | *Saccharomyces cerevisiae* |
| C8Z6W6 | EC1118_1E8_0529p | *Saccharomyces cerevisiae* |
| C8Z7L7 | EC1118_1F14_0078p | *Saccharomyces cerevisiae* |
| C8Z7L8 | EC1118_1F14_0089p | *Saccharomyces cerevisiae* |
| C8Z7P0 | EC1118_1F14_0364p | *Saccharomyces cerevisiae* |
| C8Z7S4 | EC1118_1F14_0771p | *Saccharomyces cerevisiae* |
| C8Z863 | EC1118_1G1_0881p | *Saccharomyces cerevisiae* |
| C8Z8E0 | EC1118_1G1_1783p | *Saccharomyces cerevisiae* |
| C8Z8H3 | EC1118_1G1_2157p | *Saccharomyces cerevisiae* |
| C8Z8K8 | EC1118_1G1_2597p | *Saccharomyces cerevisiae* |
| C8Z8V8 | EC1118_1G1_3719p | *Saccharomyces cerevisiae* |
| C8Z9C9 | EC1118_1G1_5710p | *Saccharomyces cerevisiae* |
| C8Z9F7 | EC1118_1G1_6029p | *Saccharomyces cerevisiae* |
| C8Z9X3 | EC1118_1H13_1563p | *Saccharomyces cerevisiae* |
| C8ZAV1 | EC1118_1I12_0078p | *Saccharomyces cerevisiae* |
| C8ZAR4 | EC1118_1I12_2223p | *Saccharomyces cerevisiae* |
| C8ZBQ5 | EC1118_1J19_0562p | *Saccharomyces cerevisiae* |
| C8ZCA1 | EC1118_1K5_1717p | *Saccharomyces cerevisiae* |
| C8ZD25 | EC1118_1L10_1277p | *Saccharomyces cerevisiae* |
| C8ZDM4 | Vps63p | *Saccharomyces cerevisiae* |
| C8ZDM9 | EC1118_1L7_1156p | *Saccharomyces cerevisiae* |
| C8ZEK7 | EC1118_1M3_1585p | *Saccharomyces cerevisiae* |
| C8ZEV8 | EC1118_1M3_2773p | *Saccharomyces cerevisiae* |
| C8ZEZ9 | EC1118_1M3_3224p | *Saccharomyces cerevisiae* |
| C8ZFQ2 | EC1118_1N18_0672p | *Saccharomyces cerevisiae* |
| C8ZG24 | EC1118_1N9_1376p | *Saccharomyces cerevisiae* |
| C8ZG35 | EC1118_1N9_1519p | *Saccharomyces cerevisiae* |
| C8ZGC8 | DNA polymerase (EC 2.7.7.7) | *Saccharomyces cerevisiae* |
| C8ZGS1 | EC1118_1O4_3785p | *Saccharomyces cerevisiae* |
| C8ZHE4 | EC1118_1O4_6513p | *Saccharomyces cerevisiae* |
| C8ZHE8 | Hxt13p | *Saccharomyces cerevisiae* |
| C8ZHF5 | EC1118_1O4_6656p | *Saccharomyces cerevisiae* |
| C8ZIH4 | EC1118_1P2_0782p | *Saccharomyces cerevisiae* |
| C8ZJ78 | EC1118_1P2_3730p | *Saccharomyces cerevisiae* |
| C8ZJF9 | EC1118_1P2_4698p | *Saccharomyces cerevisiae* |
| D0UZV8 | SSU1 (Fragment) | *Saccharomyces cerevisiae* |
| P40032 | Prolyl 3,4-dihydroxylase TPA1 (EC 1.14.11.-) (Termination and polyadenylation protein 1) (uS12 prolyl 3,4-dihydroxylase) | *Saccharomyces cerevisiae* |
| C8ZF72 | Acetyl-CoA carboxylase, mitochondrial (ACC) (EC 6.4.1.2) | *Saccharomyces cerevisiae* |
| C8Z478 | EC1118_1C17_0628p | *Saccharomyces cerevisiae* |
| C8Z4A0 | EC1118_1C17_0870p | *Saccharomyces cerevisiae* |
| C8ZFK5 | EC1118_1N18_0122p | *Saccharomyces cerevisiae* |
| C8ZBK2 | EC1118_1J11_2487p | *Saccharomyces cerevisiae* |
| C8ZBK4 | EC1118_1J11_2509p | *Saccharomyces cerevisiae* |
| D3UEG7 | EC1118_1B15_2091p | *Saccharomyces cerevisiae* |
| D3UEM0 | EC1118_1B15_2795p | *Saccharomyces cerevisiae* |
| D3UEN9 | EC1118_1B15_3015p | *Saccharomyces cerevisiae* |
| D3UES8 | EC1118_1B15_3477p | *Saccharomyces cerevisiae* |
| Q03E61 | Uncharacterized protein | *Pediococcus pentosaceus* |
| P0CE70 | ABC transporter NFT1 (New full-length MRP-type transporter 1) | *Saccharomyces cerevisiae* |
| P0CF17 | UPF0507 protein YML002W | *Saccharomyces cerevisiae* |
| P0CF22 | Putative truncated L-serine dehydratase SDL1 (EC 4.3.1.17) | *Saccharomyces cerevisiae* |
| Q08496 | Protein DIA2 (Digs into agar protein 2) | *Saccharomyces cerevisiae* |
| P40965 | MutS protein homolog 4 | *Saccharomyces cerevisiae* |
| P14737 | DNA repair protein RAD9 | *Saccharomyces cerevisiae* |
| P54787 | Vacuolar protein sorting-associated protein 9 | *Saccharomyces cerevisiae* |
| P25655 | General negative regulator of transcription subunit 1 (Cell division cycle protein 39) | *Saccharomyces cerevisiae* |
| C8ZAQ2 | Altered inheritance of mitochondria protein 21 | *Saccharomyces cerevisiae* |
| P39726 | Glycine cleavage system H protein, mitochondrial | *Saccharomyces cerevisiae* |
| P0CI40 | Calcium-binding mitochondrial carrier SAL1 (Suppressor of AAC2 lethality) | *Saccharomyces cerevisiae* |
| P27616 | Phosphoribosylaminoimidazole-succinocarboxamide synthase (EC 6.3.2.6) | *Saccharomyces cerevisiae* |
| E7DZA5 | RNA helicase (Fragment) | *Rhizopus oryzae* |
| G8CYK8 | Triose phosphate transporter (Fragment) | *Rhizopus oryzae* |
| E7E025 | Putative RNA helicase (Fragment) | *Rhizopus oryzae* |
| Q04049 | DNA polymerase eta (EC 2.7.7.7) (Radiation-sensitive protein 30) | *Saccharomyces cerevisiae* |
| C7GRS7 | Altered inheritance of mitochondria protein 11 (Genetic interactor of prohibitins 8) | *Saccharomyces cerevisiae* |
| P0AEX9 | Maltose/maltodextrin-binding periplasmic protein (MMBP) | *Escherichia coli* |
| P25554 | SAGA-associated factor 29 (29 kDa SAGA-associated factor) (SAGA histone acetyltransferase complex 29 kDa subunit) | *Saccharomyces cerevisiae* |
| C8ZAZ9 | VPS10 homolog 2 (Sortilin VTH2) | *Saccharomyces cerevisiae* |
| E7NNK3 | Spindle pole component BBP1 (BFR1-binding protein 1) | *Saccharomyces cerevisiae* |
| E7KFU6 | Protein HRI1 (HRR25-interacting protein 1) | *Saccharomyces cerevisiae* |
| P0CX52 | 40S ribosomal protein S16-B (RP61R) (Small ribosomal subunit protein uS9-B) | *Saccharomyces cerevisiae* |
| P0CX40 | 40S ribosomal protein S8-B (RP19) | *Saccharomyces cerevisiae* |
| E7LYS5 | Protein YIM1 | *Saccharomyces cerevisiae* |
| Q12405 | Peroxisomal membrane protein LPX1 (EC 3.1.1.-) (Lipase of peroxisomes protein 1) | *Saccharomyces cerevisiae* |
| P38244 | Vacuolar membrane protease (EC 3.4.-.-) (FXNA-related family protease 1) | *Saccharomyces cerevisiae* |
| P33893 | Glutamyl-tRNA(Gln) amidotransferase subunit B, mitochondrial (Glu-AdT subunit B) | *Saccharomyces cerevisiae* |
| Q04418 | RNA polymerase II-associated protein RBA50 | *Saccharomyces cerevisiae* |
| Q03049 | Putative uncharacterized oxidoreductase YDR541C (EC 1.1.1.-) | *Saccharomyces cerevisiae* |
| P40028 | Holliday junction resolvase YEN1 (EC 3.1.-.-) | *Saccharomyces cerevisiae* |
| P53238 | Peflin (Penta-EF hand domain-containing protein 1) | *Saccharomyces cerevisiae* |
| Q08281 | Restriction of telomere capping protein 1 (SEH-associated protein 2) | *Saccharomyces cerevisiae* |
| P53874 | Ubiquitin carboxyl-terminal hydrolase 10 (EC 3.4.19.12) | *Saccharomyces cerevisiae* |
| Q06839 | PX domain-containing protein YPR097W | *Saccharomyces cerevisiae* |
| Q08234 | Uncharacterized ABC transporter ATP-binding protein/permease YOL075C | *Saccharomyces cerevisiae* |
| P53170 | [Pyruvate dehydrogenase (acetyl-transferring)] kinase 2, mitochondrial (PDK 2) (Pyruvate dehydrogenase kinase 2) (EC 2.7.11.2) (Protein kinase of PDH protein 2) (Pyruvate dehydrogenase complex kinase 2) (PDC kinase 2) ([Pyruvate dehydrogenase [lipoamide]] kinase 2) | *Saccharomyces cerevisiae* |
| P41832 | Protein BNI1 (Pointed projection formation protein 3) | *Saccharomyces cerevisiae* |
| P32454 | Aminopeptidase 2, mitochondrial (AP-II) (Aminopeptidase II) | *Saccharomyces cerevisiae* |
| P43612 | SIT4-associating protein SAP155 | *Saccharomyces cerevisiae* |
| G2WAA9 | K7_00867p | *Saccharomyces cerevisiae* |
| G2WDQ9 | K7_Mds3p | *Saccharomyces cerevisiae* |
| G2WF84 | K7_Ysc84p | *Saccharomyces cerevisiae* |
| G2WG52 | K7_Xbp1p | *Saccharomyces cerevisiae* |
| G2WGY9 | K7_Yjl027cp | *Saccharomyces cerevisiae* |
| G2WH50 | K7_03945p | *Saccharomyces cerevisiae* |
| G2WHT6 | K7_Ykl096c-bp | *Saccharomyces cerevisiae* |
| G2WHY2 | K7_Ykl050cp | *Saccharomyces cerevisiae* |
| G2WI66 | K7_Caf4p | *Saccharomyces cerevisiae* |
| G2WMJ6 | K7_Yol036wp | *Saccharomyces cerevisiae* |
| G2WNB3 | K7_Dga1bp (Fragment) | *Saccharomyces cerevisiae* |
| G2WNK9 | K7_06754p | *Saccharomyces cerevisiae* |
| G2WPJ2 | K7_Ypr013cp | *Saccharomyces cerevisiae* |
| G2WPR4 | K7_Ypr097wp | *Saccharomyces cerevisiae* |
| G2WPW1 | K7_Ypr148cp | *Saccharomyces cerevisiae* |
| P15705 | Heat shock protein STI1 | *Saccharomyces cerevisiae* |
| P38634 | Protein SIC1 (CDK inhibitor p40) | *Saccharomyces cerevisiae* |
| P19736 | Pre-mRNA-splicing factor PRP9 | *Saccharomyces cerevisiae* |
| N1NVX4 | Protein transport protein sec16 | *Saccharomyces cerevisiae* |
| N1P1U9 | Cdc25p | *Saccharomyces cerevisiae* |
| J3U3K0 | EXO5 (Fragment) | *Saccharomyces cerevisiae* |
| J3U3M5 | IRC8 (Fragment) | *Saccharomyces cerevisiae* |
| K9JYR4 | Glycine--tRNA ligase (EC 6.1.1.14) (Fragment) | *Pediococcus pentosaceus* |
| K4I8G4 | Trp1p | *Saccharomyces cerevisiae* |
| P25043 | Proteasome subunit beta type-2 (EC 3.4.25.1) | *Saccharomyces cerevisiae* |
| P38304 | Mediator of RNA polymerase II transcription subunit 8 | *Saccharomyces cerevisiae* |
| P14306 | Carboxypeptidase Y inhibitor (CPY inhibitor) | *Saccharomyces cerevisiae* |
| Q12406 | Actin-related protein 7 (Actin-like protein ARP7) | *Saccharomyces cerevisiae* |
| P13382 | DNA polymerase alpha catalytic subunit A (EC 2.7.7.7) | *Saccharomyces cerevisiae* |
| A0A0Q0U1B2 | DUF956 domain-containing protein | *Pediococcus pentosaceus* |
| Q03E26 | Uncharacterized protein | *Pediococcus pentosaceus* |
| A0A0R2HG55 | 50S ribosomal protein L3 | *Pediococcus pentosaceus* |
| Q03EB6 | 50S ribosomal protein L3 | *Pediococcus pentosaceus* |
| A0A0R2H836 | 30S ribosomal protein S5 | *Pediococcus pentosaceus* |
| Q03ED3 | 30S ribosomal protein S5 | *Pediococcus pentosaceus* |
| A0A0Q1DLM0 | Uncharacterized protein conserved in bacteria | *Pediococcus pentosaceus* |
| Q03EH4 | Uncharacterized protein | *Pediococcus pentosaceus* |
| A0A063XAS0 | DUF2188 domain-containing protein | *Bacillus subtilis* |
| A0A199WBC6 | Uncharacterized protein | *Bacillus subtilis subsp. subtilis* |
| P96595 | Uncharacterized protein YdaT | *Bacillus subtilis* |
| A0A0E2BXK9 | ATP-binding component of an ABC superfamily transporter (EC 3.6.1.3) | *Lactobacillus casei* |
| A0A0R1EWT4 | ABC superfamily ATP binding cassette transporter, ABC protein | *Lactobacillus coryniformis* |
| A0A0R1KLV2 | ABC superfamily ATP binding cassette transporter, ABC protein | *Lactobacillus nodensis* |
| A0A0R2M873 | ABC superfamily ATP binding cassette transporter, ABC protein | *Lactobacillus xiangfangensis* |
| A0A161XQG4 | Glycosyl transferase family 2 | *Lactobacillus collinoides* |
| A0A1E7XHY0 | Putative ABC transporter ATP-binding protein YbhF | *Lactobacillus sunkii* |
| A0A1J3CGD1 | ABC transporter ATP-binding protein (ABC transporter, ATP-binding protein) (Putative ABC transporter ATP-binding protein YbhF) | *Lactobacillus paracasei* |
| A0A1Z5J4S5 | ABC transporter ATP-binding protein | *Lactobacillus silagincola* |
| A0A2K7QZP8 | ABC transporter ATP-binding protein | *Lactobacillus plantarum* |
| X0PSJ7 | ABC superfamily ATP binding cassette transporter, ABC protein | *Lactobacillus composti* |
| R0G5N4 | Uncharacterized protein | *Pediococcus acidilactici D3* |
| A0A2D1KSP5 | NodB homology domain-containing protein | *Lactobacillus coryniformis* |
| Q9WW30 | Mobilization protein (Orf2) | *Pediococcus pentosaceus* |
| Q03HE6 | ABC-type uncharacterized transport system, periplasmic component | *Pediococcus pentosaceus* |
| Q03FS9 | Proline--tRNA ligase (EC 6.1.1.15) (Prolyl-tRNA synthetase) (ProRS) | *Pediococcus pentosaceus* |
| Q03FB0 | ADP-ribose pyrophosphatase | *Pediococcus pentosaceus* |
| P04050 | DNA-directed RNA polymerase II subunit RPB1 (RNA polymerase II subunit 1) | *Saccharomyces cerevisiae* |
| P38902 | DNA-directed RNA polymerase II subunit RPB11 (RNA polymerase II subunit B11) (B13.6) (DNA-directed RNA polymerase II 13.6 kDa polypeptide) | *Saccharomyces cerevisiae* |
| P46951 | Cargo-transport protein YPP1 (Alpha-synuclein protective protein 1) | *Saccharomyces cerevisiae* |
| Q08723 | 26S proteasome regulatory subunit RPN8 | *Saccharomyces cerevisiae* |
| A0A023T619 | Sup35p | *Saccharomyces cerevisiae* |
| A0A021VPL0 | Transposase | *Actinotalea ferrariae CF5-4* |
| A0A162L361 | Transposase | *Tistrella mobilis* |
| P32939 | GTP-binding protein YPT7 | *Saccharomyces cerevisiae* |
| W0HJ53 | Endo-1,4-beta-xylanase 2 (Xylanase 2) (EC 3.2.1.8) (1,4-beta-D-xylan xylanohydrolase 2) | *Rhizopus oryzae* |
| P07276 | DNA repair protein RAD2 (EC 3.1.-.-) | *Saccharomyces cerevisiae* |
| A0A023PXH4 | Putative uncharacterized membrane protein YAL026C-A | *Saccharomyces cerevisiae* |
| A0A023PYD0 | Putative uncharacterized membrane protein YAL059C-A | *Saccharomyces cerevisiae* |
| P25638 | TPR repeat-containing protein associated with Hsp90 | *Saccharomyces cerevisiae* |
| P32610 | V-type proton ATPase subunit D (V-ATPase subunit D) | *Saccharomyces cerevisiae* |
| A0A0H3WI22 | Uncharacterized protein | *Saccharomyces cerevisiae* |
| P53256 | Exosome complex component RRP46 (Ribosomal RNA-processing protein 46) | *Saccharomyces cerevisiae* |
| P38792 | Exosome complex component RRP4 (Ribosomal RNA-processing protein 4) | *Saccharomyces cerevisiae* |
| Q12149 | Exosome complex exonuclease RRP6 (EC 3.1.13.-) (Ribosomal RNA-processing protein 6) | *Saccharomyces cerevisiae* |
| A0A0N7KZM5 | Ferulic acid decarboxylase | *Saccharomyces cerevisiae* |
| Q06135 | 1,3-beta-glucanosyltransferase GAS2 (EC 2.4.1.-) (Glycolipid-anchored surface protein 2) | *Saccharomyces cerevisiae* |
| A0A1S5VAX2 | Bre5 (Fragment) | *Saccharomyces cerevisiae* |
| A0A1Y0VSQ2 | Tnp_DDE_dom domain-containing protein | *Pediococcus pentosaceus* |
| A0A1Y0VNB6 | GFO_IDH_MocA domain-containing protein | *Pediococcus pentosaceus* |
| A0A1Y0VTG1 | Cytosine deaminase (EC 3.5.4.1) | *Pediococcus pentosaceus* |
| A0A1Y0VTI9 | Phosphoribosylformylglycinamidine synthase subunit PurL (FGAM synthase) (EC 6.3.5.3) | *Pediococcus pentosaceus* |
| A0A1Y0VVG5 | Chaperone protein ClpB | *Pediococcus pentosaceus* |
| A0A1Y0VNA7 | General L-amino acid transport ATP-binding protein AapP (EC 3.6.3.-) | *Pediococcus pentosaceus* |
| A0A1Y0VP53 | AAA domain-containing protein | *Pediococcus pentosaceus* |
| A0A1Y0VWJ9 | Chromosome partition protein Smc | *Pediococcus pentosaceus* |
| A0A1Y0VP75 | Putative ribosome bioproteinis GTPase RsgA (EC 3.6.1.-) | *Pediococcus pentosaceus* |
| A0A1Y0W005 | Dethiobiotin synthase (EC 6.3.3.3) | *Pediococcus pentosaceus* |
| A0A1Y0VWZ0 | Arsenate reductase (Glutaredoxin) (EC 1.20.4.1) | *Pediococcus pentosaceus* |
| A0A1Y0W284 | Putative transposase for insertion-like sequence element IS1161 | *Pediococcus pentosaceus* |
| C1KGC4 | Pediocin (Fragment) | *Pediococcus pentosaceus* |
| P01044 | Kininogen-1 (Kininogen I) (Thiol proteinase inhibitor) | *Bos taurus (Bovine)* |
| P06623 | 2',3'-cyclic-nucleotide 3'-phosphodiesterase (CNP) (CNPase) (EC 3.1.4.37) | *Bos taurus (Bovine)* |
| P23196 | DNA-(apurinic or apyrimidinic site) lyase (EC 3.1.-.-) (EC 4.2.99.18) (APEX nuclease) (APEN) (Apurinic-apyrimidinic endonuclease 1) (AP endonuclease 1) (REF-1) (Redox factor-1) | *Bos taurus (Bovine)* |
| P39873 | Brain ribonuclease (BRB) (EC 3.1.27.-) | *Bos taurus (Bovine)* |
| P32660 | Phospholipid-transporting ATPase DNF1 (EC 7.6.2.1) | *Saccharomyces cerevisiae* |
| Q01888 | Graves disease carrier protein (GDC) | *Bos taurus (Bovine)* |
| P28801 | Glutathione S-transferase P (EC 2.5.1.18) (GST class-pi) | *Bos taurus (Bovine)* |
| P17667 | Myogenic factor 5 (Myf-5) | *Bos taurus (Bovine)* |
| P14763 | Thyrotropin receptor (Thyroid-stimulating hormone receptor) (TSH-R) | *Canis lupus familiaris* |
| P17322 | Endothelin-1 (ET-1) | *Bos taurus (Bovine)* |
| P08487 | 1-phosphatidylinositol 4,5-bisphosphate phosphodiesterase gamma-1 (EC 3.1.4.11) | *Bos taurus (Bovine)* |
| P11834 | Opioid-binding protein/cell adhesion molecule (OBCAM) | *Bos taurus (Bovine)* |
| P00720 | Endolysin (EC 3.2.1.17) (Lysis protein) (Lysozyme) (Muramidase) | *Enterobacteria phage T4* |
| Q08491 | Superkiller protein 7 | *Saccharomyces cerevisiae* |
| A0A0E1L7W5 | Uncharacterized protein | *Escherichia coli* |
| P98131 | L-selectin (CD62 antigen-like family member L) | *Bos taurus (Bovine)* |
| P14893 | 3 beta-hydroxysteroid dehydrogenase/Delta 5-->4-isomerase (3-beta-HSD) | *Bos taurus (Bovine)* |
| P08831 | Interleukin-1 alpha (IL-1 alpha) | *Bos taurus (Bovine)* |
| Q8MIH4 | Alpha s1 casein | *Capra hircus (Goat)* |
| P23004 | Cytochrome b-c1 complex subunit 2, mitochondrial (Complex III subunit 2) | *Bos taurus (Bovine)* |
| Q161M1 | Racemase, putative | *Roseobacter denitrificans* |
| P19782 | Protamine-2 (Sperm histone P2) (Sperm protamine P2) | *Bos taurus (Bovine)* |
| P11614 | 5-hydroxytryptamine receptor 1D (5-HT-1D) (5-HT1D) (Serotonin receptor 1D) | *Canis lupus familiaris* |
| P22507 | Beta-glucosidase 2 (EC 3.2.1.21) (Beta-D-glucoside glucohydrolase) (Cellobiase) (Gentiobiase) | *Saccharomycopsis fibuligera* |
| P12685 | High-affinity potassium transport protein | *Saccharomyces cerevisiae* |
| P33314 | Inhibitory regulator protein BUD2/CLA2 (Bud site selection protein 2) | *Saccharomyces cerevisiae* |
| P36022 | Dynein heavy chain, cytoplasmic (Dynein heavy chain, cytosolic) (DYHC) | *Saccharomyces cerevisiae* |
| P36161 | Nucleoporin NUP133 (Nuclear pore protein NUP133) | *Saccharomyces cerevisiae* |
| P36009 | Probable ATP-dependent RNA helicase DHR2 (EC 3.6.4.13) | *Saccharomyces cerevisiae* |
| P53438 | Protein SOK2 | *Saccharomyces cerevisiae* |
| P50090 | Kelch repeat-containing protein 2 | *Saccharomyces cerevisiae* |
| O74664 | SAP1 (Fragment) | *Rhizopus oryzae* |
| P32784 | Glycerol-3-phosphate O-acyltransferase 1 (G-3-P acyltransferase 1) (EC 2.3.1.15) | *Saccharomyces cerevisiae* |
| Q12276 | HMG2-induced ER-remodeling protein 1 | *Saccharomyces cerevisiae* |
| C8ZH46 | EC1118_1O4_5303p | *Saccharomyces cerevisiae* |
| E7E023 | Putative BTB protein | *Rhizopus oryzae* |
| E7LYB2 | Eisosome protein 1 | *Saccharomyces cerevisiae* |
| P38088 | Glycine--tRNA ligase 1, mitochondrial (EC 6.1.1.14) | *Saccharomyces cerevisiae* |
| P36130 | CCR4-associated factor 4 | *Saccharomyces cerevisiae* |
| P07991 | Ornithine aminotransferase (EC 2.6.1.13) | *Saccharomyces cerevisiae* |
| P08566 | Pentafunctional AROM polypeptide [Includes: 3-dehydroquinate synthase (DHQS) (EC 4.2.3.4); 3-phosphoshikimate 1-carboxyvinyltransferase (EC 2.5.1.19) (5-enolpyruvylshikimate-3-phosphate synthase) (EPSP synthase) (EPSPS); Shikimate kinase (SK) (EC 2.7.1.71); 3-dehydroquinate dehydratase (3-dehydroquinase) (EC 4.2.1.10); Shikimate dehydrogenase (EC 1.1.1.25)] | *Saccharomyces cerevisiae* |
| P21951 | DNA polymerase epsilon catalytic subunit A (EC 2.7.7.7) | *Saccharomyces cerevisiae* |
| P14284 | DNA polymerase zeta catalytic subunit (EC 2.7.7.7) (Protein reversionless 3) | *Saccharomyces cerevisiae* |
| P03870 | Site-specific recombinase Flp (FLP) (Protein Able) | *Saccharomyces cerevisiae* |
| P20459 | Eukaryotic translation initiation factor 2 subunit alpha (eIF-2-alpha) | *Saccharomyces cerevisiae* |
| P22147 | 5'-3' exoribonuclease 1 (EC 3.1.13.-) (DNA strand transfer protein beta) (STP-beta) | *Saccharomyces cerevisiae* |
| P06106 | Homocysteine/cysteine synthase (EC 2.5.1.47) (EC 2.5.1.49) | *Saccharomyces cerevisiae* |
| P21372 | Pre-mRNA-processing ATP-dependent RNA helicase PRP5 (EC 3.6.4.13) | *Saccharomyces cerevisiae* |
| P12687 | 54S ribosomal protein L2, mitochondrial (Mitochondrial large ribosomal subunit protein bL27m) | *Saccharomyces cerevisiae* |
| P29496 | Minichromosome maintenance protein 5 (EC 3.6.4.12) | *Saccharomyces cerevisiae* |
| P32324 | Elongation factor 2 (EF-2) (Eukaryotic elongation factor 2) | *Saccharomyces cerevisiae* |
| P32502 | Translation initiation factor eIF-2B subunit beta (GCD complex subunit GCD7) | *Saccharomyces cerevisiae* |
| P32179 | 3'(2'),5'-bisphosphate nucleotidase (EC 3.1.3.7) | *Saccharomyces cerevisiae* |
| P32340 | Rotenone-insensitive NADH-ubiquinone oxidoreductase, mitochondrial (EC 1.6.5.9) | *Saccharomyces cerevisiae* |
| P32387 | 54S ribosomal protein L41, mitochondrial | *Saccharomyces cerevisiae* |
| P32902 | 37S ribosomal protein MRP4, mitochondrial | *Saccharomyces cerevisiae* |
| P32259 | Mediator of RNA polymerase II transcription subunit 16 | *Saccharomyces cerevisiae* |
| P32364 | Kinesin-related protein SMY1 (Suppressor protein SMY1) | *Saccharomyces cerevisiae* |
| P32603 | Sporulation-specific glucan 1,3-beta-glucosidase (EC 3.2.1.58) (Exo-1,3-beta-glucanase) | *Saccharomyces cerevisiae* |
| Q03497 | Serine/threonine-protein kinase STE20 (EC 2.7.11.1) | *Saccharomyces cerevisiae* |
| P32367 | Transcription factor tau 95 kDa subunit | *Saccharomyces cerevisiae* |
| P32912 | Vacuolar morphogenesis protein 7 | *Saccharomyces cerevisiae* |
| P33441 | THO complex subunit MFT1 (Mitochondrial fusion target protein 1) | *Saccharomyces cerevisiae* |
| P36135 | Probable secreted beta-glucosidase UTH1 (EC 3.2.1.-) (Youth protein 1) | *Saccharomyces cerevisiae* |
| P32801 | Serine/threonine-protein kinase ELM1 (EC 2.7.11.1) | *Saccharomyces cerevisiae* |
| Q02455 | Protein MLP1 (Myosin-like protein 1) | *Saccharomyces cerevisiae* |
| P36120 | ATP-dependent RNA helicase DBP7 (EC 3.6.4.13) | *Saccharomyces cerevisiae* |
| P36148 | Glycerol-3-phosphate O-acyltransferase 2 (G-3-P acyltransferase 2) (EC 2.3.1.15) | *Saccharomyces cerevisiae* |
| P36097 | TEL2-interacting protein 1 | *Saccharomyces cerevisiae* |
| P36095 | Vacuolar protein-sorting-associated protein 24 (DOA4-independent degradation protein 3) (ESCRT-III complex subunit VPS24) | *Saccharomyces cerevisiae* |
| P36016 | Heat shock protein 70 homolog LHS1 (EC 3.6.1.3) | *Saccharomyces cerevisiae* |
| P36082 | Putative platinum sensitivity protein 1 | *Saccharomyces cerevisiae* |
| P36048 | Pre-mRNA-splicing factor SNU114 (114 kDa U5 small nuclear ribonucleoprotein component) (Growth inhibitory protein 10) | *Saccharomyces cerevisiae* |
| P25328 | RNA-directed RNA polymerase (EC 2.7.7.48) (p91) | *Saccharomyces 20S RNA narnavirus* |
| P38077 | ATP synthase subunit gamma, mitochondrial (F-ATPase gamma subunit) | *Saccharomyces cerevisiae* |
| P38041 | Protein BOB1 (BEM1-binding protein) (Growth inhibitory protein 7) | *Saccharomyces cerevisiae* |
| P29465 | Chitin synthase 3 (EC 2.4.1.16) | *Saccharomyces cerevisiae* |
| P38080 | Serine/threonine-protein kinase AKL1 (EC 2.7.11.1) | *Saccharomyces cerevisiae* |
| P36775 | Lon protease homolog, mitochondrial (EC 3.4.21.53) | *Saccharomyces cerevisiae* |
| P38114 | Uncharacterized transcriptional regulatory protein TBS1 (Thiabendazole sensitive protein 1) | *Saccharomyces cerevisiae* |
| P38297 | Mitofusin FZO1 (EC 3.6.5.-) (Transmembrane GTPase FZO1) | *Saccharomyces cerevisiae* |
| P38205 | Multisite-specific tRNA:(cytosine-C(5))-methyltransferase (EC 2.1.1.202) | *Saccharomyces cerevisiae* |
| P38199 | Heterogeneous nuclear rnp K-like protein 2 (KH domain-containing protein 1) | *Saccharomyces cerevisiae* |
| P38196 | Uridine permease | *Saccharomyces cerevisiae* |
| P38272 | SWI5-dependent HO expression protein 3 | *Saccharomyces cerevisiae* |
| P39010 | Palmitoyltransferase AKR1 (EC 2.3.1.225) | *Saccharomyces cerevisiae* |
| P09119 | Cell division control protein 6 | *Saccharomyces cerevisiae* |
| P21657 | Transcriptional activator protein DAL81 (Regulatory protein UGA35) | *Saccharomyces cerevisiae* |
| P32354 | Minichromosome maintenance protein 10 (Protein DNA43) | *Saccharomyces cerevisiae* |
| P32361 | Serine/threonine-protein kinase/endoribonuclease IRE1 | *Saccharomyces cerevisiae* |
| P38990 | SNF1-activating kinase 1 (EC 2.7.11.1) | *Saccharomyces cerevisiae* |
| P25635 | Periodic tryptophan protein 2 (U three protein 1) | *Saccharomyces cerevisiae* |
| P39538 | Ubiquitin carboxyl-terminal hydrolase 12 (EC 3.4.19.12) | *Saccharomyces cerevisiae* |
| P39968 | Vacuolar protein 8 | *Saccharomyces cerevisiae* |
| P40036 | GLC7-interacting protein 2 | *Saccharomyces cerevisiae* |
| P40071 | Transmembrane 9 superfamily member 3 | *Saccharomyces cerevisiae* |
| P40086 | Cytochrome c oxidase assembly protein COX15 | *Saccharomyces cerevisiae* |
| P40094 | Conserved oligomeric Golgi complex subunit 3 (COG complex subunit 3) | *Saccharomyces cerevisiae* |
| P38737 | Proteasome component ECM29 (Extracellular mutant protein 29) | *Saccharomyces cerevisiae* |
| P38735 | ABC transporter ATP-binding protein/permease VMR1 (Vacuolar multidrug resistance protein 1) | *Saccharomyces cerevisiae* |
| P38766 | ATP-dependent DNA helicase RRM3 (EC 3.6.4.12) | *Saccharomyces cerevisiae* |
| P38710 | Inositol monophosphatase 1 (EC 3.1.3.25) | *Saccharomyces cerevisiae* |
| P38811 | Transcription-associated protein 1 (p400 kDa component of SAGA) | *Saccharomyces cerevisiae* |
| P40557 | ER-retained PMA1-suppressing protein 1 (EC 5.3.4.1) | *Saccharomyces cerevisiae* |
| P40529 | ADP-ribosylation factor GTPase-activating protein effector protein 2 (ARF GAP effector protein 2) | *Saccharomyces cerevisiae* |
| P40460 | Kinetochore protein NDC80 | *Saccharomyces cerevisiae* |
| P07683 | Glucoamylase 1 (Gluc 1) (EC 3.2.1.3) (1,4-alpha-D-glucan glucohydrolase) (Glucan 1,4-alpha-glucosidase) | *Rhizopus oryzae* |
| P05030 | Plasma membrane ATPase 1 (EC 7.1.2.1) (Proton pump 1) | *Saccharomyces cerevisiae* |
| P27614 | Carboxypeptidase S (EC 3.4.17.4) (GLY-X carboxypeptidase) (YSCS) | *Saccharomyces cerevisiae* |
| P36012 | Histone H3-like centromeric protein CSE4 (CENP-A homolog) | *Saccharomyces cerevisiae* |
| P08678 | Adenylate cyclase (EC 4.6.1.1) | *Saccharomyces cerevisiae* |
| P43574 | Transcriptional regulatory protein GAT1 | *Saccharomyces cerevisiae* |
| P41812 | Ribonucleases P/MRP protein subunit POP1 (EC 3.1.26.5) | *Saccharomyces cerevisiae* |
| P23394 | Pre-mRNA-splicing ATP-dependent RNA helicase PRP28 (EC 3.6.4.13) | *Saccharomyces cerevisiae* |
| P43561 | Iron transport multicopper oxidase FET5 (EC 1.-.-.-) | *Saccharomyces cerevisiae* |
| P43549 | Aquaglycerol porin AQY3 (Aquaporin-3) | *Saccharomyces cerevisiae* |
| P42942 | Uncharacterized GTP-binding protein YGR210C | *Saccharomyces cerevisiae* |
| P40985 | Probable E3 ubiquitin-protein ligase HUL4 (EC 2.3.2.26) | *Saccharomyces cerevisiae* |
| P42945 | U3 small nucleolar RNA-associated protein 10 | *Saccharomyces cerevisiae* |
| P42841 | Polyadenylation factor subunit 2 | *Saccharomyces cerevisiae* |
| P46974 | Respiration factor 2 (Zinc finger protein ZMS1) | *Saccharomyces cerevisiae* |
| P49367 | Homoaconitase, mitochondrial (EC 4.2.1.36) | *Saccharomyces cerevisiae* |
| P27929 | 37S ribosomal protein NAM9, mitochondrial | *Saccharomyces cerevisiae* |
| P38931 | Mediator of RNA polymerase II transcription subunit 13 (Mediator complex subunit 13) (Protein SCA1) (Suppressor of RNA polymerase B SSN2) | *Saccharomyces cerevisiae* |
| P32325 | DDK kinase regulatory subunit DBF4 (Dumbbell forming protein 4) | *Saccharomyces cerevisiae* |
| P47052 | Succinate dehydrogenase [ubiquinone] flavoprotein subunit 2, mitochondrial (EC 1.3.5.1) | *Saccharomyces cerevisiae* |
| P47154 | CAAX prenyl protease 1 (EC 3.4.24.84) | *Saccharomyces cerevisiae* |
| P47037 | Structural maintenance of chromosomes protein 3 (DA-box protein SMC3) | *Saccharomyces cerevisiae* |
| P47035 | Nucleolar protein NET1 | *Saccharomyces cerevisiae* |
| P48563 | Protein MON2 | *Saccharomyces cerevisiae* |
| Q00362 | Protein phosphatase PP2A regulatory subunit B | *Saccharomyces cerevisiae* |
| P47136 | Bud site selection protein 4 | *Saccharomyces cerevisiae* |
| P24784 | ATP-dependent RNA helicase DBP1 (EC 3.6.4.13) | *Saccharomyces cerevisiae* |
| P41939 | Isocitrate dehydrogenase [NADP] cytoplasmic (IDH) (EC 1.1.1.42) | *Saccharomyces cerevisiae* |
| P53982 | Isocitrate dehydrogenase [NADP] (IDH) (EC 1.1.1.42) (IDP) (NADP(+)-specific ICDH) (Oxalosuccinate decarboxylase) | *Saccharomyces cerevisiae* |
| P39112 | Exoribonuclease II, mitochondrial (RNase II) (Ribonuclease II) (EC 3.1.13.1) | *Saccharomyces cerevisiae* |
| P32497 | Eukaryotic translation initiation factor 3 subunit C (eIF3c) | *Saccharomyces cerevisiae* |
| P11154 | Pyruvate carboxylase 1 (EC 6.4.1.1) (Pyruvic carboxylase 1) (PCB 1) | *Saccharomyces cerevisiae* |
| P04456 | 60S ribosomal protein L25 | *Saccharomyces cerevisiae* |
| P10662 | 37S ribosomal protein MRP1, mitochondrial (Mitochondrial small ribosomal subunit protein mS43) | *Saccharomyces cerevisiae* |
| P53598 | Succinate--CoA ligase [ADP-forming] subunit alpha, mitochondrial (EC 6.2.1.5) | *Saccharomyces cerevisiae* |
| P53616 | Probable secreted beta-glucosidase SUN4 (EC 3.2.1.-) (Septation protein SUN4) (Soluble cell wall protein 3) | *Saccharomyces cerevisiae* |
| P00958 | Methionine--tRNA ligase, cytoplasmic (EC 6.1.1.10) | *Saccharomyces cerevisiae* |
| P39001 | Transcriptional regulatory protein UME6 (Negative transcriptional regulator of IME2) | *Saccharomyces cerevisiae* |
| P39959 | Zinc finger protein YER130C | *Saccharomyces cerevisiae* |
| P53316 | Uncharacterized RNA-binding protein YGR250C | *Saccharomyces cerevisiae* |
| P53145 | Large subunit GTPase 1 (EC 3.6.1.-) | *Saccharomyces cerevisiae* |
| P53121 | Putative flavin carrier protein 3 (FAD transporter 3) (TRP-like ion channel FLC3) | *Saccharomyces cerevisiae* |
| P53112 | Peroxisomal membrane protein PEX14 (Peroxin-14) | *Saccharomyces cerevisiae* |
| P53101 | Cystathionine beta-lyase (CBL) (EC 4.4.1.13) | *Saccharomyces cerevisiae* |
| P53759 | tRNA-dihydrouridine(16/17) synthase [NAD(P)(+)] (EC 1.3.1.88) | *Saccharomyces cerevisiae* |
| P53742 | Nucleolar GTP-binding protein 2 | *Saccharomyces cerevisiae* |
| P53972 | 25S rRNA (cytosine(2278)-C(5))-methyltransferase (EC 2.1.1.311) | *Saccharomyces cerevisiae* |
| P53943 | Probable transporter AQR1 | *Saccharomyces cerevisiae* |
| P53940 | J domain-containing protein APJ1 | *Saccharomyces cerevisiae* |
| P53924 | E3 ubiquitin-protein ligase DMA2 (EC 2.3.2.27) | *Saccharomyces cerevisiae* |
| Q12434 | Rho GDP-dissociation inhibitor (Rho GDI) | *Saccharomyces cerevisiae* |
| Q04439 | Myosin-5 (Actin-dependent myosin-I MYO5) | *Saccharomyces cerevisiae* |
| Q06706 | Elongator complex protein 1 (Gamma-toxin target 1) | *Saccharomyces cerevisiae* |
| Q12216 | E3 SUMO-protein ligase SIZ2 (EC 2.3.2.-) (E3 SUMO-protein transferase SIZ2) | *Saccharomyces cerevisiae* |
| Q07350 | Pre-mRNA-splicing factor PRP11 | *Saccharomyces cerevisiae* |
| Q05022 | rRNA biogenesis protein RRP5 (Ribosomal RNA-processing protein 5) | *Saccharomyces cerevisiae* |
| Q08446 | Protein SGT1 (Suppressor of G2 allele of SKP1) | *Saccharomyces cerevisiae* |
| Q07878 | Vacuolar protein sorting-associated protein 13 | *Saccharomyces cerevisiae* |
| Q92331 | Vacuolar protein sorting-associated protein 5 (Carboxypeptidase Y-deficient protein 10) | *Saccharomyces cerevisiae* |
| Q12749 | Structural maintenance of chromosomes protein 6 (DNA repair protein RHC18) (Rad18 homolog) | *Saccharomyces cerevisiae* |
| Q05506 | Arginine--tRNA ligase, cytoplasmic (EC 6.1.1.19) | *Saccharomyces cerevisiae* |
| Q03263 | Uncharacterized transporter YMR279C | *Saccharomyces cerevisiae* |
| Q12362 | Bifunctional protein RIB2 [Includes: tRNA pseudouridine(32) synthase, cytoplasmic (EC 5.4.99.28) (tRNA pseudouridine synthase 8) (tRNA pseudouridylate synthase 8) (tRNA-uridine isomerase 8); Diaminohydroxyphosphoribosylaminopyrimidine deaminase (DRAP deaminase) (EC 3.5.4.26) (Riboflavin-specific deaminase)] | *Saccharomyces cerevisiae* |
| P46943 | Translation factor GUF1, mitochondrial (EC 3.6.5.-) (Elongation factor 4 homolog) | *Saccharomyces cerevisiae* |
| P39078 | T-complex protein 1 subunit delta (TCP-1-delta) (CCT-delta) | *Saccharomyces cerevisiae* |
| P22579 | Transcriptional regulatory protein SIN3 | *Saccharomyces cerevisiae* |
| Q04399 | Putative multicopper oxidase GMC1 (EC 1.-.-.-) (Grand meiotic recombination cluster protein 1) | *Saccharomyces cerevisiae* |
| Q03834 | DNA mismatch repair protein MSH6 (MutS protein homolog 6) (Postmeiotic segregation protein 3) | *Saccharomyces cerevisiae* |
| Q00416 | Helicase SEN1 (EC 3.6.4.-) (tRNA-splicing endonuclease positive effector) | *Saccharomyces cerevisiae* |
| Q08968 | Protein adenylyltransferase SelO, mitochondrial (EC 2.7.7.-) (Selenoprotein O) (SelO) | *Saccharomyces cerevisiae* |
| P13186 | Serine/threonine-protein kinase KIN2 (EC 2.7.11.1) | *Saccharomyces cerevisiae* |
| P32526 | Karyogamy protein KAR9 (Cortical protein KAR9) | *Saccharomyces cerevisiae* |
| P32898 | Mitochondrial presequence protease (EC 3.4.24.-) (Cytosolic metalloprotease 1) (Metalloprotease of 112 kDa) | *Saccharomyces cerevisiae* |
| P03875 | Putative COX1/OXI3 intron 1 protein | *Saccharomyces cerevisiae* |
| P03876 | Putative COX1/OXI3 intron 2 protein | *Saccharomyces cerevisiae* |
| P32906 | Endoplasmic reticulum mannosyl-oligosaccharide 1,2-alpha-mannosidase (EC 3.2.1.113) (ER alpha-1,2-mannosidase) (Man(9)-alpha-mannosidase) | *Saccharomyces cerevisiae* |
| Q08484 | GTPase-activating protein GYP1 (GAP for YPT1) | *Saccharomyces cerevisiae* |
| Q03784 | Trafficking protein particle complex subunit 23 (TRAPP subunit 23) (Transport protein particle 23 kDa subunit) | *Saccharomyces cerevisiae* |
| P16120 | Threonine synthase (TS) (EC 4.2.3.1) | *Saccharomyces cerevisiae* |
| P54115 | Magnesium-activated aldehyde dehydrogenase, cytosolic (EC 1.2.1.4) (Mg(2+)-activated acetaldehyde dehydrogenase) (Mg(2+)-ACDH) | *Saccharomyces cerevisiae* |
| P48415 | COPII coat assembly protein SEC16 (Protein transport protein SEC16) | *Saccharomyces cerevisiae* |
| Q12019 | Midasin (Dynein-related AAA-ATPase REA1) (MIDAS-containing protein) (Ribosome export/assembly protein 1) | *Saccharomyces cerevisiae* |
| Q12361 | G protein-coupled receptor GPR1 | *Saccharomyces cerevisiae* |
| P47068 | Myosin tail region-interacting protein MTI1 (Protein BBC1) | *Saccharomyces cerevisiae* |
| P29547 | Elongation factor 1-gamma 1 (EF-1-gamma 1) (Calcium and membrane-binding protein 1) (Calcium phospholipid-binding protein) (CPBP) (Eukaryotic elongation factor 1Bgamma 1) (eEF1Bgamma 1) (Translation elongation factor 1B gamma 1) | *Saccharomyces cerevisiae* |
| E9P8D6 | Vacuolar membrane ATPase subunit a (Fragment) | *Saccharomyces cerevisiae* |
| Q12178 | Cytosine deaminase (EC 3.5.4.1) (Cytosine aminohydrolase) | *Saccharomyces cerevisiae* |
| P46680 | Actin-interacting protein 1 | *Saccharomyces cerevisiae* |
| P25623 | Suppressor of yeast profilin deletion | *Saccharomyces cerevisiae* |
| Q03280 | E3 ubiquitin-protein ligase TOM1 (EC 2.3.2.26) (HECT-type E3 ubiquitin transferase TOM1) (Suppressor of snRNA protein 2) (Temperature-dependent organization in mitotic nucleus protein 1) | *Saccharomyces cerevisiae* |
| P53342 | Putative uncharacterized protein YGR293C | *Saccharomyces cerevisiae* |
| P39683 | Nicotinate phosphoribosyltransferase (NAPRTase) (EC 6.3.4.21) | *Saccharomyces cerevisiae* |
| Q12280 | Ras GTPase-activating-like protein IQG1 (Cytokinesis protein 1) | *Saccharomyces cerevisiae* |
| P25386 | Intracellular protein transport protein USO1 (Int-1) | *Saccharomyces cerevisiae* |
| Q08224 | Hydroxymethylpyrimidine/phosphomethylpyrimidine kinase THI20 (EC 2.7.1.49) (EC 2.7.4.7) (Hydroxymethylpyrimidine kinase) (HMP kinase) (Hydroxymethylpyrimidine phosphate kinase) (HMP-P kinase) (HMP-phosphate kinase) (HMPP kinase) | *Saccharomyces cerevisiae* |
| Q06628 | Autophagy-related protein 13 | *Saccharomyces cerevisiae* |
| Q12527 | Autophagy-related protein 11 (Cytoplasm to vacuole targeting protein 9) | *Saccharomyces cerevisiae* |
| Q12518 | Epsin-1 | *Saccharomyces cerevisiae* |
| Q12309 | Pre-mRNA-splicing factor CLF1 (Crooked neck-like factor 1) | *Saccharomyces cerevisiae* |
| Q05468 | Ribosome quality control complex subunit 1 | *Saccharomyces cerevisiae* |
| Q06681 | Membrane-anchored lipid-binding protein YSP2 | *Saccharomyces cerevisiae* |
| Q07508 | Protein LUC7 | *Saccharomyces cerevisiae* |
| Q08650 | Diacylglycerol O-acyltransferase 1 (DGAT) (EC 2.3.1.20) (Acyl-CoA:monoacylglycerol acyltransferase) (MGAT) (EC 2.3.1.22) | *Saccharomyces cerevisiae* |
| Q08949 | DNA damage checkpoint protein 1 | *Saccharomyces cerevisiae* |
| Q12028 | AP-1 complex subunit gamma-1 (Clathrin assembly protein complex 1 gamma large chain) | *Saccharomyces cerevisiae* |
| Q07457 | E3 ubiquitin-protein ligase BRE1 (EC 2.3.2.27) | *Saccharomyces cerevisiae* |
| Q07527 | tRNA (guanosine(18)-2'-O)-methyltransferase (EC 2.1.1.34) | *Saccharomyces cerevisiae* |
| Q08204 | Structural maintenance of chromosomes protein 5 | *Saccharomyces cerevisiae* |
| Q12344 | GTPase-activating protein GYP5 | *Saccharomyces cerevisiae* |
| Q06315 | Protein SKG3 (Suppressor of lethality of KEX2-GAS1 double null mutant protein 3) | *Saccharomyces cerevisiae* |
| Q06698 | Putative ATP-dependent RNA helicase YLR419W (EC 3.6.4.13) | *Saccharomyces cerevisiae* |
| Q07825 | Putative Xaa-Pro aminopeptidase FRA1 (EC 3.4.11.9) | *Saccharomyces cerevisiae* |
| Q03016 | GLC7-interacting protein 3 | *Saccharomyces cerevisiae* |
| Q06623 | HDA1 complex subunit 3 (Histone deacetylase complex 1 subunit 3) | *Saccharomyces cerevisiae* |
| Q12139 | Zinc finger protein YPR022C | *Saccharomyces cerevisiae* |
| Q12354 | Acyl-protein thioesterase 1 (EC 3.1.2.-) | *Saccharomyces cerevisiae* |
| Q12532 | Ribosome quality control complex subunit 2 (Translation-associated element 2) | *Saccharomyces cerevisiae* |
| Q2VER8 | Glucoamylase b | *Rhizopus oryzae* |
| P38345 | Succinate dehydrogenase assembly factor 4, mitochondrial (SDH assembly factor 4) (SDHAF4) (Found in mitochondrial proteome protein 21) | *Saccharomyces cerevisiae* |
| P43610 | Uncharacterized ATP-dependent helicase IRC5 (EC 3.6.4.-) (Increased recombination centers protein 5) | *Saccharomyces cerevisiae* |
| Q1W284 | Alpha-aminoadipate reductase (EC 1.2.1.31) | *Saccharomycopsis fibuligera* |
| P0C155 | Putative carboxypeptidase YOL153C (EC 3.4.17.-) | *Saccharomyces cerevisiae* |
| P25569 | Glucose-induced degradation protein 7 | *Saccharomyces cerevisiae* |
| P19158 | Inhibitory regulator protein IRA2 | *Saccharomyces cerevisiae* |
| Q12442 | ADIPOR-like receptor IZH2 (Phosphate metabolism protein 36) | *Saccharomyces cerevisiae* |
| P53088 | Cytoplasmic tRNA 2-thiolation protein 1 (EC 2.7.7.-) (Cytoplasmic tRNA adenylyltransferase 1) | *Saccharomyces cerevisiae* |
| P36126 | Phospholipase D1 (PLD 1) (EC 3.1.4.4) (Choline phosphatase 1) (Meiosis-specific sporulation-specific protein 14) (Phosphatidylcholine-hydrolyzing phospholipase D1) | *Saccharomyces cerevisiae* |
| Q03EB0 | DNA-directed RNA polymerase subunit beta' (RNAP subunit beta') (EC 2.7.7.6) | *Pediococcus pentosaceus* |
| Q03FM6 | Formate--tetrahydrofolate ligase (EC 6.3.4.3) | *Pediococcus pentosaceus* |
| Q03FZ4 | Exodeoxyribonuclease 7 large subunit (EC 3.1.11.6) | *Pediococcus pentosaceus* |
| Q03GY3 | Glycerol-3-phosphate dehydrogenase [NAD(P)+] (EC 1.1.1.94) | *Pediococcus pentosaceus* |
| Q08685 | mRNA cleavage and polyadenylation factor CLP1 | *Saccharomyces cerevisiae* |
| Q99315 | Transposon Ty3-G Gag-Pol polyprotein (Gag3-Pol3) | *Saccharomyces cerevisiae* |
| P0C2H7 | 60S ribosomal protein L27-B (Large ribosomal subunit protein eL27-B) | *Saccharomyces cerevisiae* |
| Q12337 | Transposon Ty2-GR1 Gag-Pol polyprotein (TY2A-TY2B) | *Saccharomyces cerevisiae* |
| A6ZRJ6 | Conserved protein | *Saccharomyces cerevisiae* |
| P04807 | Hexokinase-2 (EC 2.7.1.1) (Hexokinase PII) (Hexokinase-B) | *Saccharomyces cerevisiae* |
| P00950 | Phosphoglycerate mutase 1 (PGAM 1) (EC 5.4.2.11) | *Saccharomyces cerevisiae* |
| P00817 | Inorganic pyrophosphatase (EC 3.6.1.1) | *Saccharomyces cerevisiae* |
| A9QGY6 | Translation elongation factor 1-alpha (Fragment) | *Saccharomycopsis fibuligera* |
| Q07888 | Y' element ATP-dependent helicase YLL067C (EC 3.6.4.12) | *Saccharomyces cerevisiae* |
| P28834 | Isocitrate dehydrogenase [NAD] subunit 1, mitochondrial (EC 1.1.1.41) | *Saccharomyces cerevisiae* |
| Q06053 | tRNA-dihydrouridine(47) synthase [NAD(P)(+)] (EC 1.3.1.89) | *Saccharomyces cerevisiae* |
| P46997 | J protein JJJ2 | *Saccharomyces cerevisiae* |
| B5VTP4 | YPR103Wp-like protein | *Saccharomyces cerevisiae* |
| B5VS98 | Uncharacterized protein | *Saccharomyces cerevisiae* |
| B5VMB4 | YKL042Wp-like protein | *Saccharomyces cerevisiae* |
| B5VKT6 | YIR001Cp-like protein | *Saccharomyces cerevisiae* |
| P42937 | CDC25-like phosphatase YCH1 (EC 3.1.3.-) (CDC25 homolog 1) | *Saccharomyces cerevisiae* |
| B7XC04 | Glucoamylase | *Rhizopus oryzae* |
| B8YJG3 | Rhizopuspepsin 2 (EC 3.4.23.21) | *Rhizopus oryzae* |
| B9W3V1 | Uncharacterized protein | *Pediococcus pentosaceus* |
| C8Z4W2 | EC1118_1D0_2861p | *Saccharomyces cerevisiae* |
| C8ZAH8 | EC1118_1I12_1189p | *Saccharomyces cerevisiae* |
| C8ZF51 | EC1118_1M3_3796p | *Saccharomyces cerevisiae* |
| C8ZHE1 | EC1118_1O4_6480p | *Saccharomyces cerevisiae* |
| C8ZHF0 | EC1118_1O4_6579p | *Saccharomyces cerevisiae* |
| Q03E11 | Dihydrofolate reductase | *Pediococcus pentosaceus* |
| P53218 | Ribonucleases P/MRP protein subunit POP6 (EC 3.1.26.5) | *Saccharomyces cerevisiae* |
| D3W8P6 | Truncated aquaporin | *Saccharomyces cerevisiae* |
| P0CE68 | ABC transporter NFT1 (New full-length MRP-type transporter 1) | *Saccharomyces cerevisiae* |
| P37020 | Anion/proton exchange transporter GEF1 (CLC protein GEF1) (ClC-A) (ClC-Y1) (Voltage-gated chloride channel) [Cleaved into: GEF1 N-terminal; GEF1 C-terminal] | *Saccharomyces cerevisiae* |
| P40825 | Alanine--tRNA ligase, mitochondrial (EC 6.1.1.7) (Alanyl-tRNA synthetase) (AlaRS) | *Saccharomyces cerevisiae* |
| P38266 | Altered inheritance of mitochondria protein 3 | *Saccharomyces cerevisiae* |
| P31374 | Serine/threonine-protein kinase PSK1 (EC 2.7.11.1) (PAS kinase 1) | *Saccharomyces cerevisiae* |
| P38968 | Protein transport protein SEC31 (Protein WEB1) | *Saccharomyces cerevisiae* |
| G2HKE5 | K7_Bi4p | *Saccharomyces cerevisiae* |
| P53046 | RHO1 GDP-GTP exchange protein 1 (Protein kinase C suppressor SKC1) | *Saccharomyces cerevisiae* |
| P53327 | Antiviral helicase SLH1 (EC 3.6.4.13) (SKI2-like helicase 1) | *Saccharomyces cerevisiae* |
| Q01163 | 37S ribosomal protein S23, mitochondrial (DAP-3) (Mitochondrial small ribosomal subunit protein mS29) | *Saccharomyces cerevisiae* |
| G2WCK6 | K7_Yel025cp | *Saccharomyces cerevisiae* |
| G2WFY8 | K7_03404p | *Saccharomyces cerevisiae* |
| P38348 | DNA mismatch repair protein HSM3 (Enhanced spontaneous mutability protein 3) | *Saccharomyces cerevisiae* |
| P32565 | 26S proteasome regulatory subunit RPN2 | *Saccharomyces cerevisiae* |
| V5J299 | Glucoamylase A (Fragment) | *Rhizopus oryzae* |
| A0A0R2H8X7 | 50S ribosomal protein L34 | *Pediococcus pentosaceus* |
| A0A0R2L5Y2 | 50S ribosomal protein L34 | *Pediococcus stilesii* |
| A0A1A5VRE4 | 50S ribosomal protein L34 | *Pediococcus acidilactici* |
| D2EH17 | 50S ribosomal protein L34 | *Pediococcus acidilactici* |
| E0NG94 | 50S ribosomal protein L34 | *Pediococcus acidilactici* |
| Q03D56 | 50S ribosomal protein L34 | *Pediococcus pentosaceus* |
| P95540 | Replication protein | *Pediococcus pentosaceus* |
| P08518 | DNA-directed RNA polymerase II subunit RPB2 (RNA polymerase II subunit 2) (EC 2.7.7.6) | *Saccharomyces cerevisiae* |
| P57743 | U6 snRNA-associated Sm-like protein LSm3 (SmX4 protein) | *Saccharomyces cerevisiae* |
| A0A023IRJ6 | Alkane-inducible cytochrome P450 52A3 (EC 1.14.14.-) | *Saccharomyces cerevisiae* |
| P20486 | Cyclin-dependent kinases regulatory subunit (Cell division control protein CKS1) | *Saccharomyces cerevisiae* |
| Q05123 | Actin-like protein ARP9 (Chromatin structure-remodeling complex protein ARP9) | *Saccharomyces cerevisiae* |
| Q12151 | Sterol uptake control protein 2 (Mannoprotein regulation by oxygen protein 4) | *Saccharomyces cerevisiae* |
| Q03338 | U4/U6 small nuclear ribonucleoprotein PRP3 (Pre-mRNA-splicing factor 3) | *Saccharomyces cerevisiae* |
| A0A1P8L1E1 | Alpha-amylase (EC 3.2.1.1) | *Rhizopus oryzae* |
| P11978 | Regulatory protein SIR4 (Silent information regulator 4) | *Saccharomyces cerevisiae* |
| P31115 | tRNA pseudouridine(38/39) synthase (EC 5.4.99.45) | *Saccharomyces cerevisiae* |
| P53115 | Chromatin-remodeling ATPase INO80 (EC 3.6.4.-) (Inositol-requiring protein 80) | *Saccharomyces cerevisiae* |
| P10964 | DNA-directed RNA polymerase I subunit RPA190 (EC 2.7.7.6) | *Saccharomyces cerevisiae* |
| P47075 | Vacuolar transporter chaperone 4 (Phosphate metabolism protein 3) | *Saccharomyces cerevisiae* |
| Q07834 | KH domain-containing protein YLL032C | *Saccharomyces cerevisiae* |
| P06778 | DNA repair and recombination protein RAD52 | *Saccharomyces cerevisiae* |
| Q03DZ7 | L-lactate dehydrogenase (L-LDH) (EC 1.1.1.27) | *Pediococcus pentosaceus* |
| G2WPT4 | K7_Ypr117wp | *Saccharomyces cerevisiae* |
| A0A1Y0VPZ8 | N,N'-diacetylchitobiose permease IIC component | *Pediococcus pentosaceus* |
| P28007 | H/ACA ribonucleoprotein complex subunit GAR1 (snoRNP protein GAR1) | *Saccharomyces cerevisiae* |
| P24000 | 60S ribosomal protein L24-B (L30) (Large ribosomal subunit protein eL24-B) (RP29) (YL21) | *Saccharomyces cerevisiae* |
| P32892 | ATP-dependent RNA helicase DRS1 (EC 3.6.4.13) (Deficiency of ribosomal subunits protein 1) | *Saccharomyces cerevisiae* |
| P17121 | GTPase-activating protein SAC7 | *Saccharomyces cerevisiae* |
| P32368 | Phosphoinositide phosphatase SAC1 (EC 3.1.3.-) (Recessive suppressor of secretory defect) | *Saccharomyces cerevisiae* |
| P34163 | Sterol esterase TGL1 (EC 3.1.1.13) | *Saccharomyces cerevisiae* |
| A2NY36 | ORF 107 protein | *Saccharomyces cerevisiae* |
| P08458 | Sporulation-specific protein 1 (EC 2.7.11.1) | *Saccharomyces cerevisiae* |
| P32074 | Coatomer subunit gamma (Gamma-coat protein) (Gamma-COP) | *Saccharomyces cerevisiae* |
| P53978 | Elongation factor 3B (EF-3B) (Homolog of EF-3) | *Saccharomyces cerevisiae* |
| P20449 | ATP-dependent RNA helicase DBP5 (EC 3.6.4.13) | *Saccharomyces cerevisiae* |
| P32493 | ATPase expression protein 1, mitochondrial | *Saccharomyces cerevisiae* |
| P43132 | COMPASS component BRE2 (Brefeldin-A sensitivity protein 2) | *Saccharomyces cerevisiae* |
| O13513 | Putative uncharacterized protein YAL056C-A | *Saccharomyces cerevisiae* |
| Q08925 | RNA-binding protein MRN1 (Multicopy suppressor of RSC-NHP6 synthetic lethality protein 1) | *Saccharomyces cerevisiae* |
| P40328 | Probable 26S proteasome subunit YTA6 (Tat-binding homolog 6) | *Saccharomyces cerevisiae* |
| Q12192 | Repression factor of MSEs protein 1 | *Saccharomyces cerevisiae* |
| Q04921 | Sporulation-regulated protein 28 | *Saccharomyces cerevisiae* |
| Q12504 | Ribosomal lysine N-methyltransferase 4 (EC 2.1.1.-) (SET domain-containing protein 7) | *Saccharomyces cerevisiae* |
| Q08811 | Putative uncharacterized protein YOR345C | *Saccharomyces cerevisiae* |
| Q06671 | Autophagy-related protein 23 (Cytoplasm to vacuole targeting protein 23) | *Saccharomyces cerevisiae* |
| Q08421 | Enhancer of translation termination 1 | *Saccharomyces cerevisiae* |
| Q12029 | Sideroflexin FSF1 (Fungal sideroflexin-1) | *Saccharomyces cerevisiae* |
| Q03GZ8 | Protein translocase subunit SecA 1 | *Pediococcus pentosaceus* |
| P41930 | Sulfite efflux pump SSU1 (Sulfite sensitivity protein SSU1) | *Saccharomyces cerevisiae* |
| B5VGX4 | Uncharacterized protein | *Saccharomyces cerevisiae* |
| B5VEE1 | YBR191Wp-like protein | *Saccharomyces cerevisiae* |
| C7GWD3 | Tre2p | *Saccharomyces cerevisiae* |
| C8ZJK6 | EC1118_1P2_5226p | *Saccharomyces cerevisiae* |
| P0CX42 | 60S ribosomal protein L23-B (L17a) (Large ribosomal subunit protein uL14-B) (YL32) | *Saccharomyces cerevisiae* |
| G2WD82 | K7_02212p | *Saccharomyces cerevisiae* |
| A0A0A1H265 | ArsR family transcriptional regulator | *Lactobacillus hokkaidonensis* |
| A0A0E2BLS0 | Arsenical resistance operon repressor | *Lactobacillus casei A2-362* |
| A0A1J3C9G9 | ArsR family transcriptional regulator (Arsenical resistance operon repressor) (Transcriptional repressor SdpR) | *Lactobacillus paracasei* |
| A0A1X1FB73 | Transcriptional repressor SdpR | *Lactobacillus parabuchneri* |
| P02309 | Histone H4 | *Saccharomyces cerevisiae* |
| P46674 | Nuclear mRNA export protein SAC3 (Leucine permease transcriptional regulator) | *Saccharomyces cerevisiae* |
| P26309 | APC/C activator protein CDC20 (Cell division control protein 20) | *Saccharomyces cerevisiae* |
| P28004 | Pre-mRNA-processing protein 45 | *Saccharomyces cerevisiae* |
| P28003 | SWIRM domain-containing protein FUN19 | *Saccharomyces cerevisiae* |
| P27697 | Atypical kinase COQ8, mitochondrial (EC 2.7.-.-) (Activity of bc1 complex protein 1) (Coenzyme Q protein 8) (Ubiquinone biosynthesis protein COQ8) | *Saccharomyces cerevisiae* |
| P00331 | Alcohol dehydrogenase 2 (EC 1.1.1.1) (Alcohol dehydrogenase II) (YADH-2) | *Saccharomyces cerevisiae* |
| P04710 | ADP,ATP carrier protein 1 (ADP/ATP translocase 1) (Adenine nucleotide translocator 1) (ANT 1) | *Saccharomyces cerevisiae* |
| P07250 | Inositol polyphosphate multikinase (IPMK) (EC 2.7.1.151) (Arginine metabolism regulation protein III) | *Saccharomyces cerevisiae* |
| P00812 | Arginase (EC 3.5.3.1) | *Saccharomyces cerevisiae* |
| P28777 | Chorismate synthase (EC 4.2.3.5) (5-enolpyruvylshikimate-3-phosphate phospholyase) | *Saccharomyces cerevisiae* |
| P15703 | Glucan 1,3-beta-glucosidase (EC 3.2.1.58) (Exo-1,3-beta-glucanase) (GP29) (Soluble cell wall protein 9) | *Saccharomyces cerevisiae* |
| P27825 | Calnexin homolog | *Saccharomyces cerevisiae* |
| P13365 | G1/S-specific cyclin CLN3 | *Saccharomyces cerevisiae* |
| P24867 | PHO85 cyclin-1 (Cyclin HCS26) (G1/S-specific cyclin PCL1) | *Saccharomyces cerevisiae* |
| P25337 | Pre-mRNA-splicing factor BUD31 (Bud site selection protein 31) (Complexed with CEF1 protein 14) | *Saccharomyces cerevisiae* |
| P02293 | Histone H2B.1 (Suppressor of Ty protein 12) | *Saccharomyces cerevisiae* |
| P09950 | 5-aminolevulinate synthase, mitochondrial (EC 2.3.1.37) (5-aminolevulinic acid synthase) (Delta-ALA synthase) (Delta-aminolevulinate synthase) | *Saccharomyces cerevisiae* |
| P12684 | 3-hydroxy-3-methylglutaryl-coenzyme A reductase 2 (HMG-CoA reductase 2) (EC 1.1.1.34) | *Saccharomyces cerevisiae* |
| P02829 | ATP-dependent molecular chaperone HSP82 (82 kDa heat shock protein) (Heat shock protein Hsp90 heat-inducible isoform) | *Saccharomyces cerevisiae* |
| P23585 | High-affinity glucose transporter HXT2 | *Saccharomyces cerevisiae* |
| P01097 | ATPase inhibitor, mitochondrial (ATP synthase F1 subunit epsilon) | *Saccharomyces cerevisiae* |
| P26364 | GTP:AMP phosphotransferase, mitochondrial (EC 2.7.4.10) (Adenylate kinase 3) (AK 3) | *Saccharomyces cerevisiae* |
| P06242 | Serine/threonine-protein kinase KIN28 (EC 2.7.11.23) | *Saccharomyces cerevisiae* |
| P25491 | Mitochondrial protein import protein MAS5 (Yeast dnaJ protein 1) | *Saccharomyces cerevisiae* |
| P23059 | N-alpha-acetyltransferase 38, NatC auxiliary subunit (L-A virus GAG protein N-acetyltransferase subunit MAK31) (Maintenance of killer protein 31) (N-terminal acetyltransferase C complex subunit MAK31) (NatC complex subunit MAK31) | *Saccharomyces cerevisiae* |
| P27476 | Nuclear localization sequence-binding protein (p67) | *Saccharomyces cerevisiae* |
| P05374 | Phosphatidylethanolamine N-methyltransferase (PE methyltransferase) (PEAMT) (PEMT) (EC 2.1.1.17) (Choline-requiring protein 2) | *Saccharomyces cerevisiae* |
| P24384 | Pre-mRNA-splicing factor ATP-dependent RNA helicase PRP22 (EC 3.6.4.13) | *Saccharomyces cerevisiae* |
| P12753 | DNA repair protein RAD50 (EC 3.6.-.-) (153 kDa protein) | *Saccharomyces cerevisiae* |
| P03872 | Partitioning protein REP2 (R2) (Protein Charlie) (Trans-acting factor C) | *Saccharomyces cerevisiae* |
| P17076 | 60S ribosomal protein L8-A (L4) (L4-2) (L7a-1) (Large ribosomal subunit protein eL8-A) (Maintenance of killer protein 7) (RP6) (YL5) | *Saccharomyces cerevisiae* |
| P11745 | Ran GTPase-activating protein 1 (Protein involved in RNA production/processing) | *Saccharomyces cerevisiae* |
| P23293 | Serine/threonine-protein kinase BUR1 (EC 2.7.11.22) (EC 2.7.11.23) (Bypass UAS requirement protein 1) (Suppressor of GPA1-Vall50 mutation protein 1) | *Saccharomyces cerevisiae* |
| P06700 | NAD-dependent histone deacetylase SIR2 (EC 2.3.1.286) (Regulatory protein SIR2) (Silent information regulator 2) | *Saccharomyces cerevisiae* |
| P25294 | Protein SIS1 | *Saccharomyces cerevisiae* |
| P22082 | Transcription regulatory protein SNF2 (EC 3.6.4.-) (ATP-dependent helicase SNF2) (Regulatory protein GAM1) (Regulatory protein SWI2) (SWI/SNF complex component SNF2) (Transcription factor TYE3) | *Saccharomyces cerevisiae* |
| P00445 | Superoxide dismutase [Cu-Zn] (EC 1.15.1.1) | *Saccharomyces cerevisiae* |
| P23615 | Transcription elongation factor SPT6 (Chromatin elongation factor SPT6) | *Saccharomyces cerevisiae* |
| P06784 | Serine/threonine-protein kinase STE7 (EC 2.7.12.2) | *Saccharomyces cerevisiae* |
| P09436 | Isoleucine--tRNA ligase, cytoplasmic (EC 6.1.1.5) (Isoleucyl-tRNA synthetase) (IleRS) | *Saccharomyces cerevisiae* |
| P16649 | General transcriptional corepressor TUP1 (Flocculation suppressor protein) (Glucose repression regulatory protein TUP1) (Repressor AER2) | *Saccharomyces cerevisiae* |
| P12887 | Uracil-DNA glycosylase (UDG) (EC 3.2.2.27) | *Saccharomyces cerevisiae* |
| P25594 | Vacuolar basic amino acid transporter 3 | *Saccharomyces cerevisiae* |
| P23292 | Casein kinase I homolog 2 (EC 2.7.11.1) | *Saccharomyces cerevisiae* |
| P25348 | 54S ribosomal protein L32, mitochondrial (Mitochondrial large ribosomal subunit protein bL32m) (YmL32) | *Saccharomyces cerevisiae* |
| P25627 | 18S rRNA (guanine(1575)-N(7))-methyltransferase (EC 2.1.1.309) (Bud site selection protein 23) | *Saccharomyces cerevisiae* |
| P25365 | Putative guanine nucleotide-exchange factor SED4 | *Saccharomyces cerevisiae* |
| P07251 | ATP synthase subunit alpha, mitochondrial | *Saccharomyces cerevisiae* |
| P32452 | Putative prephenate dehydratase (PDT) (EC 4.2.1.51) (Phenylalanine-requiring protein 2) | *Saccharomyces cerevisiae* |
| P25367 | [PIN+] prion protein RNQ1 (Rich in asparagine and glutamine protein 1) | *Saccharomyces cerevisiae* |
| P23503 | Cytosolic Fe-S cluster assembly factor NAR1 (Nuclear architecture-related protein 1) | *Saccharomyces cerevisiae* |
| Q00711 | Succinate dehydrogenase [ubiquinone] flavoprotein subunit, mitochondrial (EC 1.3.5.1) (Flavoprotein subunit of complex II) (FP) | *Saccharomyces cerevisiae* |
| Q00916 | U1 small nuclear ribonucleoprotein 70 kDa homolog (U1 70K) (U1 snRNP 70 kDa homolog) (U1-70K) (U1 small nuclear ribonucleoprotein SNP1) (U1 snRNP protein SNP1) | *Saccharomyces cerevisiae* |
| P29478 | Signal recognition particle subunit SEC65 | *Saccharomyces cerevisiae* |
| P30619 | Protein transport protein SEC1 | *Saccharomyces cerevisiae* |
| P30902 | ATP synthase subunit d, mitochondrial | *Saccharomyces cerevisiae* |
| Q02207 | Peroxisomal hydratase-dehydrogenase-epimerase (HDE) (Multifunctional beta-oxidation protein) (MFP) [Includes: 2-enoyl-CoA hydratase (EC 4.2.1.119); (3R)-3-hydroxyacyl-CoA dehydrogenase (EC 1.1.1.n12)] | *Saccharomyces cerevisiae* |
| P30952 | Malate synthase 1 (EC 2.3.3.9) | *Saccharomyces cerevisiae* |
| Q01560 | Nucleolar protein 3 (Mitochondrial targeting suppressor 1 protein) (Nuclear polyadenylated RNA-binding protein 1) | *Saccharomyces cerevisiae* |
| P21576 | Vacuolar protein sorting-associated protein 1 | *Saccharomyces cerevisiae* |
| P31380 | ATP-dependent helicase FUN30 (EC 3.6.4.12) | *Saccharomyces cerevisiae* |
| P24813 | AP-1-like transcription factor YAP2 (Cadmium resistance protein 1) (Transcription factor CAD1) | *Saccharomyces cerevisiae* |
| P32872 | Aldehyde dehydrogenase 2, mitochondrial (EC 1.2.1.3) | *Saccharomyces cerevisiae* |
| P32462 | Delta(14)-sterol reductase (EC 1.3.1.70) (C-14 sterol reductase) (Sterol C14-reductase) | *Saccharomyces cerevisiae* |
| P32353 | Delta(7)-sterol 5(6)-desaturase (EC 1.14.19.20) (C-5 sterol desaturase) (Ergosterol Delta(5,6) desaturase) (Sterol-C5-desaturase) | *Saccharomyces cerevisiae* |
| P32501 | Translation initiation factor eIF-2B subunit epsilon (GCD complex subunit GCD6) (Guanine nucleotide exchange factor subunit GCD6) (eIF-2B GDP-GTP exchange factor subunit epsilon) | *Saccharomyces cerevisiae* |
| P32190 | Glycerol kinase (GK) (Glycerokinase) (EC 2.7.1.30) (ATP:glycerol 3-phosphotransferase) | *Saccharomyces cerevisiae* |
| P09435 | Heat shock protein SSA3 | *Saccharomyces cerevisiae* |
| P32787 | Mitochondrial genome maintenance protein MGM101 | *Saccharomyces cerevisiae* |
| P32445 | Single-stranded DNA-binding protein RIM1, mitochondrial (Mitochondrial ssDNA-binding protein) | *Saccharomyces cerevisiae* |
| P32605 | U1 small nuclear ribonucleoprotein A (U1 snRNP A) (U1-A) (U1A) (Mutant U1 die protein 1) | *Saccharomyces cerevisiae* |
| P10080 | Single-stranded nucleic acid-binding protein | *Saccharomyces cerevisiae* |
| P32591 | SWI/SNF complex subunit SWI3 (Transcription factor TYE2) (Transcription regulatory protein SWI3) | *Saccharomyces cerevisiae* |
| P32862 | Glucose transport transcription regulator RGT1 (Restores glucose transport protein 1) | *Saccharomyces cerevisiae* |
| V9H070 | LAP16 | *Saccharomyces cerevisiae* |
| P34164 | SNF1 protein kinase subunit beta-2 (Protein SPM2) (SNF1-interacting protein 2) | *Saccharomyces cerevisiae* |
| P33312 | 2,5-diamino-6-ribosylamino-4(3H)-pyrimidinone 5'-phosphate reductase (DAROPP reductase) (DARP reductase) (EC 1.1.1.302) (2,5-diamino-6-(5-phospho-D-ribosylamino)pyrimidin-4(3H)-one reductase) (2,5-diamino-6-ribitylamino-4(3H)-pyrimidinone 5'-phosphate synthase) (DARIPP synthase) | *Saccharomyces cerevisiae* |
| P33313 | Hsp70/Hsp90 co-chaperone CNS1 (Cyclophilin seven suppressor 1) (STI1 stress-inducible protein homolog) | *Saccharomyces cerevisiae* |
| P34230 | Peroxisomal long-chain fatty acid import protein 1 (Peroxisomal ABC transporter 2) | *Saccharomyces cerevisiae* |
| P33338 | Protein SLA2 (Transmembrane protein MOP2) | *Saccharomyces cerevisiae* |
| P40990 | Protein MSS2, mitochondrial | *Saccharomyces cerevisiae* |
| P36100 | Transcription initiation factor IIE subunit alpha (TFIIE-alpha) | *Saccharomyces cerevisiae* |
| P36149 | Trafficking protein particle complex subunit BET3 (TRAPP subunit BET3) | *Saccharomyces cerevisiae* |
| P36165 | Lipase 4 (EC 3.1.1.3) (Triacylglycerol lipase 4) | *Saccharomyces cerevisiae* |
| P36102 | PAN2-PAN3 deadenylation complex subunit PAN3 (PAB1P-dependent poly(A)-specific ribonuclease) | *Saccharomyces cerevisiae* |
| P36096 | Transmembrane E3 ubiquitin-protein ligase 1 (EC 2.3.2.27) | *Saccharomyces cerevisiae* |
| P32860 | NifU-like protein, mitochondrial | *Saccharomyces cerevisiae* |
| P35731 | 3-oxoacyl-[acyl-carrier-protein] reductase (EC 1.1.1.100) | *Saccharomyces cerevisiae* |
| P36076 | Coenzyme A biosynthesis protein 3 | *Saccharomyces cerevisiae* |
| P32343 | Protein SSH4 (Multicopy suppressor of leflunomide protein 4) | *Saccharomyces cerevisiae* |
| P36049 | rRNA-processing protein EBP2 (EBNA1-binding protein homolog) | *Saccharomyces cerevisiae* |
| P36035 | Carboxylic acid transporter protein homolog | *Saccharomyces cerevisiae* |
| P36033 | Ferric/cupric reductase transmembrane component 2 (EC 1.16.1.9) (Ferric-chelate reductase 2) | *Saccharomyces cerevisiae* |
| P18239 | ADP,ATP carrier protein 2 (ADP/ATP translocase 2) ) | *Saccharomyces cerevisiae* |
| P38630 | Replication factor C subunit 1 (Replication factor C1) | *Saccharomyces cerevisiae* |
| P38431 | Eukaryotic translation initiation factor 5 (eIF-5) | *Saccharomyces cerevisiae* |
| P00635 | Repressible acid phosphatase (EC 3.1.3.2) (P60) | *Saccharomyces cerevisiae* |
| P21538 | DNA-binding protein REB1 (QBP) | *Saccharomyces cerevisiae* |
| P38064 | 54S ribosomal protein L16, mitochondrial (Mitochondrial large ribosomal subunit protein uL16m) | *Saccharomyces cerevisiae* |
| P38427 | Trehalose synthase complex regulatory subunit TSL1 (Alpha,alpha-trehalose-phosphate synthase [UDP-forming] 123 kDa subunit) | *Saccharomyces cerevisiae* |
| P38292 | Peroxisomal membrane protein PEX32 (Peroxin-32) | *Saccharomyces cerevisiae* |
| P38335 | Maintenance of telomere capping protein 4 | *Saccharomyces cerevisiae* |
| P38181 | Nucleoporin NUP170 (Nuclear pore protein NUP170) | *Saccharomyces cerevisiae* |
| P38230 | Probable quinone oxidoreductase (EC 1.6.5.5) (NADPH:quinone reductase) | *Saccharomyces cerevisiae* |
| P38232 | Protein REG2 | *Saccharomyces cerevisiae* |
| P38249 | Eukaryotic translation initiation factor 3 subunit A (eIF3a) (Eukaryotic translation initiation factor 3 110 kDa subunit homolog) (eIF3 p110) (Translation initiation factor eIF3, p110 subunit homolog) | *Saccharomyces cerevisiae* |
| P38112 | ATP-dependent RNA helicase MAK5 (EC 3.6.4.13) (Maintenance of killer protein 5) | *Saccharomyces cerevisiae* |
| P04819 | DNA ligase 1 (EC 6.5.1.1) (DNA ligase I) (Polydeoxyribonucleotide synthase [ATP] 1) | *Saccharomyces cerevisiae* |
| P40000 | Putative uncharacterized protein YEL010W | *Saccharomyces cerevisiae* |
| P40062 | Putative uncharacterized protein YER097W | *Saccharomyces cerevisiae* |
| P40103 | Uncharacterized protein YER188W | *Saccharomyces cerevisiae* |
| P38696 | Centractin (Actin-like protein) (Actin-related protein 1) | *Saccharomyces cerevisiae* |
| P40994 | ADP-ribosylation factor 3 | *Saccharomyces cerevisiae* |
| P38986 | L-asparaginase 1 (EC 3.5.1.1) | *Saccharomyces cerevisiae* |
| P38929 | Calcium-transporting ATPase 2 (EC 7.2.2.10) | *Saccharomyces cerevisiae* |
| P40987 | Chromosome instability protein 1 | *Saccharomyces cerevisiae* |
| P21373 | NAD(+) kinase (EC 2.7.1.23) (Unknown transcript 1 protein) | *Saccharomyces cerevisiae* |
| P38682 | ADP-ribosylation factor GTPase-activating protein GLO3 (ARF GAP GLO3) | *Saccharomyces cerevisiae* |
| P32191 | Glycerol-3-phosphate dehydrogenase, mitochondrial (GPD-M) (GPDH-M) (EC 1.1.5.3) | *Saccharomyces cerevisiae* |
| P40150 | Ribosome-associated molecular chaperone SSB2 (EC 3.6.4.10) (Heat shock protein SSB2) | *Saccharomyces cerevisiae* |
| P38695 | Probable glucose transporter HXT5 | *Saccharomyces cerevisiae* |
| P40970 | Serine palmitoyltransferase 2 (SPT 2) (EC 2.3.1.50) (Long chain base biosynthesis protein 2) | *Saccharomyces cerevisiae* |
| P20967 | 2-oxoglutarate dehydrogenase, mitochondrial (EC 1.2.4.2) (2-oxoglutarate dehydrogenase complex component E1) (OGDC-E1) (Alpha-ketoglutarate dehydrogenase) | *Saccharomyces cerevisiae* |
| P16387 | Pyruvate dehydrogenase E1 component subunit alpha, mitochondrial (EC 1.2.4.1) (Pyruvate dehydrogenase complex component E1 alpha) (PDHE1-A) | *Saccharomyces cerevisiae* |
| P39104 | Phosphatidylinositol 4-kinase PIK1 (PI4-kinase) (PtdIns-4-kinase) (EC 2.7.1.67) | *Saccharomyces cerevisiae* |
| P39682 | Pre-mRNA-processing factor 39 | *Saccharomyces cerevisiae* |
| P40352 | DNA repair and recombination protein RAD26 (EC 3.6.4.12) | *Saccharomyces cerevisiae* |
| Q00245 | GTP-binding protein RHO3 | *Saccharomyces cerevisiae* |
| P40693 | Ribosome biogenesis protein RLP7 (Ribosomal protein L7-like) | *Saccharomyces cerevisiae* |
| P39940 | E3 ubiquitin-protein ligase RSP5 (EC 2.3.2.26) | *Saccharomyces cerevisiae* |
| P38889 | Transcription factor SKN7 (Peroxide sensitivity protein 9) | *Saccharomyces cerevisiae* |
| P38985 | Signal recognition particle subunit SRP14 (Signal recognition particle 14 kDa protein homolog) | *Saccharomyces cerevisiae* |
| P40327 | 26S proteasome regulatory subunit 4 homolog (Tat-binding homolog 5) | *Saccharomyces cerevisiae* |
| P18411 | Protein FUN14 | *Saccharomyces cerevisiae* |
| P39706 | COMPASS component SWD1 (Complex proteins associated with SET1 protein SWD1) (Set1C component SWD1) | *Saccharomyces cerevisiae* |
| P40098 | Uncharacterized mitochondrial membrane protein FMP10 | *Saccharomyces cerevisiae* |
| P39953 | Mitochondrial nicotinamide adenine dinucleotide transporter 2 (Mitochondrial NAD(+) transporter 2) | *Saccharomyces cerevisiae* |
| P39987 | Heat shock protein SSC3, mitochondrial (Extracellular mutant protein 10) | *Saccharomyces cerevisiae* |
| P39976 | D-2-hydroxyglutarate--pyruvate transhydrogenase DLD3 (D-2HG--pyruvate transhydrogenase DLD3) (EC 1.1.99.40) ((R)-2-hydroxyglutarate--pyruvate transhydrogenase) (D-lactate dehydrogenase [cytochrome] 3) (EC 1.1.2.4) (D-lactate ferricytochrome C oxidoreductase) (D-LCR) | *Saccharomyces cerevisiae* |
| P40017 | Carnitine O-acetyltransferase YAT2 (EC 2.3.1.7) | *Saccharomyces cerevisiae* |
| P40051 | Intermediate cleaving peptidase 55 (EC 3.4.11.26) | *Saccharomyces cerevisiae* |
| P39000 | Protein SHC1 (Sporulation-specific homolog of CSD4) | *Saccharomyces cerevisiae* |
| P40088 | Plasma membrane iron permease | *Saccharomyces cerevisiae* |
| P38744 | Putative pterin-4-alpha-carbinolamine dehydratase (PHS) (EC 4.2.1.96) | *Saccharomyces cerevisiae* |
| P38787 | 2-dehydropantoate 2-reductase (EC 1.1.1.169) (Ketopantoate reductase) | *Saccharomyces cerevisiae* |
| P38796 | Protein phosphatase methylesterase 1 (PME-1) (EC 3.1.1.89) (Yms2) | *Saccharomyces cerevisiae* |
| P38715 | NADPH-dependent aldose reductase GRE3 (AR) (EC 1.1.1.21) (Genes de respuesta a estres protein 3) (NADPH-dependent aldo-keto reductase GRE3) (Xylose reductase) (EC 1.1.1.-) | *Saccharomyces cerevisiae* |
| P38817 | ADP-ribosylation factor-binding protein GGA2 (Golgi-localized, gamma ear-containing, ARF-binding protein 2) | *Saccharomyces cerevisiae* |
| P38827 | Histone-lysine N-methyltransferase, H3 lysine-4 specific (EC 2.1.1.354) (COMPASS component SET1) (Lysine N-methyltransferase 2) (SET domain-containing protein 1) | *Saccharomyces cerevisiae* |
| P38853 | Kelch repeat-containing protein 1 | *Saccharomyces cerevisiae* |
| P38876 | Peptidyl-tRNA hydrolase (PTH) (EC 3.1.1.29) | *Saccharomyces cerevisiae* |
| P40555 | Probable 26S proteasome regulatory subunit p27 (Proteasome non-ATPase subunit 2) | *Saccharomyces cerevisiae* |
| P40550 | ATP-dependent permease PDR11 | *Saccharomyces cerevisiae* |
| P40546 | Protein FAF1 (Forty S assembly factor) | *Saccharomyces cerevisiae* |
| P40516 | Protein-lysine N-methyltransferase EFM4 (EC 2.1.1.-) (Elongation factor methyltransferase 4) (Secretion and early endocytosis protein 1) | *Saccharomyces cerevisiae* |
| P40504 | Probable mannosyltransferase KTR7 (EC 2.4.1.-) | *Saccharomyces cerevisiae* |
| P40495 | Homoisocitrate dehydrogenase, mitochondrial (HIcDH) (EC 1.1.1.87) | *Saccharomyces cerevisiae* |
| P40492 | Protein FYV10 (EC 2.3.2.27) | *Saccharomyces cerevisiae* |
| P40482 | Protein transport protein SEC24 (Abnormal nuclear morphology 1) | *Saccharomyces cerevisiae* |
| P40474 | Quinidine resistance protein 2 | *Saccharomyces cerevisiae* |
| P40447 | Putative nitrilase-like protein NIT1 | *Saccharomyces cerevisiae* |
| P40360 | Amino-acid acetyltransferase, mitochondrial (EC 2.3.1.1) (Arginine-requiring protein 2) (Glutamate N-acetyltransferase) (N-acetylglutamate synthase) | *Saccharomyces cerevisiae* |
| P32525 | Protein ECM25 (Extracellular matrix protein 25) | *Saccharomyces cerevisiae* |
| P39542 | Uncharacterized transporter YJL193W | *Saccharomyces cerevisiae* |
| P40890 | VPS10 homolog 2 (Sortilin VTH2) | *Saccharomyces cerevisiae* |
| P40164 | Serine/threonine-protein phosphatase 4 regulatory subunit 3 (PP4R3) | *Saccharomyces cerevisiae* |
| A2NY33 | ORF 115 protein | *Saccharomyces cerevisiae* |
| E9PA89 | Orf protein | *Saccharomyces cerevisiae* |
| P10963 | Phosphoenolpyruvate carboxykinase (ATP) (EC 4.1.1.49) | *Saccharomyces cerevisiae* |
| Q12324 | Calcium channel YVC1 (TRP homolog) (Yeast vacuolar conductance protein 1) | *Saccharomyces cerevisiae* |
| P46681 | D-2-hydroxyglutarate--pyruvate transhydrogenase DLD2 (D-2HG--pyruvate transhydrogenase DLD2) (EC 1.1.99.40) (Actin-interacting protein 2) (D-lactate dehydrogenase [cytochrome] 2, mitochondrial) (EC 1.1.2.4) (D-lactate ferricytochrome C oxidoreductase) (D-LCR) | *Saccharomyces cerevisiae* |
| P07834 | Cell division control protein 4 (E3 ubiquitin ligase complex SCF subunit CDC4) (F-box protein CDC4) | *Saccharomyces cerevisiae* |
| P06243 | Cell division control protein 7 (EC 2.7.11.1) | *Saccharomyces cerevisiae* |
| P00360 | Glyceraldehyde-3-phosphate dehydrogenase 1 (GAPDH 1) (EC 1.2.1.12) | *Saccharomyces cerevisiae* |
| P43535 | Protein GCN20 (General control non-derepressible protein 20) | *Saccharomyces cerevisiae* |
| P20448 | ATP-dependent RNA helicase HCA4 (EC 3.6.4.13) (DEAD box protein 4) (Helicase CA4) (Helicase UF1) | *Saccharomyces cerevisiae* |
| P43565 | Serine/threonine-protein kinase RIM15 (EC 2.7.11.1) | *Saccharomyces cerevisiae* |
| P46654 | 40S ribosomal protein S0-B (Nucleic acid-binding protein NAB1B) (Small ribosomal subunit protein uS2-B) | *Saccharomyces cerevisiae* |
| P34077 | Nucleoporin NIC96 (96 kDa nucleoporin-interacting component) (Nuclear pore protein NIC96) | *Saccharomyces cerevisiae* |
| P38798 | Nonsense-mediated mRNA decay protein 2 (Up-frameshift suppressor 2) | *Saccharomyces cerevisiae* |
| P39684 | Protein PES4 (DNA polymerase epsilon suppressor 4) | *Saccharomyces cerevisiae* |
| P43122 | tRNA N6-adenosine threonylcarbamoyltransferase, mitochondrial (EC 2.3.1.234) (N6-L-threonylcarbamoyladenine synthase) (t(6)A synthase) (t(6)A37 threonylcarbamoyladenosine biosynthesis protein QRI7) (tRNA threonylcarbamoyladenosine biosynthesis protein QRI7) | *Saccharomyces cerevisiae* |
| P05750 | 40S ribosomal protein S3 (RP13) (Small ribosomal subunit protein uS3) (YS3) | *Saccharomyces cerevisiae* |
| P41901 | Sporulation-regulated protein 3 | *Saccharomyces cerevisiae* |
| P43558 | Ubiquitin thioesterase OTU1 (EC 3.4.19.12) (OTU domain-containing protein 1) | *Saccharomyces cerevisiae* |
| P43553 | Magnesium transporter ALR2 (Aluminum resistance protein 2) | *Saccharomyces cerevisiae* |
| P43613 | ER-localized J domain-containing protein 5 | *Saccharomyces cerevisiae* |
| P43620 | Sporulation protein RMD8 (Required for meiotic nuclear division protein 8) | *Saccharomyces cerevisiae* |
| P47137 | Uncharacterized oxidoreductase YJR096W (EC 1.-.-.-) | *Saccharomyces cerevisiae* |
| P42951 | Phosphatidylinositol 4-kinase LSB6 (PI4-kinase) (PtdIns-4-kinase) (EC 2.7.1.67) | *Saccharomyces cerevisiae* |
| P41903 | Peroxisomal acyl-coenzyme A thioester hydrolase 1 (EC 3.1.2.2) | *Saccharomyces cerevisiae* |
| P42842 | Essential for maintenance of the cell wall protein 1 | *Saccharomyces cerevisiae* |
| E9P982 | P-type ATPase | *Saccharomyces cerevisiae* |
| Q12459 | Pheromone-regulated protein PRM7 | *Saccharomyces cerevisiae* |
| E9PA87 | L2943 protein | *Saccharomyces cerevisiae* |
| P48524 | Ubiquitin ligase-binding protein BUL1 (Respiration deficiency suppressor 1) | *Saccharomyces cerevisiae* |
| P22146 | 1,3-beta-glucanosyltransferase GAS1 (EC 2.4.1.-) (Glycolipid-anchored surface protein 1) (Glycoprotein GP115) | *Saccharomyces cerevisiae* |
| P38631 | 1,3-beta-glucan synthase component FKS1 (EC 2.4.1.34) | *Saccharomyces cerevisiae* |
| P46961 | Phosphatidylinositol N-acetylglucosaminyltransferase GPI2 subunit (EC 2.4.1.198) | *Saccharomyces cerevisiae* |
| P40850 | Protein MKT1 | *Saccharomyces cerevisiae* |
| P25270 | rRNA methyltransferase 1, mitochondrial | *Saccharomyces cerevisiae* |
| P42073 | RNA end formation protein 2 | *Saccharomyces cerevisiae* |
| P48570 | Homocitrate synthase, cytosolic isozyme (HCS) (EC 2.3.3.14) | *Saccharomyces cerevisiae* |
| P07246 | Alcohol dehydrogenase 3, mitochondrial (EC 1.1.1.1) (Alcohol dehydrogenase III) (YADH-3) | *Saccharomyces cerevisiae* |
| P22580 | Probable amidase (EC 3.5.1.4) | *Saccharomyces cerevisiae* |
| P05085 | Arginine metabolism regulation protein II (Arginine-requiring protein 81) | *Saccharomyces cerevisiae* |
| P40416 | Iron-sulfur clusters transporter ATM1, mitochondrial | *Saccharomyces cerevisiae* |
| P47102 | ARF guanine-nucleotide exchange factor 1 | *Saccharomyces cerevisiae* |
| P47108 | Nucleolar pre-ribosomal-associated protein 2 (Unhealthy ribosome biogenesis protein 2) | *Saccharomyces cerevisiae* |
| P47112 | Cell division cycle protein CDT1 (SIC1 indispensable protein 2) (Topoisomerase-A hypersensitive protein 11) | *Saccharomyces cerevisiae* |
| P47127 | Altered inheritance of mitochondria protein 24, mitochondrial | *Saccharomyces cerevisiae* |
| P47166 | Protein SGM1 (Slow growth on galactose and mannose protein 1) | *Saccharomyces cerevisiae* |
| P47170 | Vacuolar membrane-associated protein IML1 (Increased minichromosome loss protein 1) (SEH-associated protein 1) | *Saccharomyces cerevisiae* |
| P47015 | Altered inheritance of mitochondria protein 23, mitochondrial | *Saccharomyces cerevisiae* |
| P47089 | Translation machinery-associated protein 22 | *Saccharomyces cerevisiae* |
| P52893 | Probable alanine aminotransferase, mitochondrial (EC 2.6.1.2) | *Saccharomyces cerevisiae* |
| P53629 | Sterol O-acyltransferase 2 (EC 2.3.1.26) (Sterol-ester synthase 2) | *Saccharomyces cerevisiae* |
| P49956 | Chromosome transmission fidelity protein 18 | *Saccharomyces cerevisiae* |
| P54861 | Dynamin-related protein DNM1 (EC 3.6.5.5) | *Saccharomyces cerevisiae* |
| P53037 | Phosphatidylserine decarboxylase proenzyme 2 (EC 4.1.1.65) | *Saccharomyces cerevisiae* |
| P32476 | Squalene monooxygenase (EC 1.14.14.17) | *Saccharomyces cerevisiae* |
| P06174 | Uroporphyrinogen-III synthase (UROIIIS) (UROS) (EC 4.2.1.75) | *Saccharomyces cerevisiae* |
| P41800 | Maintenance of mitochondrial morphology protein 1 (Mitochondrial outer membrane protein MMM1) (Yeast mitochondrial escape protein 6) | *Saccharomyces cerevisiae* |
| P50276 | High-affinity methionine permease | *Saccharomyces cerevisiae* |
| P22211 | Nitrogen permease reactivator protein (EC 2.7.11.1) (Serine/threonine-protein kinase NPR1) | *Saccharomyces cerevisiae* |
| P50111 | Protein ZDS1 (Protein NRC1) (RT2GS1) | *Saccharomyces cerevisiae* |
| P53397 | N-glycosylase/DNA lyase [Includes: 8-oxoguanine DNA glycosylase (EC 3.2.2.-); DNA-(apurinic or apyrimidinic site) lyase (AP lyase) (EC 4.2.99.18)] | *Saccharomyces cerevisiae* |
| P07213 | Mitochondrial import receptor subunit TOM70 (70 kDa mitochondrial outer membrane protein) (Translocase of outer membrane 70 kDa subunit) | *Saccharomyces cerevisiae* |
| P52867 | Dolichyl-phosphate-mannose--protein mannosyltransferase 5 (EC 2.4.1.109) | *Saccharomyces cerevisiae* |
| P53552 | THO complex subunit 2 (Low dye-binding protein 5) (THO complex subunit RLR1) (Zinc-regulated gene 13 protein) | *Saccharomyces cerevisiae* |
| P12686 | 37S ribosomal protein MRP13, mitochondrial (Mitochondrial small ribosomal subunit protein mS44) (YmS-A) | *Saccharomyces cerevisiae* |
| P53047 | Protein RTA1 | *Saccharomyces cerevisiae* |
| P15624 | Phenylalanine--tRNA ligase beta subunit (EC 6.1.1.20) | *Saccharomyces cerevisiae* |
| P07806 | Valine--tRNA ligase, mitochondrial (EC 6.1.1.9) (Valyl-tRNA synthetase) (ValRS) | *Saccharomyces cerevisiae* |
| P29509 | Thioredoxin reductase 1 (EC 1.8.1.9) | *Saccharomyces cerevisiae* |
| P50102 | Ubiquitin carboxyl-terminal hydrolase 8 (EC 3.4.19.12) (Deubiquitinating enzyme 8) (Ubiquitin thioesterase 8) (Ubiquitin-specific-processing protease 8) | *Saccharomyces cerevisiae* |
| P39730 | Eukaryotic translation initiation factor 5B (eIF-5B) (EC 3.6.5.3) (Translation initiation factor IF-2) | *Saccharomyces cerevisiae* |
| P53203 | Peroxisomal membrane protein PEX31 (Peroxin-31) | *Saccharomyces cerevisiae* |
| P53204 | Nicotinamide/nicotinic acid mononucleotide adenylyltransferase 2 (EC 2.7.7.1) (EC 2.7.7.18) | *Saccharomyces cerevisiae* |
| P53214 | Protein MTL1 (MID two-like protein 1) | *Saccharomyces cerevisiae* |
| P53217 | Uncharacterized membrane protein YGR026W | *Saccharomyces cerevisiae* |
| P53220 | Mitochondrial import inner membrane translocase subunit TIM21 | *Saccharomyces cerevisiae* |
| P53235 | Eukaryotic translation initiation factor 2A (eIF-2A) | *Saccharomyces cerevisiae* |
| P53257 | Mitochondrial thiamine pyrophosphate carrier 1 | *Saccharomyces cerevisiae* |
| P53266 | Cytochrome oxidase assembly protein SHY1 | *Saccharomyces cerevisiae* |
| P53301 | Probable glycosidase CRH1 (EC 3.2.-.-) (Congo red hypersensitive protein 1) | *Saccharomyces cerevisiae* |
| P53320 | Mitochondrial carrier protein MTM1 (Manganese trafficking factor for mitochondrial SOD2) | *Saccharomyces cerevisiae* |
| P53198 | Protein ERP6 | *Saccharomyces cerevisiae* |
| P53197 | APC/C activator protein CDH1 (CDC20 homolog 1) (Homolog of CDC twenty 1) | *Saccharomyces cerevisiae* |
| P53196 | 26S proteasome regulatory subunit RPN14 (Proteasome non-ATPase subunit 14) | *Saccharomyces cerevisiae* |
| P53148 | Spindle pole body component SPC105 (105 kDa spindle pole component protein) | *Saccharomyces cerevisiae* |
| P53131 | Pre-mRNA-splicing factor ATP-dependent RNA helicase PRP43 (EC 3.6.4.13) (Helicase JA1) | *Saccharomyces cerevisiae* |
| P38616 | Protein YGP1 (GP38) | *Saccharomyces cerevisiae* |
| P53038 | Telomere length regulation protein TEL2 | *Saccharomyces cerevisiae* |
| P31688 | Trehalose-phosphatase (EC 3.1.3.12) (Trehalose synthase complex catalytic subunit TPS2) | *Saccharomyces cerevisiae* |
| P53104 | Serine/threonine-protein kinase ATG1 (EC 2.7.11.1) (Autophagy protein 3) (Autophagy-related protein 1) | *Saccharomyces cerevisiae* |
| P17649 | 4-aminobutyrate aminotransferase (EC 2.6.1.19) (GABA aminotransferase) | *Saccharomyces cerevisiae* |
| P54003 | Protein SUR7 | *Saccharomyces cerevisiae* |
| P50104 | Probable transcriptional regulatory protein STB4 | *Saccharomyces cerevisiae* |
| P53838 | Boron transporter 1 | *Saccharomyces cerevisiae* |
| P53834 | Hsp90 co-chaperone HCH1 (High-copy Hsp90 suppressor protein 1) | *Saccharomyces cerevisiae* |
| P53833 | Ribonucleases P/MRP protein subunit POP3 (RNA-processing protein POP3) | *Saccharomyces cerevisiae* |
| P53829 | Protein CAF40 (40 kDa CCR4-associated factor) | *Saccharomyces cerevisiae* |
| P53746 | Ferric reductase transmembrane component 4 (EC 1.16.1.9) (Ferric-chelate reductase 4) | *Saccharomyces cerevisiae* |
| P53752 | Uncharacterized membrane glycoprotein YNR066C | *Saccharomyces cerevisiae* |
| P53756 | ABC transporter ATP-binding protein/permease PDR18 | *Saccharomyces cerevisiae* |
| P53959 | Conserved oligomeric Golgi complex subunit 6 (COG complex subunit 6) | *Saccharomyces cerevisiae* |
| P53947 | Vacuolar membrane protein YNL058C | *Saccharomyces cerevisiae* |
| P53946 | Actin-related protein 5 (Actin-like protein ARP5) | *Saccharomyces cerevisiae* |
| P53937 | 37S ribosomal protein SWS2, mitochondrial (Mitochondrial small ribosomal subunit protein uS13m) | *Saccharomyces cerevisiae* |
| P53923 | Cytoplasmic tRNA 2-thiolation protein 2 (Needs CLA4 to survive protein 2) (Thiolation of uridine in cytoplasmic tRNA protein 2) | *Saccharomyces cerevisiae* |
| P53921 | 54S ribosomal protein bL35m (Mitochondrial large ribosomal subunit protein bL35m) | *Saccharomyces cerevisiae* |
| P53918 | Uncharacterized transporter ESBP6 | *Saccharomyces cerevisiae* |
| P53915 | Nicotinamide riboside kinase (NRK) (NmR-K) (EC 2.7.1.22) (Nicotinic acid riboside kinase) (EC 2.7.1.173) (Ribosylnicotinamide kinase) (RNK) (Ribosylnicotinic acid kinase) | *Saccharomyces cerevisiae* |
| P53899 | CDC48-associated ubiquitin-like/zinc finger protein 1 (CDC48-associated UBL/Zn-finger protein 1) | *Saccharomyces cerevisiae* |
| P53890 | Bud neck protein 5 | *Saccharomyces cerevisiae* |
| P53866 | Protein SQS1 (Squelch of splicing suppression protein 1) | *Saccharomyces cerevisiae* |
| P53863 | J protein JJJ1 | *Saccharomyces cerevisiae* |
| P53854 | Pre-mRNA-splicing factor CWC25 (Complexed with CEF1 protein 25) | *Saccharomyces cerevisiae* |
| P19541 | Regulator of drug sensitivity 2 | *Saccharomyces cerevisiae* |
| P36008 | Elongation factor 1-gamma 2 (EF-1-gamma 2) (Eukaryotic elongation factor 1Bgamma 2) (eEF1Bgamma 2) (Translation elongation factor 1B gamma 2) | *Saccharomyces cerevisiae* |
| O13553 | Putative uncharacterized protein YLR317W | *Saccharomyces cerevisiae* |
| Q99186 | AP-2 complex subunit mu (Adaptin medium chain APM4) (Clathrin assembly protein complex 2 mu medium chain) (Clathrin coat assembly protein AP50) (Clathrin coat-associated protein AP50) (Mu2-adaptin) (Plasma membrane adaptor AP-2 50 kDa protein) | *Saccharomyces cerevisiae* |
| Q12284 | FAD-linked sulfhydryl oxidase ERV2 (EC 1.8.3.2) | *Saccharomyces cerevisiae* |
| Q04772 | Increased recombination centers protein 21 | *Saccharomyces cerevisiae* |
| Q03655 | Probable 1,3-beta-glucanosyltransferase GAS3 (EC 2.4.1.-) (Glycolipid-anchored surface protein 3) | *Saccharomyces cerevisiae* |
| Q12024 | Ribosome biogenesis protein YTM1 (Microtubule-associated protein YTM1) | *Saccharomyces cerevisiae* |
| Q03177 | WD repeat-containing protein YMR102C | *Saccharomyces cerevisiae* |
| Q03210 | Probable RNA exonuclease NGL3 (EC 3.1.-.-) | *Saccharomyces cerevisiae* |
| Q04693 | Pre-mRNA-splicing factor RSE1 (RNA splicing and ER to Golgi transport factor 1) (Spliceosome-associated protein 130) | *Saccharomyces cerevisiae* |
| Q04304 | UPF0659 protein YMR090W | *Saccharomyces cerevisiae* |
| Q03218 | Mitochondrial metal transporter 1 | *Saccharomyces cerevisiae* |
| Q12387 | N-terminal acetyltransferase B complex subunit MDM20 | *Saccharomyces cerevisiae* |
| Q08822 | Probable electron transfer flavoprotein-ubiquinone oxidoreductase, mitochondrial (ETF-QO) (ETF-ubiquinone oxidoreductase) (EC 1.5.5.1) | *Saccharomyces cerevisiae* |
| Q12446 | Proline-rich protein LAS17 | *Saccharomyces cerevisiae* |
| P89102 | Exocyst complex component SEC5 | *Saccharomyces cerevisiae* |
| Q12512 | Protein ZPS1 | *Saccharomyces cerevisiae* |
| Q12196 | Serine/threonine-protein kinase RIO1 (EC 2.7.11.1) (EC 3.6.3.-) (Ribosomal RNA-processing protein 10) | *Saccharomyces cerevisiae* |
| Q07657 | Seventh homolog of septin 1 (Septation protein 7) | *Saccharomyces cerevisiae* |
| Q07953 | Ribosome maturation protein SDO1 | *Saccharomyces cerevisiae* |
| Q12086 | DNA damage-inducible protein DIN7 (EC 3.1.-.-) | *Saccharomyces cerevisiae* |
| Q05854 | Uncharacterized transcriptional regulatory protein YLR278C | *Saccharomyces cerevisiae* |
| P38144 | ISWI chromatin-remodeling complex ATPase ISW1 (EC 3.6.4.-) | *Saccharomyces cerevisiae* |
| P14906 | Protein translocation protein SEC63 (Protein NPL1) | *Saccharomyces cerevisiae* |
| P08417 | Fumarate hydratase, mitochondrial (Fumarase) (EC 4.2.1.2) | *Saccharomyces cerevisiae* |
| P53064 | RNA polymerase-associated protein RTF1 | *Saccharomyces cerevisiae* |
| P47029 | Arrestin-related trafficking adapter 3 (Arrestin-like protein 2) | *Saccharomyces cerevisiae* |
| P40068 | Transcriptional activator FLO8 (Protein PDH5) | *Saccharomyces cerevisiae* |
| P32770 | Asparagine-rich protein (Protein ARP) | *Saccharomyces cerevisiae* |
| P40353 | Alcohol O-acetyltransferase 1 (AATase 1) (EC 2.3.1.84) (EC 3.1.2.20) | *Saccharomyces cerevisiae* |
| P19659 | Mediator of RNA polymerase II transcription subunit 15 (Autonomous replication regulatory protein 3) (Basal expression activator protein 1) (Defective silencing suppressor protein 4) (Mediator complex subunit 15) (Transcription regulatory protein GAL11) (Ty insertion suppressor protein 13) | *Saccharomyces cerevisiae* |
| P39904 | Vacuolar protein sorting-associated protein 52 (Suppressor of actin mutation protein 2) | *Saccharomyces cerevisiae* |
| P11075 | Protein transport protein SEC7 | *Saccharomyces cerevisiae* |
| P32913 | Vacuolar protein sorting-associated protein 17 (Carboxypeptidase Y-deficient protein 21) | *Saccharomyces cerevisiae* |
| P37370 | Verprolin | *Saccharomyces cerevisiae* |
| P12611 | Growth regulation protein | *Saccharomyces cerevisiae* |
| P07263 | Histidine--tRNA ligase, mitochondrial (EC 6.1.1.21) | *Saccharomyces cerevisiae* |
| P25605 | Acetolactate synthase small subunit, mitochondrial | *Saccharomyces cerevisiae* |
| Q06005 | Octanoyltransferase, mitochondrial (EC 2.3.1.181) | *Saccharomyces cerevisiae* |
| P25039 | Elongation factor G, mitochondrial (EF-Gmt) (Elongation factor G 1, mitochondrial) (mEF-G 1) (Elongation factor G1) | *Saccharomyces cerevisiae* |
| P38066 | GTP cyclohydrolase-2 (EC 3.5.4.25) (GTP cyclohydrolase II) | *Saccharomyces cerevisiae* |
| P00175 | Cytochrome b2, mitochondrial (EC 1.1.2.3) | *Saccharomyces cerevisiae* |
| Q12265 | Ribose-phosphate pyrophosphokinase 5 (EC 2.7.6.1) | *Saccharomyces cerevisiae* |
| Q12325 | Sulfate permease 2 (High-affinity sulfate transporter 2) | *Saccharomyces cerevisiae* |
| P32602 | Alpha-soluble NSF attachment protein (SNAP-alpha) | *Saccharomyces cerevisiae* |
| Q9ZZX1 | Intron-encoded DNA endonuclease aI5 alpha (DNA endonuclease I-SceIV) | *Saccharomyces cerevisiae* |
| P38215 | Putative uncharacterized protein YBR013C | *Saccharomyces cerevisiae* |
| P32469 | Diphthine methyl ester synthase (EC 2.1.1.314) | *Saccharomyces cerevisiae* |
| Q04121 | Endosomal/prevacuolar sodium/hydrogen exchanger (Endosomal/prevacuolar Na(+)/H(+) exchanger) (Vacuolar protein sorting-associated protein 44) | *Saccharomyces cerevisiae* |
| P39109 | Metal resistance protein YCF1 (ABC-type Cd(2+) transporter) (EC 7.2.2.2) (ABC-type glutathione-S-conjugate transporter) (EC 7.6.2.3) (Yeast cadmium factor 1) | *Saccharomyces cerevisiae* |
| Q07560 | Cardiolipin synthase (CMP-forming) (CLS) (EC 2.7.8.41) | *Saccharomyces cerevisiae* |
| Q35811 | Putative uncharacterized protein Q0092, mitochondrial | *Saccharomyces cerevisiae* |
| P18562 | Uracil phosphoribosyltransferase (UPRTase) (EC 2.4.2.9) (UMP pyrophosphorylase) | *Saccharomyces cerevisiae* |
| P03962 | Orotidine 5'-phosphate decarboxylase (EC 4.1.1.23) | *Saccharomyces cerevisiae* |
| Q06708 | Vacuole morphology and inheritance protein 14 (Swollen vacuole phenotype 2 protein) | *Saccharomyces cerevisiae* |
| Q08979 | Kelch repeat-containing protein 3 | *Saccharomyces cerevisiae* |
| Q99299 | Altered inheritance of mitochondria protein 44 | *Saccharomyces cerevisiae* |
| Q08236 | Target of rapamycin complex 2 subunit AVO1 (TORC2 subunit AVO1) | *Saccharomyces cerevisiae* |
| Q01846 | Structural protein MDM1 (Mitochondrial distribution and morphology protein 1) | *Saccharomyces cerevisiae* |
| Q03707 | Inner nuclear membrane protein SRC1 (Helix-extension-helix domain-containing protein 1) | *Saccharomyces cerevisiae* |
| Q12451 | Oxysterol-binding protein homolog 2 | *Saccharomyces cerevisiae* |
| P11972 | Protein SST2 | *Saccharomyces cerevisiae* |
| Q04062 | 26S proteasome regulatory subunit RPN9 (Proteasome non-ATPase subunit 7) | *Saccharomyces cerevisiae* |
| Q12250 | 26S proteasome regulatory subunit RPN5 (Proteasome non-ATPase subunit 5) | *Saccharomyces cerevisiae* |
| P17883 | Superkiller protein 3 | *Saccharomyces cerevisiae* |
| Q06648 | GTPase-interacting component 2 | *Saccharomyces cerevisiae* |
| Q8KRK0 | RpoB protein (Fragment) | *Pediococcus pentosaceus* |
| E9PA30 | K-2 killer toxin | *Saccharomyces cerevisiae* |
| Q9URQ3 | tRNA-specific adenosine deaminase subunit TAD3 | *Saccharomyces cerevisiae* |
| P52286 | Suppressor of kinetochore protein 1 | *Saccharomyces cerevisiae* |
| Q12267 | Structural maintenance of chromosomes protein 4 | *Saccharomyces cerevisiae* |
| Q03759 | Uncharacterized protein YML108W | *Saccharomyces cerevisiae* |
| P06169 | Pyruvate decarboxylase isozyme 1 (EC 4.1.1.-) (EC 4.1.1.43) (EC 4.1.1.72) (EC 4.1.1.74) (Thiamine pyrophosphate-dependent 2-oxo-acid decarboxylase) (2ODC) | *Saccharomyces cerevisiae* |
| Q06156 | Condensin complex subunit 1 (XCAP-D2 homolog) | *Saccharomyces cerevisiae* |
| P25356 | Beige protein homolog 1 | *Saccharomyces cerevisiae* |
| P25578 | CDP-diacylglycerol--glycerol-3-phosphate 3-phosphatidyltransferase (EC 2.7.8.5) | *Saccharomyces cerevisiae* |
| P25602 | Putative uncharacterized protein YCL076W | *Saccharomyces cerevisiae* |
| A2P2J0 | Uncharacterized protein | *Saccharomyces cerevisiae* |
| P25390 | Serine/threonine-protein kinase SSK22 (EC 2.7.11.1) | *Saccharomyces cerevisiae* |
| P38257 | Crossover junction endonuclease MMS4 (EC 3.1.22.-) | *Saccharomyces cerevisiae* |
| Q06350 | Sporulation-specific chitinase 2 (EC 3.2.1.14) | *Saccharomyces cerevisiae* |
| Q03103 | Endoplasmic oxidoreductin-1 (EC 1.8.4.-) (Endoplasmic reticulum oxidoreductase protein 1) | *Saccharomyces cerevisiae* |
| Q12743 | DER1-like family member protein 1 | *Saccharomyces cerevisiae* |
| P53889 | Uncharacterized mitochondrial hydrolase FMP41 (EC 3.-.-.-) | *Saccharomyces cerevisiae* |
| P32339 | Heme-binding protein HMX1 | *Saccharomyces cerevisiae* |
| P53876 | Uncharacterized protein YNL184C | *Saccharomyces cerevisiae* |
| P40461 | Putative uncharacterized protein YIL141W | *Saccharomyces cerevisiae* |
| P25454 | DNA repair protein RAD51 | *Saccharomyces cerevisiae* |
| P38228 | Mitochondrial chaperone TCM62 | *Saccharomyces cerevisiae* |
| P13259 | Choline-phosphate cytidylyltransferase (EC 2.7.7.15) | *Saccharomyces cerevisiae* |
| Q07349 | MIOREX complex component 9 | *Saccharomyces cerevisiae* |
| P11353 | Oxygen-dependent coproporphyrinogen-III oxidase (COX) (Coprogen oxidase) (Coproporphyrinogenase) (EC 1.3.3.3) | *Saccharomyces cerevisiae* |
| Q06011 | Protein ECM19 (Extracellular mutant protein 19) | *Saccharomyces cerevisiae* |
| Q3T4E1 | NADH-ubiquinone oxidoreductase chain 5 (EC 7.1.1.2) | *Rhizopus oryzae* |
| Q3T4E6 | Orf376 | *Rhizopus oryzae* |
| Q06510 | Lysophosphatidylcholine acyltransferase (EC 2.3.1.23) | *Saccharomyces cerevisiae* |
| P39081 | Protein PCF11 (protein 1 of CF I) | *Saccharomyces cerevisiae* |
| O60200 | Mitochondrial distribution and morphology protein 35 | *Saccharomyces cerevisiae* |
| Q4VZZ8 | Mobilization protein | *Pediococcus pentosaceus* |
| Q4VZZ7 | Replication protein | *Pediococcus pentosaceus* |
| P32471 | Elongation factor 1-beta (EF-1-beta) (Eukaryotic elongation factor 1Balpha) (eEF1Balpha) (Translation elongation factor 1B alpha) | *Saccharomyces cerevisiae* |
| P53216 | Uncharacterized protein YGR025W | *Saccharomyces cerevisiae* |
| P53936 | Putative uncharacterized protein YNL089C | *Saccharomyces cerevisiae* |
| Q04048 | Pre-mRNA-splicing factor SYF1 (PRP19-associated complex protein 90) | *Saccharomyces cerevisiae* |
| Q03921 | Protein dopey | *Saccharomyces cerevisiae* |
| Q04341 | Mitochondrial intermembrane space cysteine motif-containing protein MIX14 | *Saccharomyces cerevisiae* |
| Q04430 | Pantothenate kinase CAB1 (EC 2.7.1.33) (Coenzyme A biosynthesis protein 1) | *Saccharomyces cerevisiae* |
| Q05518 | Protein PAL1 (Pears and lemons protein 1) | *Saccharomyces cerevisiae* |
| Q07395 | Synchronized import protein 1 (Symportin 1) | *Saccharomyces cerevisiae* |
| Q07684 | Morphogenetic regulator of filamentous growth protein 1 | *Saccharomyces cerevisiae* |
| Q08886 | Guanine nucleotide-binding protein subunit beta 1 (Gbeta mimic kelch protein 1) | *Saccharomyces cerevisiae* |
| Q08952 | Oxidation resistance protein 1 | *Saccharomyces cerevisiae* |
| Q08966 | PHO85 cyclin-8 | *Saccharomyces cerevisiae* |
| Q08985 | Homocysteine S-methyltransferase 2 (EC 2.1.1.10) | *Saccharomyces cerevisiae* |
| Q12249 | Putative uncharacterized protein YOR218C | *Saccharomyces cerevisiae* |
| Q12270 | Rhomboid protein 2 (EC 3.4.21.-) | *Saccharomyces cerevisiae* |
| Q12427 | Protein STB3 (SIN3-binding protein 3) | *Saccharomyces cerevisiae* |
| Q9ZZW1 | Putative uncharacterized protein Q0182, mitochondrial | *Saccharomyces cerevisiae* |
| Q05473 | MIOREX complex component 8 | *Saccharomyces cerevisiae* |
| Q08750 | Protein MUM3 (Muddled meiosis protein 3) | *Saccharomyces cerevisiae* |
| Q08991 | Fluoride export protein 2 | *Saccharomyces cerevisiae* |
| Q12083 | DNA mismatch repair protein MLH3 (MutL protein homolog 3) | *Saccharomyces cerevisiae* |
| Q12296 | Protein MAM3 | *Saccharomyces cerevisiae* |
| Q12447 | Polyamine N-acetyltransferase 1 (EC 2.3.1.-) (Arylalkylamine N-acetyltransferase homolog) (scAANAT) | *Saccharomyces cerevisiae* |
| Q99216 | Pre-rRNA-processing protein PNO1 (Partner of NOB1) (Ribosomal RNA-processing protein 20) | *Saccharomyces cerevisiae* |
| Q06032 | Chromosome stability protein 9 (Molecular zipper protein 3) | *Saccharomyces cerevisiae* |
| Q06164 | E3 ubiquitin-protein ligase substrate receptor MMS22 (Methyl methanesulfonate-sensitivity protein 22) (Synthetically lethal with MCM10 protein 2) | *Saccharomyces cerevisiae* |
| Q06224 | Endoribonuclease YSH1 (EC 3.1.27.-) (Yeast 73 kDa homolog 1) (mRNA 3'-end-processing protein YSH1) | *Saccharomyces cerevisiae* |
| Q06266 | Protein TOS4 (Target of SBF protein 4) | *Saccharomyces cerevisiae* |
| Q06538 | Calcium permeable stress-gated cation channel 1 (ScCSC1) | *Saccharomyces cerevisiae* |
| Q06709 | Cytoplasmic 60S subunit biogenesis factor REH1 (REI1-homolog 1) (pre-60S factor REH1) | *Saccharomyces cerevisiae* |
| Q12098 | Structure-specific endonuclease subunit SLX4 (Synthetic lethal of unknown function protein 4) | *Saccharomyces cerevisiae* |
| Q12172 | Zinc finger transcription factor YRR1 (Pleiotropic drug-resistance protein 2) | *Saccharomyces cerevisiae* |
| Q12416 | G1-specific transcriptional repressor WHI5 | *Saccharomyces cerevisiae* |
| Q12517 | mRNA-decapping enzyme subunit 1 | *Saccharomyces cerevisiae* |
| Q07530 | Uncharacterized oxidoreductase YDL114W (EC 1.-.-.-) | *Saccharomyces cerevisiae* |
| Q12340 | Zinc finger transcription factor YRM1 (Reveromycin resistance modulator 1) | *Saccharomyces cerevisiae* |
| Q02950 | 37S ribosomal protein MRP51, mitochondrial (Mitochondrial small ribosomal subunit protein bS1m) | *Saccharomyces cerevisiae* |
| Q03306 | Serine/threonine-protein kinase PKH3 (EC 2.7.11.1) (Pkb-activating kinase homolog 3) | *Saccharomyces cerevisiae* |
| Q05549 | ATP-dependent helicase HRQ1 (EC 3.6.4.12) (Homologous to recQ protein 1) | *Saccharomyces cerevisiae* |
| Q06106 | Multiple RNA-binding domain-containing protein 1 | *Saccharomyces cerevisiae* |
| Q06107 | Uncharacterized TLC domain-containing protein YPR114W | *Saccharomyces cerevisiae* |
| Q06451 | Polyamine transporter 3 | *Saccharomyces cerevisiae* |
| Q06505 | Transcription factor SPN1 (Interacts with SPT6 protein 1) (Suppresses postrecruitment functions protein 1) | *Saccharomyces cerevisiae* |
| Q06511 | Ribosomal RNA-processing protein 15 | *Saccharomyces cerevisiae* |
| Q06595 | Maltose fermentation regulatory protein YPR196W | *Saccharomyces cerevisiae* |
| Q06817 | Glycine--tRNA ligase 2 (EC 6.1.1.14) (Diadenosine tetraphosphate synthetase) (Ap4A synthetase) (EC 2.7.7.-) (Glycyl-tRNA synthetase 2) (GlyRS 2) (GlyRS2) | *Saccharomyces cerevisiae* |
| Q07895 | FAS1 domain-containing protein YLR001C | *Saccharomyces cerevisiae* |
| Q08817 | Leucine-rich repeat-containing protein SOG2 | *Saccharomyces cerevisiae* |
| Q08920 | Nuclear cap-binding protein subunit 2 (20 kDa nuclear cap-binding protein) (NCBP 20 kDa subunit) (CBP20) | *Saccharomyces cerevisiae* |
| Q08924 | Regulator of Ty1 transposition protein 10 (Endosomal recycling protein 2) (tRNA (guanosine(34)-2'-O)-methyltransferase non-catalytic subunit TRM734) | *Saccharomyces cerevisiae* |
| Q12090 | RNA exonuclease 3 (EC 3.1.-.-) | *Saccharomyces cerevisiae* |
| Q12100 | Probable serine/threonine-protein kinase RTK1 (EC 2.7.11.1) (Ribosome biogenesis and tRNA synthetase-associated kinase 1) | *Saccharomyces cerevisiae* |
| Q12161 | RING finger protein PSH1 (POB3/SPT16 histone-associated protein 1) | *Saccharomyces cerevisiae* |
| Q12230 | Sphingolipid long chain base-responsive protein LSP1 | *Saccharomyces cerevisiae* |
| Q12346 | Uncharacterized membrane protein YPR071W | *Saccharomyces cerevisiae* |
| Q12457 | RNA polymerase I termination factor (NTS1 silencing protein 1) | *Saccharomyces cerevisiae* |
| Q99247 | DUB-associated factor 1 | *Saccharomyces cerevisiae* |
| Q9ZZW7 | Cytochrome b mRNA maturase bI3 | *Saccharomyces cerevisiae* |
| Q2VC81 | Glucoamylase (Glucoamylase A) | *Rhizopus oryzae* |
| P41735 | 5-demethoxyubiquinone hydroxylase, mitochondrial (DMQ hydroxylase) (EC 1.14.99.60) (Catabolite repression protein 5) (Ubiquinone biosynthesis monooxygenase COQ7) | *Saccharomyces cerevisiae* |
| Q01802 | Aspartate aminotransferase, mitochondrial (EC 2.6.1.1) (Transaminase A) | *Saccharomyces cerevisiae* |
| P43583 | Proteasome activator BLM10 (Bleomycin resistance protein BLM10) | *Saccharomyces cerevisiae* |
| P80235 | Putative mitochondrial carnitine O-acetyltransferase (EC 2.3.1.7) | *Saccharomyces cerevisiae* |
| P37292 | Serine hydroxymethyltransferase, mitochondrial (SHMT) (EC 2.1.2.1) | *Saccharomyces cerevisiae* |
| P35845 | Oxysterol-binding protein homolog 1 | *Saccharomyces cerevisiae* |
| Q3E792 | 40S ribosomal protein S25-A (RP45) (S31) (Small ribosomal subunit protein eS25-A) (YS23) | *Saccharomyces cerevisiae* |
| P53200 | Protein-lysine N-methyltransferase EFM5 (EC 2.1.1.-) (Elongation factor methyltransferase 5) (N(6)-adenine-specific DNA methyltransferase-like 1) | *Saccharomyces cerevisiae* |
| Q06682 | UBX domain-containing protein 5 | *Saccharomyces cerevisiae* |
| P03878 | Intron-encoded DNA endonuclease aI4 (DNA endonuclease I-SceII) | *Saccharomyces cerevisiae* |
| P27895 | Kinesin-like protein CIN8 (Chromosome instability protein 8) | *Saccharomyces cerevisiae* |
| P36163 | Mitochondrial metalloendopeptidase OMA1 (EC 3.4.24.-) | *Saccharomyces cerevisiae* |
| Q02981 | ABC1 family protein YPL109C, mitochondrial | *Saccharomyces cerevisiae* |
| Q08687 | Translation machinery-associated protein 16 | *Saccharomyces cerevisiae* |
| Q3E829 | Inner kinetochore subunit MHF2 (CENP-X homolog) (Constitutive centromere-associated network protein MHF2) (MHF histone-fold complex subunit 2) (MPH1-associated histone-fold protein 2) | *Saccharomyces cerevisiae* |
| P14242 | DNA mismatch repair protein PMS1 (Postmeiotic segregation protein 1) | *Saccharomyces cerevisiae* |
| P33753 | tRNA (uracil(54)-C(5))-methyltransferase (EC 2.1.1.35) (Transfer RNA methyltransferase 2) (tRNA(m5U54)-methyltransferase) | *Saccharomyces cerevisiae* |
| Q12680 | Glutamate synthase [NADH] (EC 1.4.1.14) (NADH-GOGAT) | *Saccharomyces cerevisiae* |
| P51979 | ATP-dependent DNA helicase MER3 (EC 3.6.4.12) (Protein HFM1) | *Saccharomyces cerevisiae* |
| P46655 | Glutamate--tRNA ligase, cytoplasmic (EC 6.1.1.17) (Glutamyl-tRNA synthetase) ((c)ERS) (GluRS) (P85) | *Saccharomyces cerevisiae* |
| Q3E7Y5 | Uncharacterized helicase-like protein YBL111C | *Saccharomyces cerevisiae* |
| P10870 | Low glucose sensor SNF3 (High-affinity glucose receptor SNF3) (High-affinity transporter-like sensor SNF3) (Sucrose nonfermenting protein 3) | *Saccharomyces cerevisiae* |
| Q03G26 | Uncharacterized protein | *Pediococcus pentosaceus* |
| Q03G11 | Uncharacterized protein | *Pediococcus pentosaceus* |
| P40484 | DBF2 kinase activator protein MOB1 (MPS1 binder 1) (Maintenance of ploidy protein MOB1) | *Saccharomyces cerevisiae* |
| A7J3S1 | Priming glycosyltransferase (Fragment) | *Pediococcus pentosaceus* |
| Q05164 | Haze protective factor 1 | *Saccharomyces cerevisiae* |
| Q3T4F3 | NADH-ubiquinone oxidoreductase chain 2 (EC 7.1.1.2) (NADH dehydrogenase subunit 2) | *Rhizopus oryzae* |
| Q03D70 | ATP-dependent helicase/deoxyribonuclease subunit B (EC 3.1.-.-) (EC 3.6.4.12) (ATP-dependent helicase/nuclease RexB) | *Pediococcus pentosaceus* |
| Q03DS0 | Ornithine carbamoyltransferase (OTCase) (EC 2.1.3.3) | *Pediococcus pentosaceus* |
| Q03EI3 | Glucose-6-phosphate isomerase (GPI) (EC 5.3.1.9) (Phosphoglucose isomerase) (PGI) (Phosphohexose isomerase) (PHI) | *Pediococcus pentosaceus* |
| Q03EL3 | ATP synthase gamma chain (ATP synthase F1 sector gamma subunit) (F-ATPase gamma subunit) | *Pediococcus pentosaceus* |
| Q03EY9 | Isoleucine--tRNA ligase (EC 6.1.1.5) (Isoleucyl-tRNA synthetase) (IleRS) | *Pediococcus pentosaceus* |
| Q03F22 | 30S ribosomal protein S15 | *Pediococcus pentosaceus* |
| Q03F82 | Segregation and condensation protein B | *Pediococcus pentosaceus* |
| Q03FY3 | Methionyl-tRNA formyltransferase (EC 2.1.2.9) | *Pediococcus pentosaceus* |
| Q03GW7 | Phosphoglycerate kinase (EC 2.7.2.3) | *Pediococcus pentosaceus* |
| Q03HB3 | Aspartate carbamoyltransferase (EC 2.1.3.2) | *Pediococcus pentosaceus* |
| P0C2H9 | 60S ribosomal protein L31-B (L34) (Large ribosomal subunit protein eL31-B) (YL28) | *Saccharomyces cerevisiae* |
| P38797 | Protein phosphatase 2C homolog 7, mitochondrial (PP2C-7) (EC 3.1.3.16) | *Saccharomyces cerevisiae* |
| A7A2C3 | Conserved protein (Fragment) | *Saccharomyces cerevisiae* |
| A6ZY79 | Anaphase promoting complex (APC) subunit | *Saccharomyces cerevisiae* |
| A6ZYA9 | Uncharacterized protein | *Saccharomyces cerevisiae* |
| A6ZTJ6 | Conserved protein | *Saccharomyces cerevisiae* |
| A6ZSJ4 | Uncharacterized protein | *Saccharomyces cerevisiae* |
| A6ZRE5 | Uncharacterized protein (Fragment) | *Saccharomyces cerevisiae* |
| P00431 | Cytochrome c peroxidase, mitochondrial (CCP) (EC 1.11.1.5) | *Saccharomyces cerevisiae* |
| P54860 | E4 ubiquitin-protein ligase UFD2 (EC 2.3.2.27) | *Saccharomyces cerevisiae* |
| P49775 | Bis(5'-adenosyl)-triphosphatase (EC 3.6.1.29) (AP3A hydrolase) (AP3Aase) | *Saccharomyces cerevisiae* |
| P42844 | Mitochondrial protein import protein ZIM17 | *Saccharomyces cerevisiae* |
| Q02206 | Chromatin structure-remodeling complex subunit RSC4 | *Saccharomyces cerevisiae* |
| A9JQ55 | DNA-directed RNA polymerase subunit alpha (Fragment) | *Pediococcus pentosaceus* |
| A9JPZ9 | Phenylalanyl-tRNA synthetase alpha subunit (Fragment) | *Pediococcus pentosaceus* |
| P33309 | Protein DOM34 (EC 3.1.-.-) | *Saccharomyces cerevisiae* |
| P61830 | Histone H3 | *Saccharomyces cerevisiae* |
| B3LFV7 | Arginyl-tRNA synthetase | *Saccharomyces cerevisiae* |
| B3LGY9 | Dolichyl-phosphate-mannose-protein mannosyltransferase 1 | *Saccharomyces cerevisiae* |
| B3LH96 | COP9 signalosome complex subunit 5 (EC 3.4.-.-) | *Saccharomyces cerevisiae* |
| B3LKF9 | Uncharacterized protein | *Saccharomyces cerevisiae* |
| B3LPI8 | Protein HOL1 | *Saccharomyces cerevisiae* |
| P32589 | Heat shock protein homolog SSE1 (Chaperone protein MSI3) | *Saccharomyces cerevisiae* |
| Q07418 | Peroxisomal membrane protein import receptor PEX19 (Peroxin-19) | *Saccharomyces cerevisiae* |
| A6ZTT5 | Alcohol dehydrogenase 4 (EC 1.1.1.1) (Alcohol dehydrogenase IV) (ADHIV) | *Saccharomyces cerevisiae* |
| B5VTY7 | Dimer_Tnp_hAT domain-containing protein | *Saccharomyces cerevisiae* |
| B5VTX1 | YPR194Cp-like protein (Fragment) | *Saccharomyces cerevisiae* |
| B5VSE8 | YOR269Wp-like protein | *Saccharomyces cerevisiae* |
| B5VS75 | YOR191Wp-like protein (Fragment) | *Saccharomyces cerevisiae* |
| B5VQV8 | YNL085Wp-like protein (Fragment) | *Saccharomyces cerevisiae* |
| B5VQ55 | Uncharacterized protein | *Saccharomyces cerevisiae* |
| B5VQ25 | YMR259Cp-like protein (Fragment) | *Saccharomyces cerevisiae* |
| B5VPK8 | YMR083Wp-like protein (Fragment) | *Saccharomyces cerevisiae* |
| B5VPF6 | YMR028Wp-like protein | *Saccharomyces cerevisiae* |
| B5VPC8 | Uncharacterized protein | *Saccharomyces cerevisiae* |
| B5VMJ1 | Uncharacterized protein | *Saccharomyces cerevisiae* |
| B5VMC3 | Uncharacterized protein | *Saccharomyces cerevisiae* |
| B5VM70 | Uncharacterized protein | *Saccharomyces cerevisiae* |
| B5VM52 | YKL105Cp-like protein (Fragment) | *Saccharomyces cerevisiae* |
| B5VL45 | YJL132Wp-like protein | *Saccharomyces cerevisiae* |
| B5VK35 | YHR102Wp-like protein | *Saccharomyces cerevisiae* |
| B5VJB4 | YGR170Wp-like protein (Fragment) | *Saccharomyces cerevisiae* |
| B5VJA6 | Uncharacterized protein | *Saccharomyces cerevisiae* |
| B5VJ86 | YGR143Wp-like protein (Fragment) | *Saccharomyces cerevisiae* |
| B5VID5 | YGL197Wp-like protein (Fragment) | *Saccharomyces cerevisiae* |
| B5VI39 | Uncharacterized protein | *Saccharomyces cerevisiae* |
| B5VHT3 | YER154Wp-like protein | *Saccharomyces cerevisiae* |
| B5VHS1 | YER145Cp-like protein | *Saccharomyces cerevisiae* |
| B5VHK2 | YER075Cp-like protein | *Saccharomyces cerevisiae* |
| B5VHC3 | YEL013Wp-like protein | *Saccharomyces cerevisiae* |
| B5VHB2 | YEL023Cp-like protein | *Saccharomyces cerevisiae* |
| B5VG58 | Uncharacterized protein | *Saccharomyces cerevisiae* |
| B5VFT6 | YDR033Wp-like protein (Fragment) | *Saccharomyces cerevisiae* |
| B5VFN6 | Uncharacterized protein | *Saccharomyces cerevisiae* |
| B5VFL9 | Uncharacterized protein (Fragment) | *Saccharomyces cerevisiae* |
| B5VEP7 | YCL069Wp-like protein (Fragment) | *Saccharomyces cerevisiae* |
| B5VEF4 | DNA replication licensing factor MCM7 (EC 3.6.4.12) (Fragment) | *Saccharomyces cerevisiae* |
| B5VE61 | YBR098Wp-like protein (Fragment) | *Saccharomyces cerevisiae* |
| B5VDU9 | YBL034Cp-like protein (Fragment) | *Saccharomyces cerevisiae* |
| B8YJG2 | Rhizopuspepsin 1 (EC 3.4.23.21) | *Rhizopus oryzae* |
| P33891 | Protein transport protein TIP20 | *Saccharomyces cerevisiae* |
| P40033 | 37S ribosomal protein RSM18, mitochondrial (Mitochondrial small ribosomal subunit protein bS18m) | *Saccharomyces cerevisiae* |
| P32776 | General transcription and DNA repair factor IIH subunit TFB1 (TFIIH subunit TFB1) (RNA polymerase II transcription factor B 73 kDa subunit) (RNA polymerase II transcription factor B p73 subunit) (RNA polymerase II transcription factor B subunit 1) | *Saccharomyces cerevisiae* |
| C7GXK2 | Ski2p | *Saccharomyces cerevisiae* |
| C7GXM3 | YLR422W-like protein | *Saccharomyces cerevisiae* |
| C7GW03 | Erv41p | *Saccharomyces cerevisiae* |
| C7GS72 | Boi2p | *Saccharomyces cerevisiae* |
| C7GS07 | YJL181W-like protein | *Saccharomyces cerevisiae* |
| C7GNW3 | Mum3p | *Saccharomyces cerevisiae* |
| C7GJY5 | Spo16p | *Saccharomyces cerevisiae* |
| P53043 | Serine/threonine-protein phosphatase T (PPT) (EC 3.1.3.16) | *Saccharomyces cerevisiae* |
| Q01574 | Acetyl-coenzyme A synthetase 1 (EC 6.2.1.1) (Acetate--CoA ligase 1) (Acyl-activating enzyme 1) | *Saccharomyces cerevisiae* |
| C8Z3I8 | EC1118_1A20_0958p | *Saccharomyces cerevisiae* |
| C8Z3Y4 | EC1118_1B15_1145p | *Saccharomyces cerevisiae* |
| C8Z3Z2 | EC1118_1B15_1233p | *Saccharomyces cerevisiae* |
| C8Z465 | EC1118_1C17_0485p | *Saccharomyces cerevisiae* |
| C8Z6C1 | EC1118_1D0_0254p | *Saccharomyces cerevisiae* |
| C8Z4L2 | EC1118_1D0_1728p | *Saccharomyces cerevisiae* |
| C8Z4Q3 | EC1118_1D0_2201p | *Saccharomyces cerevisiae* |
| C8Z4U4 | Fyv1p | *Saccharomyces cerevisiae* |
| C8Z5A9 | EC1118_1D0_4632p | *Saccharomyces cerevisiae* |
| C8Z5E1 | EC1118_1D0_5028p | *Saccharomyces cerevisiae* |
| C8Z5G1 | EC1118_1D0_5248p | *Saccharomyces cerevisiae* |
| C8Z670 | EC1118_1D0_8317p | *Saccharomyces cerevisiae* |
| C8Z7V1 | EC1118_1F14_1101p | *Saccharomyces cerevisiae* |
| C8Z8B2 | EC1118_1G1_1442p | *Saccharomyces cerevisiae* |
| C8Z8G5 | EC1118_1G1_2069p | *Saccharomyces cerevisiae* |
| C8Z8N6 | EC1118_1G1_2905p | *Saccharomyces cerevisiae* |
| C8Z908 | EC1118_1G1_4313p | *Saccharomyces cerevisiae* |
| C8ZAL3 | cAMP-dependent protein kinase regulatory subunit | *Saccharomyces cerevisiae* |
| C8ZCA9 | EC1118_1K5_1827p | *Saccharomyces cerevisiae* |
| C8ZCD3 | EC1118_1K5_2135p | *Saccharomyces cerevisiae* |
| C8ZCU1 | EC1118_1L10_0331p | *Saccharomyces cerevisiae* |
| C8ZDK2 | Lip2p | *Saccharomyces cerevisiae* |
| C8ZGT4 | Dcs2p | *Saccharomyces cerevisiae* |
| C8ZH32 | EC1118_1O4_5138p | *Saccharomyces cerevisiae* |
| C8ZH59 | EC1118_1O4_5468p | *Saccharomyces cerevisiae* |
| C8ZH96 | EC1118_1O4_5908p | *Saccharomyces cerevisiae* |
| C8ZHF1 | EC1118_1O4_6612p | *Saccharomyces cerevisiae* |
| C8ZIV3 | EC1118_1P2_2256p | *Saccharomyces cerevisiae* |
| C8ZJI5 | EC1118_1P2_4995p | *Saccharomyces cerevisiae* |
| Q12745 | Protein transport protein SEC39 (Dependent on SLY1-20 protein 3) | *Saccharomyces cerevisiae* |
| D3J7V5 | Heat shock protein 60 (Fragment) | *Saccharomyces cerevisiae* |
| P38291 | Ribonucleases P/MRP protein subunit POP7 (EC 3.1.26.5) (RNA-processing protein POP7) (RNases P/MRP 15.8 kDa subunit) | *Saccharomyces cerevisiae* |
| P0CE41 | Heme-responsive zinc finger transcription factor HAP1 (CYP1 activatory protein) (Heme activator protein 1) | *Saccharomyces cerevisiae* |
| P0CE00 | Alpha-glucosides permease MPH3 (Maltose transport protein 3) | *Saccharomyces cerevisiae* |
| C8Z4B2 | EC1118_1C17_1002p | *Saccharomyces cerevisiae* |
| C8ZB01 | EC1118_1J11_0100p | *Saccharomyces cerevisiae* |
| C8ZB44 | EC1118_1J11_0606p | *Saccharomyces cerevisiae* |
| D3UF39 | EC1118_1B15_4775p | *Saccharomyces cerevisiae* |
| P0CF21 | Putative truncated L-serine dehydratase YIL168W (EC 4.3.1.17) | *Saccharomyces cerevisiae* |
| A7A0P0 | Guanine nucleotide exchange factor SDC25 | *Saccharomyces cerevisiae* |
| B5VGX4 | Uncharacterized protein | *Saccharomyces cerevisiae* |
| P32523 | Pre-mRNA-processing factor 19 (EC 2.3.2.27) (RING-type E3 ubiquitin transferase PRP19) | *Saccharomyces cerevisiae* |
| E1VCX9 | Replication protein | *Pediococcus pentosaceus* |
| P05759 | Ubiquitin-40S ribosomal protein S31 [Cleaved into: Ubiquitin; 40S ribosomal protein S31 (CEP76) (S37) (Small ribosomal subunit protein eS31) (YS24)] | *Saccharomyces cerevisiae* |
| Q01080 | DNA-directed RNA polymerase I subunit RPA49 (A49) (DNA-directed RNA polymerase I 49 kDa polypeptide) | *Saccharomyces cerevisiae* |
| P40851 | Putative protease AXL1 (EC 3.4.24.-) | *Saccharomyces cerevisiae* |
| O13297 | mRNA-capping enzyme subunit beta (EC 3.1.3.33) (Polynucleotide 5'-triphosphatase) (mRNA 5'-triphosphatase) (TPase) | *Saccharomyces cerevisiae* |
| P35206 | Mannosyl phosphorylinositol ceramide synthase regulatory protein CSG2 | *Saccharomyces cerevisiae* |
| P14922 | General transcriptional corepressor CYC8 (Glucose repression mediator protein CYC8) | *Saccharomyces cerevisiae* |
| P38635 | Histidinol-phosphatase (HolPase) (EC 3.1.3.15) | *Saccharomyces cerevisiae* |
| P53091 | DNA replication licensing factor MCM6 (EC 3.6.4.12) (Minichromosome maintenance protein 6) | *Saccharomyces cerevisiae* |
| P47190 | Dolichyl-phosphate-mannose--protein mannosyltransferase 3 (EC 2.4.1.109) | *Saccharomyces cerevisiae* |
| P13433 | DNA-directed RNA polymerase, mitochondrial (EC 2.7.7.6) | *Saccharomyces cerevisiae* |
| P38714 | Arginine--tRNA ligase, mitochondrial (EC 6.1.1.19) (Arginyl-tRNA synthetase) (ArgRS) | *Saccharomyces cerevisiae* |
| P38623 | Serine/threonine-protein kinase RCK2 (EC 2.7.11.1) (CAM kinase-like protein kinase CLK1) | *Saccharomyces cerevisiae* |
| B5VIR6 | Genetic interactor of prohibitin 7, mitochondrial | *Saccharomyces cerevisiae* |
| P47024 | Transposon Ty4-J Gag-Pol polyprotein (TY4A-TY4B) | *Saccharomyces cerevisiae* |
| P0CL41 | Putative UPF0479 protein YIL177W-A | *Saccharomyces cerevisiae* |
| C8Z3X9 | Vacuolar protein sorting/targeting protein PEP1 (Carboxypeptidase Y receptor) | *Saccharomyces cerevisiae* |
| E7LVV3 | Tethering factor for nuclear proteasome STS1 | *Saccharomyces cerevisiae (strain VIN 13)* |
| P0CY09 | Silenced mating-type protein ALPHA2 (MATalpha2 protein) (Alpha-2 repressor) | *Saccharomyces cerevisiae* |
| P0CX13 | Protein COS3 | *Saccharomyces cerevisiae* |
| P0CX54 | 60S ribosomal protein L12-B (L15) (Large ribosomal subunit protein uL11-B) (YL23) | *Saccharomyces cerevisiae* |
| P0CX22 | Y' element ATP-dependent helicase protein 1 copy 8 (EC 3.6.4.12) | *Saccharomyces cerevisiae* |
| P39714 | (R,R)-butanediol dehydrogenase (EC 1.1.1.4) | *Saccharomyces cerevisiae* |
| Q12389 | ATP-dependent RNA helicase DBP10 (EC 3.6.4.13) (DEAD box protein 10) | *Saccharomyces cerevisiae* |
| P38170 | Condensin complex subunit 2 (Barren homolog) (CAPH homolog) | *Saccharomyces cerevisiae* |
| P27636 | Cell division control protein 15 (EC 2.7.11.1) | *Saccharomyces cerevisiae* |
| P54199 | Serine/threonine-protein kinase MPS1 (EC 2.7.12.2) (Monopolar spindle protein 1) (Regulatory cell proliferation kinase 1) | *Saccharomyces cerevisiae* |
| P39717 | Guanine nucleotide-binding protein subunit beta 2 (Gbeta mimic kelch protein 2) | *Saccharomyces cerevisiae* |
| P38132 | DNA replication licensing factor MCM7 (EC 3.6.4.12) (Cell division control protein 47) (Minichromosome maintenance protein 7) | *Saccharomyces cerevisiae* |
| P38163 | Lethal(2) giant larvae protein homolog SRO77 (Sodium protection protein 2) (Suppressor of RHO3 protein 77) | *Saccharomyces cerevisiae* |
| P29539 | Telomere length regulator protein RIF1 (RAP1-interacting factor 1) | *Saccharomyces cerevisiae* |
| D3UEH0 | Vacuolar membrane protease (EC 3.4.-.-) (FXNA-related family protease 1) | *Saccharomyces cerevisiae* |
| G2HKD7 | K7_07362p | *Saccharomyces cerevisiae* |
| G2HKD8 | Cytochrome c oxidase subunit 1 (EC 1.9.3.1) | *Saccharomyces cerevisiae* |
| G2HKE1 | K7_Ai5_betabp | *Saccharomyces cerevisiae* |
| G2HKF4 | K7_07381p | *Saccharomyces cerevisiae* |
| P46985 | Probable alpha-1,6-mannosyltransferase MNN11 (EC 2.4.1.-) (Mannan polymerase II complex MNN11 subunit) (M-Pol II subunit MNN11) | *Saccharomyces cerevisiae* |
| P53883 | Nucleolar protein 13 | *Saccharomyces cerevisiae* |
| Q08285 | Exosome complex component RRP40 (Ribosomal RNA-processing protein 40) | *Saccharomyces cerevisiae* |
| D6VTK4 | Pheromone alpha factor receptor | *Saccharomyces cerevisiae* |
| P53882 | Topoisomerase I damage affected protein 7 | *Saccharomyces cerevisiae* |
| Q08749 | Mitochondrial import inner membrane translocase subunit TIM18 | *Saccharomyces cerevisiae* |
| P20134 | Flocculation suppression protein (Protein SFL1) | *Saccharomyces cerevisiae* |
| P53167 | tRNA pseudouridine(27/28) synthase (EC 5.4.99.44) (tRNA pseudouridine synthase 2) (tRNA pseudouridylate synthase 2) (tRNA-uridine isomerase 2) | *Saccharomyces cerevisiae* |
| G2W998 | K7_Ybr090cp | *Saccharomyces cerevisiae* |
| G2WD39 | K7_Yer152cp | *Saccharomyces cerevisiae* |
| G2WEK5 | K7_Ygr122wp | *Saccharomyces cerevisiae* |
| G2WFM2 | K7_Skg6p | *Saccharomyces cerevisiae* |
| G2WHA1 | K7_Yjr098cp | *Saccharomyces cerevisiae* |
| G2WHG8 | K7_11205p | *Saccharomyces cerevisiae* |
| G2WIV0 | K7_Mdn1ap (Fragment) | *Saccharomyces cerevisiae* |
| G2WKF9 | K7_Myo5bp (Fragment) | *Saccharomyces cerevisiae* |
| G2WKX3 | K7_Ymr265cp | *Saccharomyces cerevisiae* |
| G2WLD2 | K7_Ami1p | *Saccharomyces cerevisiae* |
| G2WLD5 | K7_Yjr161cp | *Saccharomyces cerevisiae* |
| P38862 | Ubiquitin-like modifier-activating enzyme ATG7 (ATG12-activating enzyme E1 ATG7) (Autophagy-related protein 7) | *Saccharomyces cerevisiae* |
| B5VSC3 | Folylpolyglutamate synthase (EC 6.3.2.17) | *Saccharomyces cerevisiae* |
| P40161 | Histone chaperone RTT106 (Regulator of Ty1 transposition protein 106) | *Saccharomyces cerevisiae* |
| P38850 | Regulator of Ty1 transposition protein 107 (Establishes silent chromatin protein 4) | *Saccharomyces cerevisiae* |
| P33299 | 26S proteasome regulatory subunit 7 homolog (Protein CIM5) (Tat-binding homolog 3) | *Saccharomyces cerevisiae* |
| Q12306 | Ubiquitin-like protein SMT3 | *Saccharomyces cerevisiae* |
| H9BH37 | Sup35 | *Saccharomyces cerevisiae* |
| Q05809 | Cytochrome oxidase assembly factor 4 (Cx9C motif-containing protein 3) | *Saccharomyces cerevisiae* |
| N1P0B2 | Alkyl transferase (EC 2.5.1.-) | *Saccharomyces cerevisiae* |
| N1P056 | Cdc25p | *Saccharomyces cerevisiae* |
| N1NZZ5 | Uncharacterized protein (Fragment) | *Saccharomyces cerevisiae* |
| N1P0G7 | Uncharacterized protein | *Saccharomyces cerevisiae* |
| N1P2W8 | Kkq8p | *Saccharomyces cerevisiae* |
| N1P4B7 | Cos8p | *Saccharomyces cerevisiae* |
| N1P5P5 | Hxt13p | *Saccharomyces cerevisiae* |
| N1P949 | Pad1p | *Saccharomyces cerevisiae* |
| N1P7L8 | Gpb2p | *Saccharomyces cerevisiae* |
| J7FIN6 | Exonuclease V, mitochondrial (Exo V) (EC 3.1.-.-) | *Saccharomyces cerevisiae* |
| J3U3L4 | IRC8 (Fragment) | *Saccharomyces cerevisiae* |
| J7FHZ4 | URN1 | *Saccharomyces cerevisiae* |
| P39935 | Eukaryotic initiation factor 4F subunit p150 (eIF-4F p150) (eIF4F p150) (eIF4G1) (mRNA cap-binding protein complex subunit p150) | *Saccharomyces cerevisiae* |
| A0A0N6X261 | Maturase-like protein (Fragment) | *Saccharomyces cerevisiae* |
| P40302 | Proteasome subunit alpha type-6 (EC 3.4.25.1) (Macropain subunit PRE5) (Multicatalytic endopeptidase complex subunit PRE5) (Proteasome component PRE5) (Proteinase YSCE subunit PRE5) | *Saccharomyces cerevisiae* |
| K4HWN2 | YGL108C-like protein | *Saccharomyces cerevisiae* |
| P11655 | Guanine nucleotide-exchange factor SEC12 (Protein transport protein SEC12) | *Saccharomyces cerevisiae* |
| Q12285 | Ubiquitin-like protein MDY2 (Golgi to ER traffic protein 5) (Mating-deficient protein 2) (Translation machinery-associated protein 24) | *Saccharomyces cerevisiae* |
| P30822 | Exportin-1 (Chromosome region maintenance protein 1) (Karyopherin-124) | *Saccharomyces cerevisiae* |
| A0A0N8VY73 | 50S ribosomal protein L14 | *Pediococcus pentosaceus* |
| Q03EC6 | 50S ribosomal protein L14 | *Pediococcus pentosaceus* |
| A0A199WCI7 | DUF370 domain-containing protein | *Bacillus subtilis subsp. subtilis* |
| A0A1J5X514 | DUF370 domain-containing protein | *Bacillus sp. FMQ74* |
| A0A1Q9FXH7 | DUF370 domain-containing protein | *Bacillus licheniformis* |
| A0A2M8SZZ2 | DUF370 domain-containing protein | *Bacillus sp. SN1* |
| A0A2S4FP16 | DUF370 domain-containing protein | *Bacillus sp. MBGLi97* |
| G4NT01 | YaaB | *Bacillus subtilis subsp. spizizenii (strain TU-B-10)* |
| I0EZ89 | YaaB | *Bacillus sp. JS* |
| M4KMA5 | Uncharacterized protein | *Bacillus subtilis XF-1* |
| P37525 | Extracellular matrix regulatory protein B (Regulator of the extracellular matrix B) | *Bacillus subtilis (strain 168)* |
| Q03HX6 | Bacteriocin-processing peptidase, Cysteine peptidase, MEROPS family C39 | *Pediococcus pentosaceus* |
| Q03H52 | Uncharacterized protein | *Pediococcus pentosaceus* |
| Q03FG5 | Predicted phage phi-C31 gp36 major capsid-like protein | *Pediococcus pentosaceus* |
| A0A061CVE9 | Putative transposase | *Pseudomonas oleovorans* |
| A0A087MGX0 | ATPase AAA | *Arenimonas donghaensis* |
| A0A091BCZ9 | ATPase AAA | *Arenimonas metalli CF5-1* |
| A0A091C2A4 | ATPase AAA | *Arenimonas malthae CC-JY-1* |
| A0A0E3C278 | ATPase AAA | *Comamonas thiooxydans* |
| W6QZ35 | Insertion sequence IS1162 putative ATP-binding protein | *Pseudomonas pseudoalcaligenes* |
| P20434 | DNA-directed RNA polymerases I, II, and III subunit RPABC1 | *Saccharomyces cerevisiae* |
| P22139 | DNA-directed RNA polymerases I, II, and III subunit RPABC5 | *Saccharomyces cerevisiae* |
| P36106 | Transcription factor BYE1 (Bypass of ESS1 protein 1) | *Saccharomyces cerevisiae* |
| P47017 | Sm-like protein LSm1 (SPB8 protein) | *Saccharomyces cerevisiae* |
| P27472 | Glycogen [starch] synthase isoform 2 (EC 2.4.1.11) | *Saccharomyces cerevisiae* |
| P38265 | Inner kinetochore subunit IML3 (CENP-L homolog) | *Saccharomyces cerevisiae* |
| A0A023T460 | Sup35p | *Saccharomyces cerevisiae* |
| Q01454 | DNA polymerase alpha-binding protein (Chromosome replication protein CHL15) | *Saccharomyces cerevisiae* |
| A0A0R1KH24 | Transcription regulator | *Lactobacillus nodensis DSM 19682 = JCM 14932 = NBRC 107160* |
| P53860 | Phosphatidylinositol transfer protein PDR16 (PITP) (Pleiotropic drug resistance protein 16) (SEC14 homolog 3) | *Saccharomyces cerevisiae* |
| A0A0A1HAV5 | RNA dependent RNA polymerase (Fragment) | *Saccharomyces cerevisiae* |
| Q08932 | Ribosome assembly 1 protein | *Saccharomyces cerevisiae* |
| A0A0B5WYD4 | Trehalose 6 phosphate synthase (EC 2.4.1.15) | *Rhizopus oryzae* |
| A0A0C5PRS0 | Putative reverse transcriptase/integrase (Fragment) | *Rhizopus oryzae* |
| A0A0H4BJU5 | Phosphoribosyl anthranilate isomerase | *Saccharomycopsis fibuligera* |
| A0A1Y0VPS6 | Protein-N(Pi)-phosphohistidine--sugar phosphotransferase (EC 2.7.1.191) | *Pediococcus pentosaceus* |
| A0A0R1GZI9 | Resolvase domain protein | *Lactobacillus bifermentans* |
| P39990 | 13 kDa ribonucleoprotein-associated protein (Small nuclear ribonucleoprotein-associated protein 1) | *Saccharomyces cerevisiae* |
| A0A1Y0VM60 | tRNA pseudouridine synthase A (EC 5.4.99.12) (tRNA pseudouridine(38-40) synthase) | *Pediococcus pentosaceus* |
| Q07623 | Nucleolar protein 6 | *Saccharomyces cerevisiae* |
| P52917 | Vacuolar protein sorting-associated protein 4 (DOA4-independent degradation protein 6) | *Saccharomyces cerevisiae* |
| P22219 | Serine/threonine-protein kinase VPS15 (EC 2.7.11.1) (Golgi-retention defective mutant protein 8) (Vacuolar protein sorting-associated protein 15) | *Saccharomyces cerevisiae* |
| Q04781 | E3 ubiquitin-protein ligase listerin (EC 2.3.2.27) (RING domain mutant killed by rtf1 deletion protein 1) (RING-type E3 ubiquitin transferase listerin) | *Saccharomyces cerevisiae* |
| A0A1L4AA40 | Nup116p | *Saccharomyces cerevisiae* |
| A0A1S5VAW4 | Bre5 (Fragment) | *Saccharomyces cerevisiae* |
| A0A1Y0VKD7 | CagE_TrbE_VirB domain-containing protein | *Pediococcus pentosaceus* |
| A0A1Y0VTU7 | Uncharacterized protein | *Pediococcus pentosaceus* |
| A0A1Y0VUN0 | Putative ABC transporter ATP-binding protein | *Pediococcus pentosaceus* |
| A0A1Y0VUB3 | UmuC domain-containing protein | *Pediococcus pentosaceus* |
| A0A1Y0VNM3 | Protein smf | *Pediococcus pentosaceus* |
| A0A1Y0VQA1 | Usp domain-containing protein | *Pediococcus pentosaceus* |
| P16521 | Elongation factor 3A (EF-3) (EF-3A) (Eukaryotic elongation factor 3) (eEF3) (Translation elongation factor 3A) | *Saccharomyces cerevisiae* |
| P06786 | DNA topoisomerase 2 (EC 5.6.2.2) (DNA topoisomerase II) | *Saccharomyces cerevisiae* |
| P32389 | Transcriptional activator of sulfur metabolism MET4 (Methionine-requiring protein 4) | *Saccharomyces cerevisiae* |
| Q00955 | Acetyl-CoA carboxylase (ACC) (EC 6.4.1.2) (Fatty acid synthetase 3) (mRNA transport-defective protein 7) | *Saccharomyces cerevisiae* |
| Q06287 | Ribosomal RNA small subunit methyltransferase NEP1 (EC 2.1.1.260) | *Saccharomyces cerevisiae* |
| Q05021 | Transcription initiation factor TFIID subunit 7 (TAFII-67) | *Saccharomyces cerevisiae* |
| Q00582 | GTP-binding protein GTR1 | *Saccharomyces cerevisiae* |
| P49687 | Nucleoporin NUP145 (EC 3.4.21.-) (Nuclear pore protein NUP145) | *Saccharomyces cerevisiae* |
| Q04491 | Protein transport protein SEC13 | *Saccharomyces cerevisiae* |
| P48305 | NADH dehydrogenase [ubiquinone] 1 beta subcomplex subunit 4 (Complex I-B15) (CI-B15) (NADH-ubiquinone oxidoreductase B15 subunit) | *Bos taurus (Bovine)* |
| F7VJM9 | Transition protein 1 | *Bos taurus (Bovine)* |
| P06701 | Regulatory protein SIR3 (Silent information regulator 3) | *Saccharomyces cerevisiae* |
| P46673 | Nucleoporin NUP85 (Nuclear pore protein NUP85) | *Saccharomyces cerevisiae* |
| P36136 | Sedoheptulose 1,7-bisphosphatase (EC 3.1.3.37) | *Saccharomyces cerevisiae* |
| P03069 | General control protein GCN4 (Amino acid biosynthesis regulatory protein) | *Saccharomyces cerevisiae* |
| Q07794 | Histone acetyltransferase RTT109 (EC 2.3.1.48) (Regulator of Ty1 transposition protein 109) | *Saccharomyces cerevisiae* |
| P38011 | Guanine nucleotide-binding protein subunit beta-like protein (Receptor for activated C kinase) (Receptor of activated protein kinase C 1) (RACK1) (Small ribosomal subunit protein RACK1) | *Saccharomyces cerevisiae* |
| P07273 | Transcription elongation factor S-II (DNA strand transfer protein alpha) (STP-alpha) (DNA strand transferase 1) (Pyrimidine pathway regulatory protein 2) | *Saccharomyces cerevisiae* |
| P32581 | Meiosis induction protein kinase IME2/SME1 (EC 2.7.11.1) | *Saccharomyces cerevisiae* |
| P46675 | Protein STU2 (Suppressor of tubulin 2) | *Saccharomyces cerevisiae* |
| P32618 | Uncharacterized protein YEL043W | *Saccharomyces cerevisiae* |
